# Flare-productive active regions

**DOI:** 10.1007/s41116-019-0019-7

**Published:** 2019-05-21

**Authors:** Shin Toriumi, Haimin Wang

**Affiliations:** 1 Institute of Space and Astronautical Science (ISAS)/Japan Aerospace Exploration Agency (JAXA), 3-1-1 Yoshinodai, Chuo-ku, Sagamihara, Kanagawa 252-5210 Japan; 2 National Astronomical Observatory of Japan, 2-21-1 Osawa, Mitaka, Tokyo 181-8588 Japan; 3 Institute for Space Weather Sciences, New Jersey Institute of Technology, University Heights, Newark, NJ 07102-1982 USA; 4 Big Bear Solar Observatory, New Jersey Institute of Technology, 40386 North Shore Lane, Big Bear City, CA 92314-9672 USA

**Keywords:** Active regions, Magnetic fields active regions, Structure coronal mass ejections, Initiation and propagation flares, Dynamics flares, Models magnetohydrodynamics

## Abstract

Strong solar flares and coronal mass ejections, here defined not only as the bursts of electromagnetic radiation but as the entire process in which magnetic energy is released through magnetic reconnection and plasma instability, emanate from active regions (ARs) in which high magnetic non-potentiality resides in a wide variety of forms. This review focuses on the formation and evolution of flare-productive ARs from both observational and theoretical points of view. Starting from a general introduction of the genesis of ARs and solar flares, we give an overview of the key observational features during the long-term evolution in the pre-flare state, the rapid changes in the magnetic field associated with the flare occurrence, and the physical mechanisms behind these phenomena. Our picture of flare-productive ARs is summarized as follows: subject to the turbulent convection, the rising magnetic flux in the interior deforms into a complex structure and gains high non-potentiality; as the flux appears on the surface, an AR with large free magnetic energy and helicity is built, which is represented by $\delta$-sunspots, sheared polarity inversion lines, magnetic flux ropes, etc; the flare occurs when sufficient magnetic energy has accumulated, and the drastic coronal evolution affects magnetic fields even in the photosphere. We show that the improvement of observational instruments and modeling capabilities has significantly advanced our understanding in the last decades. Finally, we discuss the outstanding issues and future perspective and further broaden our scope to the possible applications of our knowledge to space-weather forecasting, extreme events in history, and corresponding stellar activities.

**Electronic supplementary material:**

The online version of this article (10.1007/s41116-019-0019-7) contains supplementary material, which is available to authorized users.

## Introduction

Ever since sunspot observations with telescopes started in the beginning of seventeenth century, vast amounts of observational data have been collected. Triggered by the momentous discovery of solar flares by Carrington (1859) and Hodgson (1859) and by the report of the existence of magnetic fields in sunspots by Hale (1908), the close relationship between the production of solar flares and the magnetism of active regions (ARs) has been extensively argued.

Advances in ground-based and space-borne telescopes have accelerated this trend. In recent decades, new instruments such as Hinode (Kosugi et al. 2007), Solar Dynamics Observatory (SDO; Pesnell et al. 2012), and the Goode Solar Telescope (GST; Cao et al. 2010)^1^ have delivered rich observational information and enabled us to study flares and ARs in unprecedented detail. Moreover, the ever-increasing capability of numerical simulations performed on supercomputers has improved the advanced modeling of these phenomena and deepened our understanding of their physical background.

From experience we know that there are flare-productive and flare-quiet ARs. Then, some of the key questions are:

- What are the important morphological and magnetic properties of the flare-productive ARs that differentiate these from flare-quiet ARs?
- What are the key observational features that are created during the course of large-scale, long-term AR evolution?
- What subsurface dynamics and physical mechanisms produce such observed properties and features?
- What rapid changes occur in magnetic fields during the flare eruptions?

The understanding of the flaring of ARs is not only motivated by academic curiosity but also desired by the practical demand of space weather forecasts that is growing more rapidly than ever before. Needless to say, the flaring activity of our host star directly affects the condition of the near-Earth environment through emitting coronal mass ejections (CMEs), electromagnetic radiation, and high energy particles.^2^ As the successful detection of stellar flares and starspots of solar-like stars is now increasing more and more, it is a key remaining issue for solar physicists to reveal the conditions of strong flare eruptions based on the rich information of solar ARs and flares.

Therefore, we set as primary aim of this review article the summary of the current understanding of the formation and evolution of flare-productive ARs that has been brought about through decades of effort of observational and theoretical investigations. For this aim, we first highlight key observational properties of flaring ARs during the course of long-term and large-scale evolution. We then proceed to the theoretical studies that try to understand the physical origins of these observed properties. We switch our focus to the drastic evolution during the main stage of the flare and discuss the possibility that the changes in coronal fields affect the photospheric conditions. After we summarize what we have learned so far, especially in the age with Hinode, SDO, and GST, our discussion extends further to the possibilities of space weather forecasting and historical data analysis and even to the connection with stellar flares and CMEs. Although we carefully avoid stepping into the details too much, we provide references to excellent reviews since the main topic of this article, i.e., the development of flaring ARs, is closely related to a wide spectrum of phenomena from solar dynamo, flux emergence and AR formation to sunspots, flares and CMEs.

The rest of this article is structured as follows. Section 2 provides the general introduction to the AR formation, solar flares and CMEs, and their relationships. Section 3 reviews the key morphological and magnetic properties of flare-productive ARs that are observed during the long-term and large-scale evolution. Then, in Sect. 4, we show the theoretical and numerical attempts to model and understand how these properties are created. Section 5 is dedicated to the discussion on rapid changes associated with flare eruptions. Finally, the summary and discussion are given in Sects. 6 and 7, respectively.

**Fig. 1:**
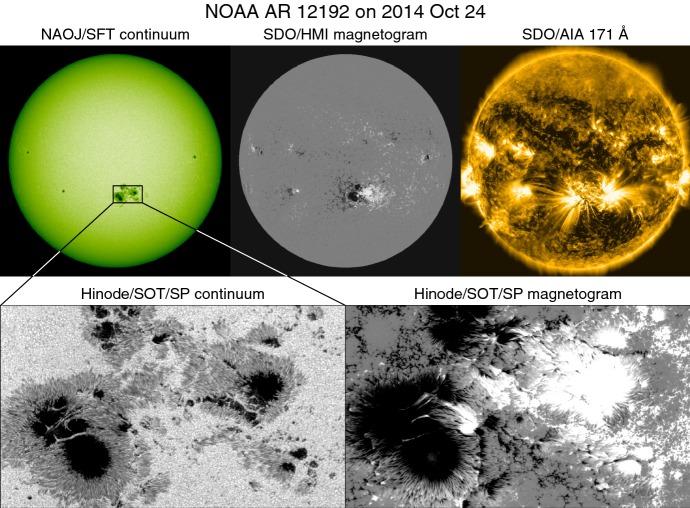
Huge flare-productive AR NOAA 12192. Images are obtained by the SDO and Hinode satellites as well as the Solar Flare Telescope in NAOJ

## Active regions and solar flares

Figure 1 shows example images of the Sun. In the southern hemisphere, one may find a large sunspot group (top left: surrounded by a box), in which the magnetic field is strongly concentrated (top middle: magnetogram by SDO’s Helioseismic and Magnetic Imager (HMI); Scherrer et al. 2012; Schou et al. 2012) and the bright loop structures are clearly seen in the EUV image (top right: 171 Å channel of SDO’s Atmospheric Imaging Assembly (AIA); Lemen et al. 2012). This region, numbered 12192 by National Oceanic and Atmospheric Administration (NOAA), appeared in October 2014 as one of the largest spot groups ever observed with a maximum spot area of 2750 MSH^3^ and produced numerous solar flares including six X-class events on the Geostationary Operational Environmental Satellite (GOES) scale. These centers of activity are called ARs (see van Driel-Gesztelyi and Green 2015, for the history of the definition of ARs). In the simplest cases, ARs take a form of a simple bipole structure. However, as the detailed observation by Hinode’s Solar Optical Telescope (SOT; Tsuneta et al. 2008) shows, ARs are sometimes composed of a number of magnetic elements of various size scales (bottom panels), and the flare productivity is known to increase with the “complexity” of the ARs.

In this section, we introduce the present knowledge of how the ARs and sunspots are generated, how they become unstable and produce flares and CMEs, and how these features, i.e., the spots and flares, are related.

### Flux emergence and AR formation

It is generally thought that ARs are created as a result of the emergence of toroidal magnetic flux from the deeper convection zone (flux emergence: Parker 1955; Babcock 1961). In most dynamo models (Charbonneau 2010; Brun and Browning 2017), the toroidal flux is generated and amplified by turbulence and shear in the tachocline, the thin shear layer at the base of the solar convection zone. There are alternative possibilities such as the dynamo working in the near surface shear layer (Brandenburg 2005) and the amplification of advected horizontal fields by convection (Stein and Nordlund 2012). Magnetic flux systems created through these processes emerge to the solar surface and eventually generate ARs.

Below we introduce the emergence processes in the interior and to the atmosphere from both theoretical and observational viewpoints. For more comprehensive discussion, interested readers may also consult the review papers by Fisher et al. (2000), Charbonneau (2010) and Brun and Browning (2017) that are specialized in magnetism in the solar interior, Zwaan (1985) and van Driel-Gesztelyi and Green (2015) for observational properties and Archontis (2008), Fan (2009a), Cheung and Isobe (2014) and Schmieder et al. (2014) that elaborate on theories and models of flux emergence.

#### Emergence in the interior: theory

Parker (1955) demonstrated that a horizontal flux tube, a horizontal bundle of magnetic field lines, will rise due to magnetic buoyancy. Let us assume pressure balance between inside and outside the thin flux tube,

1 $$\begin{aligned} p_{\mathrm{e}}=p_{\mathrm{i}}+\frac{B^{2}}{8\pi }, \end{aligned}$$

where $p_{\mathrm{i}}$ and $p_{\mathrm{e}}$ are the pressure inside and outside the flux tube, whose average field strength is *B*. When the plasma is in local thermodynamic equilibrium, i.e., $T_{\mathrm{e}}=T_{\mathrm{i}}=T$, the above equation can be rewritten as

2 $$\begin{aligned} \rho _{\mathrm{e}}=\rho _{\mathrm{i}}+\frac{B^{2}}{8\pi }\frac{m}{k_{\mathrm{B}}T}, \end{aligned}$$

where $\rho$ is the density, *m* mean molecular mass, and $k_{\mathrm{B}}$ the Boltzmann constant. It is obvious from this equation that the flux tube is buoyant ($\rho _{\mathrm{i}}<\rho _{\mathrm{e}}$), and the buoyancy per unit volume is

3 $$\begin{aligned} f_{\mathrm{B}}=(\rho _{\mathrm{e}}-\rho _{\mathrm{i}})g =\frac{B^{2}}{8\pi }\frac{mg}{k_{\mathrm{B}}T} =\frac{B^{2}}{8\pi H_{\mathrm{p}}}, \end{aligned}$$

where $H_{\mathrm{p}}=k_{\mathrm{B}}T/(mg)$ is the local pressure scale height.

In most parts of the interior, the plasma-$\beta$ ($\equiv 8\pi p/B^{2}$) is (much) greater than unity. For a magnetic flux at the base of the convection zone with a field strength of $10^{5}$ G, which is 10 times stronger than the field strength that is in equipartition with the local kinetic energy density, the plasma-$\beta$ is of the order of $10^{5}$ (e.g., Fan 2009a). In such a situation, the rising flux can still be affected by external flow fields of thermal convection.

A large number of numerical models have been developed and revealed various physical mechanisms of flux emergence and observed AR characteristics. For example, magnetohydrodynamic (MHD) simulations show that a horizontal magnetic layer at the base of the convection zone in mechanical equilibrium can break up and develop into buoyant magnetic flux tubes through the magnetic buoyancy instability (Cattaneo and Hughes 1988; Matthews et al. 1995; Fan 2001a). In order to keep the flux tube coherent, it was suggested that the flux tube needs twist, i.e., the azimuthal component of the magnetic field should wrap around the tube’s axis (Parker 1979a; Longcope et al. 1996; Moreno-Insertis and Emonet 1996). Abbett et al. (2000) found that, in 3D simulations, the amount of twist necessary for the tube to retain its coherency is reduced substantially comparing to the 2D limit.

**Fig. 2:**
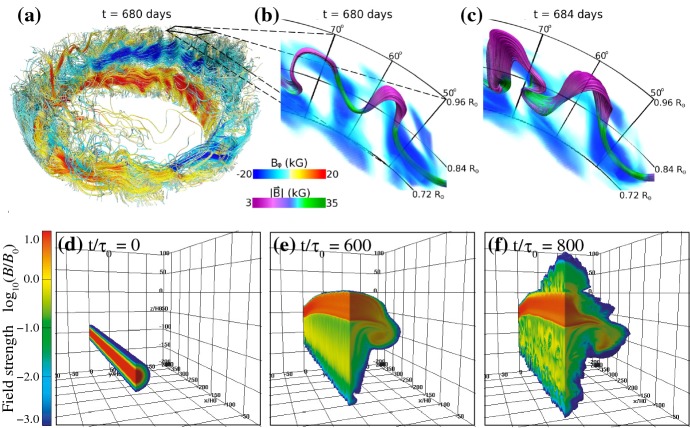
**a**–**c** Emergence of buoyant $\varOmega$-loops from a magnetic wreath self-consistently generated in an anelastic dynamo model. Panels **b** and **c** demonstrate the local evolution within a domain extending from $0.72\,R_{\odot }$ ($-\,195\, \mathrm{Mm}$ from the solar surface) to $0.96\,R_{\odot }$ ($-\,28\, \mathrm{Mm}$), with volume rendering indicating the toroidal field strength. Image reproduced by permission from Nelson et al. (2013), copyright by AAS. **d**–**f** Flux emergence simulation in a single computational domain that seamlessly covers from the convection zone to the corona with a vertical extent from $-\,40$ to $+\,50\, \mathrm{Mm}$ (here shown up to $+\,20\, \mathrm{Mm}$). The rising flux tube, initially placed at $-\,20\, \mathrm{Mm}$, decelerates and expands horizontally before it appears on the photosphere and erupts into the corona. Normalizing units are $H_{0}=200\, \mathrm{km}$ for length, $\tau _{0}=25\, \mathrm{s}$ for time, and $B_\mathrm{0}=300\, \mathrm{G}$ for magnetic field strength. Image reproduced by permission from Toriumi and Yokoyama (2012), copyright by ESO

The effect of the Coriolis force on the rising flux tube, including the asymmetry between the leading and following spots of bipolar ARs, has been studied by simulations with the assumption that the flux tube is thin enough that the cross sectional evolution can be neglected (thin flux tube approximation: e.g., Spruit 1981; Choudhuri and Gilman 1987; Fan et al. 1993; D’Silva and Choudhuri 1993; Caligari et al. 1995). The emergence in the convective interior and its interaction with the flow fields have been considered in simulations that apply the anelastic MHD approximation (e.g., Gough 1969; Fan et al. 2003; Fan 2008; Jouve and Brun 2009; Nelson et al. 2011; Weber et al. 2011; Jouve et al. 2013). The top panels of Fig. 2 illustrate the anelastic simulation by Nelson et al. (2013), who modeled the buoyant rise of $\varOmega$-shaped loops generated self-consistently from a bundle of toroidal flux (magnetic wreath).

However, these assumptions become inappropriate in the uppermost convection zone above a depth of about $20\, \mathrm{Mm}$ (Fan 2009a). This difficulty motivated Toriumi and Yokoyama (2010, 2011) to conduct fully-compressible MHD simulations that seamlessly connect the different atmospheric layers from a depth of $40\, \mathrm{Mm}$ in the interior to the solar corona. They found that, as illustrated in 3D models in Fig. 2d–f, the rising flux tube, starting at $-\,20\, \mathrm{Mm}$, temporarily slows down and undergoes horizontal expansion (pancaking) while generating escaping plasma flows before it resumes emergence into the photosphere and beyond. This process, termed “two-step emergence,” is widely observed in the larger-scale models from the interior to the atmosphere (see Sect. 3.3.5 of Cheung and Isobe 2014). As an alternative approach, Abbett and Fisher (2003) and Chen et al. (2017) joined global-scale anelastic models and local MHD simulations from the near-surface layer upwards and investigated fuller history of emergence.

#### Emergence in the interior: observation

Several attempts have been made to detect the subsurface emerging magnetic flux using local helioseismology (see review by Gizon and Birch 2005). One of the earliest works, Braun (1995), reported on the p-mode scattering starting about 2 days before the spot formation in the emerging AR NOAA 5247. The following case studies mainly focused on the wave-speed perturbation and subsurface flow fields before the flux appearance: Chang et al. (1999), Jensen et al. (2001), Komm et al. (2008), Kosovichev and Duvall (2008), Zharkov and Thompson (2008) and Kosovichev (2009). However, in most cases, it was difficult to detect significant seismic signatures associated with the emerging flux, probably because of the fast rising motion and accordingly short observation time, which leads to low signal-to-noise ratio.

A recent observation by Ilonidis et al. (2011), however, detected strong seismic perturbations in NOAA 10488 at depths between 42 and 75 Mm, up to 2 days before the photospheric flux reaches its maximum flux growth rate. The estimated rising speed from 65 Mm to the surface is about $0.6\, \mathrm{km\ s}^{-1}$ (see also Braun 2012; Ilonidis et al. 2013; Kholikov 2013; Kosovichev et al. 2018). Statistical studies by Komm et al. (2009, 2011b, 2012) showed indications of upflows, rotations, and increased vorticity in the subsurface layer. Leka et al. (2013), Birch et al. (2013) and Barnes et al. (2014) analyzed more than 100 emerging regions and found that there are statistically significant seismic signatures in average subsurface flows and the apparent wave speed, at least one day prior to the emergence, although their individual samples did not show discernible signal greater than the noise level.

Other possible precursors of flux emergence on the surface are the reduction in acoustic oscillation power (Hartlep et al. 2011; Toriumi et al. 2013b), f-mode amplification (Singh et al. 2016), and horizontal divergent flows (Toriumi et al. 2012, 2014a).

#### Birth of ARs: observation

**Fig. 3:**
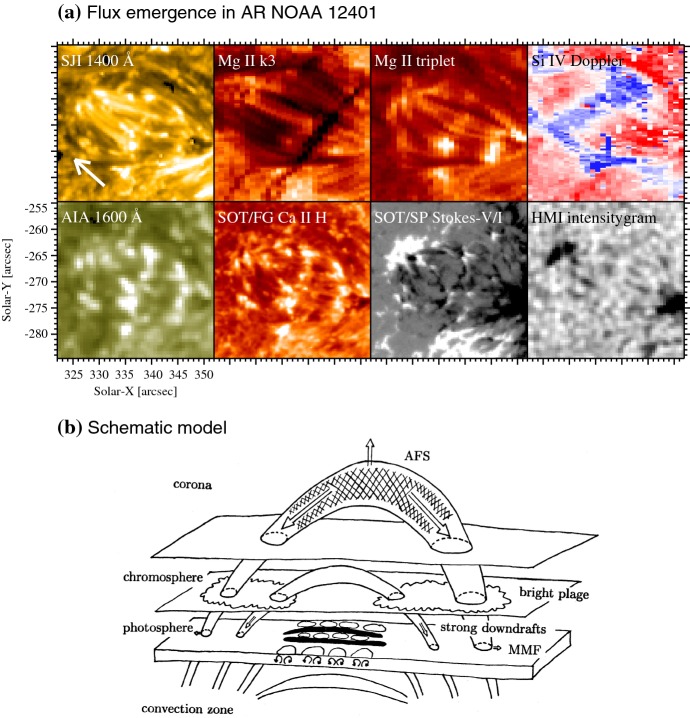
**a** “Textbook” flux emergence in AR NOAA 12401 observed simultaneously by Hinode, the Interface Region Imaging Spectrograph (IRIS; De Pontieu et al. 2014), and SDO (2015 August 19). From top left to bottom right are the IRIS slit-jaw image of 1400 Å, raster-scan intensitygram at the Mg ii k line core (k3: 2796 Å), intensitygram at the Mg ii triplet line (2798 Å), Dopplergram produced from the Si iv 1403 Å spectrum (blue, white, and red correspond to $-\,10$, 0, and $+\,40\, \mathrm{km\ s}^{-1}$, respectively), SDO/AIA 1600 Å, Hinode/SOT/FG Ca ii H, SOT/SP Stokes-V/I, and SDO/HMI intensitygram. The white arrow in the top left panel indicates the direction of the disk center. In the accompanying movie, the Ca ii H and Stokes-V/I maps are replaced by the AIA 1700 Å image and HMI magnetogram, respectively. (For movie see Electronic Supplementary Material.) Image and movie reproduced by permission from Toriumi et al. (2017a), copyright by AAS. **b** Schematic model of flux emergence. Image reproduced by permission from Shibata et al. (1989), copyright by AAS. The original version of this illustration appeared in Shibata’s review note in 1979

As the rising magnetic flux reaches the photosphere, it starts to build up an AR if the flux is sufficiently large. Figure 3a and its accompanying movie show various aspects of a newly emerging flux region. In a magnetogram (Stokes-V/I map), the emerging flux is scattered throughout the region as a number of small-scale magnetic elements of positive and negative polarities. These elements merge with and cancel each other in the middle of the region and gradually form pores and, if the emerged flux is sufficient, they eventually create sunspots (Zwaan 1978). Zwaan (1985) introduced the hierarchy of magnetic elements. Sunspots with a flux of $5\times 10^{20}\, \mathrm{Mx}$ or more have a penumbra and the umbral field is 2900–$3300\, \mathrm{G}$, sometimes exceeding $4000\, \mathrm{G}$, while the flux of pores is $2.5\times 10^{19}$–$5\times 10^{20}\, \mathrm{Mx}$ and the field strength is ${\sim }\,2000\, \mathrm{G}$. If the flux is less than $10^{20}\, \mathrm{Mx}$, the emerging regions do not develop beyond ephemeral regions (Harvey and Martin 1973).

From the observation of repeated emergence and cancellation of photospheric magnetic elements, Strous et al. (1996) and Strous and Zwaan (1999) suggested that this behavior is due to the rising of undulatory (sea-serpent) field lines. Georgoulis et al. (2002), Bernasconi et al. (2002) and Pariat et al. (2004) suggested that Ellerman bombs, the bursty intensity enhancements in H$\alpha$ line wings (Ellerman 1917), are located at the dipped parts, at which magnetic reconnection takes place to disconnect emerged flux from un-emerged, mass-laden parts of the flux tube (resistive emergence model). UV bursts in the transition region lines are similarly found at the cancellation sites (Peter et al. 2014; Young et al. 2018). Brightenings seen in 1400 Å, 1600 Å, and Ca ii H of Fig. 3a correspond to Ellerman bombs and UV bursts.

Soon after the magnetic flux shows up, an arch filament system (AFS) appears as parallel dark fibrils, probably the manifestation of rising magnetic fields (Bruzek 1967, 1969, see Mg ii k3 image of Fig. 3a). Bipolar plages are observed in the chromospheric Ca ii H and K lines at the footpoints of the AFS (Kawaguchi and Kitai 1976, brightenings above the pores in Fig. 3a). The Hinode analysis of AFS by Otsuji et al. (2007, 2010) shows the horizontal expansion and upward acceleration of emerging flux, which strongly supports the “two-step emergence” scenario (Sect. 2.1.1). The observational characteristics of emerging flux regions are schematically summarized by Shibata et al. (1989) as an illustration in Fig. 3b.

#### Birth of ARs: theory

The MHD modeling of flux emergence from the photospheric layer to the corona was pioneered by Shibata et al. (1989), who simulated the 2D emergence due to the Parker instability, the undular mode of the magnetic buoyancy instability (Parker 1979a). They successfully reproduced the observed dynamical features such as rising motion of the AFS and the strong downflow along the field lines. Since then, the flux emergence process has been widely studied both in 2D and 3D (e.g., Shibata et al. 1990; Kaisig et al. 1990; Nozawa et al. 1992; Magara 2001; Matsumoto and Shibata 1992; Matsumoto et al. 1993; Fan 2001b; Magara and Longcope 2001; Archontis et al. 2004; Isobe et al. 2005; Murray et al. 2006).

**Fig. 4:**
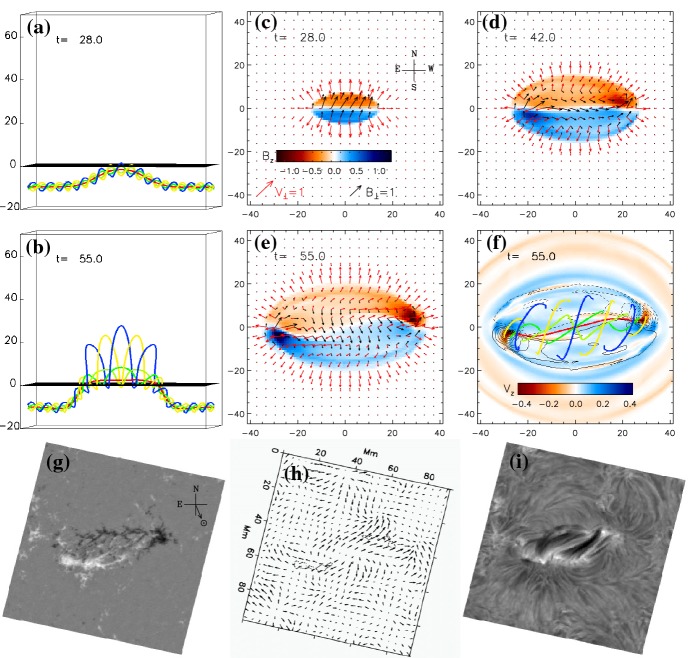
3D flux emergence simulation from around the photospheric height. **a**, **b** Selected field lines of the emerging flux tube. **c**–**e** Vertical magnetic field $B_{z}$, the horizontal magnetic field (black arrows), and the horizontal velocity field (red arrows). **f** Top-down view of panel **b** with vertical velocity $v_{z}$. **g**–**i** Line-of-sight (LOS) magnetic field, horizontal velocity, and H$\alpha$ image of NOAA AR 5617, respectively. Image reproduced by permission from Fan (2001b), copyright by AAS

Figure 4 shows a typical example of flux emergence simulations by Fan (2001b), which models the buoyant rise of a twisted flux tube from just beneath the photosphere ($-\,1.5\, \mathrm{Mm}$) and upwards. The initial flux tube, which is horizontal and endowed with a density deficit at the middle with respect to the surroundings, starts rising due to the magnetic buoyancy and deforms into an $\varOmega$-loop (panel a). As the flux tube penetrates into the upper atmosphere, a ying–yang pattern of positive and negative polarities (vertical field $B_{z}$) is produced in the photosphere (panels c–e), which resembles the polarity layout in the actual AR (panel g). Due to the initial twist, magnetic field lines in the atmosphere show a twisted structure, which also mimics the observed helical nature of the AFS (panel i).

Forbes and Priest (1984) and Yokoyama and Shibata (1995, 1996) investigated the interaction between emerging flux and the preexisting coronal loop (the model proposed by Heyvaerts et al. 1977) and successfully reproduced jet ejections (see also Miyagoshi and Yokoyama 2003; Moreno-Insertis et al. 2008; Nishizuka et al. 2008; Murray et al. 2009; Archontis et al. 2010; Takasao et al. 2013; Moreno-Insertis and Galsgaard 2013). Magnetic flux cancellation at the emerging undular fields and the resultant production of Ellerman bombs were modeled by Isobe et al. (2007) in 2D and Archontis and Hood (2009) in 3D.

With the growing ability of computation resources, simulations have become more realistic and now take into account the effect of thermal convection on flux emergence. For instance, Cheung et al. (2008) performed 3D radiative MHD simulations of the emergence of an initially horizontal flux tube in the granular convection. They found that, due to vigorous convective flows at the top of the convection zone, the rising tube is highly structured by the surface granulation pattern, which is well in agreement with the Hinode/SOT observations. The series of numerical simulations of similar setups consistently showed that the granular cells are expanded and elongated as the horizontal flux approaches and that the surface convection makes undular field lines (dipped field at the downflow lanes), which reconnect with each other and drain down the plasma from the surface layer (Abbett 2007; Cheung et al. 2007; Isobe et al. 2008; Martínez-Sykora et al. 2008, 2009; Tortosa-Andreu and Moreno-Insertis 2009; Fang et al. 2010). The realistic modeling by Archontis and Hansteen (2014) and Hansteen et al. (2017) successfully reproduced the small-scale reconnection events at the dipped fields and showed that they can be observed as Ellerman bombs or UV bursts depending on the reconnection heights. Throughout these processes, the magnetic elements grow larger and, eventually, the sunspots are formed (Cheung et al. 2010; Rempel and Cheung 2014).

### Solar flares and CMEs

**Fig. 5:**
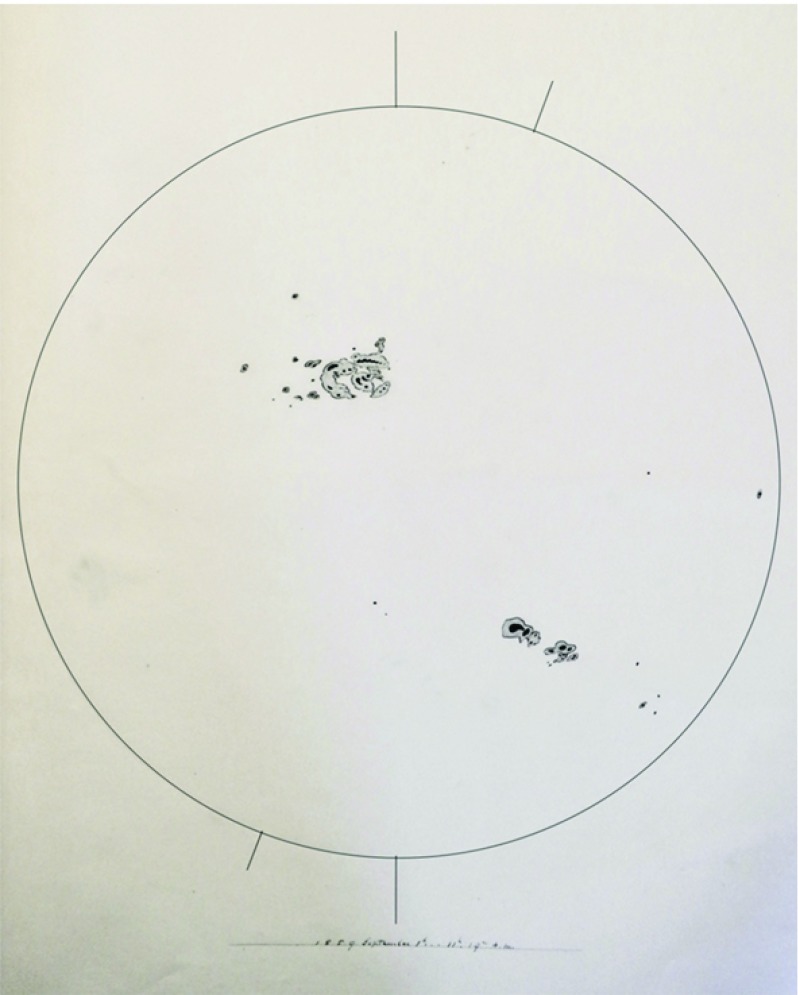
Carrington’s original whole-disk drawing on 1859 September 1. Carrington (1859) and Hodgson (1859) observed the white light flare in the large sunspot region in the northern hemisphere. This manuscript is currently preserved in the archive of the Royal Astronomical Society (RAS) as RAS MSS Carrington 3.2: Drawings of sunspots, showing the whole of the Sun’s disk, v.2, f.313a. For a better visualization, the thickness of the limb and axes is enhanced. Image reproduced by permission from Hayakawa et al. (2018), copyright by AAS and RAS

In most astronomical contexts, the term “flare” refers to the abrupt increase in intensity of electromagnetic waves, and the flares on the Sun are detected over a wide range of spectrum such as X-rays, (E)UV, radio, and even white light. In fact, the discovery of flares was made as a remarkable intensity enhancement in white light (Carrington event on 1859 September 1; Carrington 1859; Hodgson 1859). Figure 5 is the original whole-disk drawing by Carrington, which shows a large spot group that produced the strong white light flare. Nowadays, flare strengths are grouped by peak soft X-ray flux over 1–8 Å, measured by GOES, into logarithmic classes A, B, C, M, X, corresponding to $10^{-8}$, $10^{-7}$, $10^{-6}$, $10^{-5}$, $10^{-4}\, \mathrm{W\ m}^{-2}$ at Earth, respectively, so X1.2 and M3.4 represent $1.2\times 10^{-4}\, \mathrm{W\ m}^{-2}$ and $3.4\times 10^{-5}\, \mathrm{W\ m}^{-2}$, respectively. The Carrington flare is arguably considered as the most powerful event ever with the estimated magnitude of X45 ($\pm \, 5$) and bolometric energy of $5\times 10^{32}\, \mathrm{erg}$ (Tsurutani et al. 2003; Cliver and Svalgaard 2004; Boteler 2006; Cliver and Dietrich 2013).

**Fig. 6:**
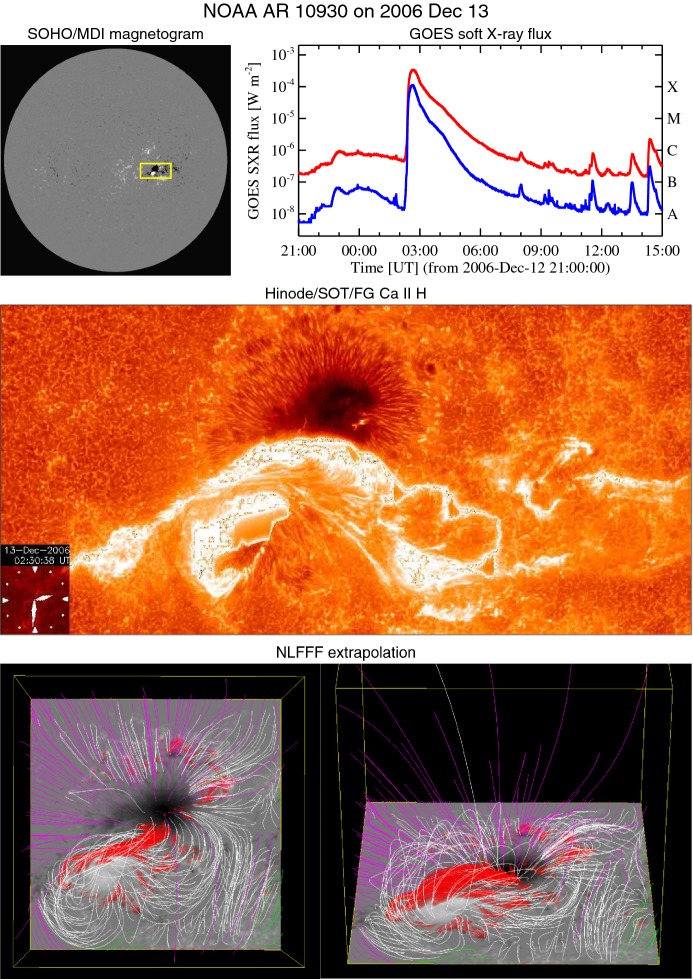
X3.4-class flare in AR NOAA 10930. The panels show full-disk magnetogram from Michelson Doppler Imager (MDI) aboard the Solar and Heliospheric Observatory (SOHO), GOES soft X-ray light curves for 1–8 Å (red) and 0.5–4.0 Å (blue), and Hinode/SOT/FG Ca ii H image (see also the accompanying movie), whose FOV is indicated by a yellow box in the magnetogram. Hinode image courtesy of Joten Okamoto (ISAS/JAXA and NAOJ). The bottom panel displays the computationally extrapolated magnetic field lines before the X3.4 flare using the NLFFF method. The red isosurface shows where the electric current is highest. Image reproduced by permission from Schrijver et al. (2008), copyright by AAS

Solar flares are now considered as the conversion process of (free) magnetic energy to kinetic and thermal energy as well as particle acceleration, most probably through magnetic reconnection. Figure 6 shows the GOES X3.4-class flare in AR NOAA 10930. From this figure and the corresponding movie, one may find that the flare occurs between the two major sunspots, particularly at the polarity inversion line (PIL: also called the neutral line), where the vertical field $B_{z}$ or the line-of-sight (LOS) field $B_{\mathrm{LOS}}$ remains zero and the sign flips across it. The most pronounced feature is the pair of flare ribbons that spreads along and away from the PIL (Bruzek 1964; Asai et al. 2004). The magnetic field in the corona, which is computationally extrapolated from the photospheric magnetogram using the non-linear force-free field (NLFFF) method (Sect. 4.3.1), shows a helical topology above the PIL. Such a highly non-potential, twisted magnetic structure called a magnetic flux rope is often observed in soft X-rays prior to the flare occurrence (see Sect. 3.3.1).

**Fig. 7:**
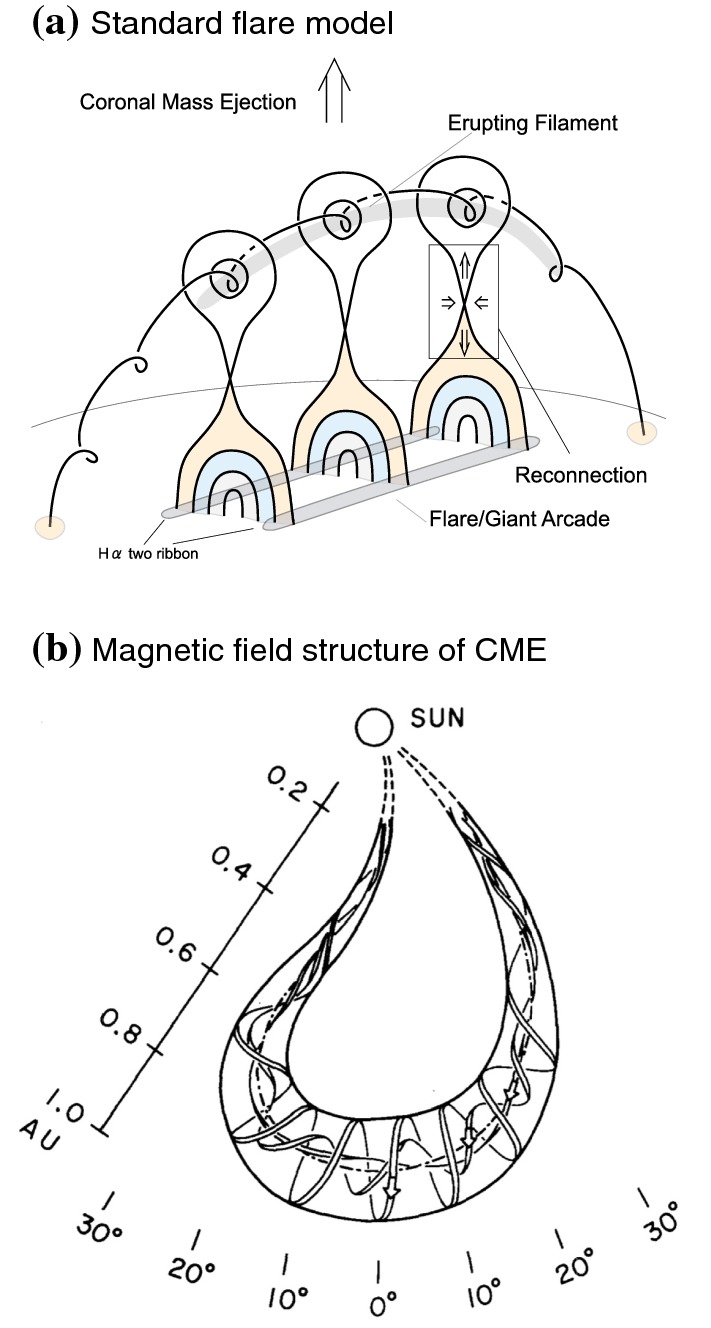
**a** Schematic illustration of the standard flare model. Image reproduced by permission from Shiota et al. (2005), copyright by AAS. The thick solid lines represent magnetic field lines. Shaded, hatched, and dotted regions display the features observed in soft X-rays, EUV, and H$\alpha$, respectively. **b** Observationally inferred magnetic field structure of CMEs in the interplanetary space. Image reproduced by permission from Marubashi (1989), copyright by Kluwer

Various observational characteristics of the flares, not only the ribbons and the flux rope but also the cusp-shaped loops seen in soft X-rays (Tsuneta et al. 1992), hard X-ray loop-top source (Masuda et al. 1994), inflows toward a current sheet (Yokoyama et al. 2001), etc., altogether lend support to the well-established flare model based on the magnetic reconnection scenario, referred to as the standard model, or the CSHKP model after its major contributors (Carmichael 1964; Sturrock 1966; Hirayama 1974; Kopp and Pneuman 1976, see Fig. 7a). In this paradigm and its updated versions (e.g., Forbes and Malherbe 1986; Shibata et al. 1995; Aulanier et al. 2012; Janvier et al. 2013), the key features are explained as follows. The magnetic flux rope becomes unstable and erupts into the higher atmosphere, entraining the overlying coronal field. The legs of the coronal field are drawn into a current sheet underneath the flux rope as inflows and reconnect with each other. The outflows from the reconnection region further boost the flux rope eruption. The post-reconnection field lines form a cusp structure, while the accelerated electrons from the reconnection site precipitate along the field lines and heat the chromosphere to produce flare ribbons.

The flux rope, if ejected successfully, expands and develops into the magnetic skeleton of a CME that travels through interplanetary space. This is well demonstrated by in-situ observations of magnetic fields at vantage points, e.g., in front of the Earth (Burlaga et al. 1981; Klein and Burlaga 1982; Marubashi 1986). Figure 7b shows a schematic illustration of the inferred topology. The helical nature of the magnetic field of the CMEs is strongly suggestive of their solar origins.

Regarding the onset of flux rope eruption and subsequent ejection of CMEs, various theories have extensively been proposed and investigated, such as flux emergence (Heyvaerts et al. 1977), breakout (Antiochos et al. 1999; DeVore and Antiochos 2008), tether-cutting (Moore et al. 2001), emerging-flux trigger (Chen and Shibata 2000), kink instability (Török and Kliem 2005; Fan and Gibson 2007), and torus instability (Kliem and Török 2006), along with a more recent concept of the double-arc instability (Ishiguro and Kusano 2017). In any case, there appears to be a consensus, at least, that the flare/CME occurrence is caused through the dynamical coupling between the unstable eruption of a flux rope (ideal MHD process) and magnetic reconnection of surrounding arcades (resistive MHD process).

It should be noted, however, that not all the stronger flares are accompanied by CMEs (e.g., Yashiro et al. 2006). The best example is the giant AR NOAA 12192 (Fig. 1). Throughout the disk passage, this AR produced numerous energetic flares including the six X-class ones, but surprisingly none of them were CME-eruptive. Sun et al. (2015) showed that in this AR, the decay index $n=-\partial \ln {B_{\mathrm{h}}}/\partial \ln {z}$, which measures the decreasing rate of the horizontal magnetic field $B_{\mathrm{h}}$ with height *z*, remains below the critical value $n_{\mathrm{c}}\approx 1.5$ for the torus instability until a large altitude and thus only failed eruptions took place (Inoue et al. 2016; Jiang et al. 2016a; Amari et al. 2018). The confinement of flux rope eruption by strong overlying field is also shown by the statistical studies on a number of ARs (Wang et al. 2017a; Vasantharaju et al. 2018; Jing et al. 2018). The same mechanism explains the observed result by Toriumi et al. (2017b) that the ratio of reconnected flux (in the flare ribbons) to the total AR flux is, on average, smaller for failed events than eruptive cases. DeRosa and Barnes (2018) showed that X-class flares located near coronal fields that are open to the heliosphere are eruptive at a higher rate than those lacking access to open fields.

The topics we have discussed above are only the most representative aspects of the flares and CMEs. In order to keep our primary focus on the formation and evolution of flare-productive ARs, however, we stop the discussion at this point and yield the rest to reviews by, e.g., Schrijver (2009), Fletcher et al. (2011) and Benz (2017) for observational overviews and Priest and Forbes (2002), Forbes et al. (2006), Chen (2011), Shibata and Magara (2011) and Janvier et al. (2015) for theoretical and modeling aspects.

### Categorizations of sunspots and flare productivity

**Fig. 8:**
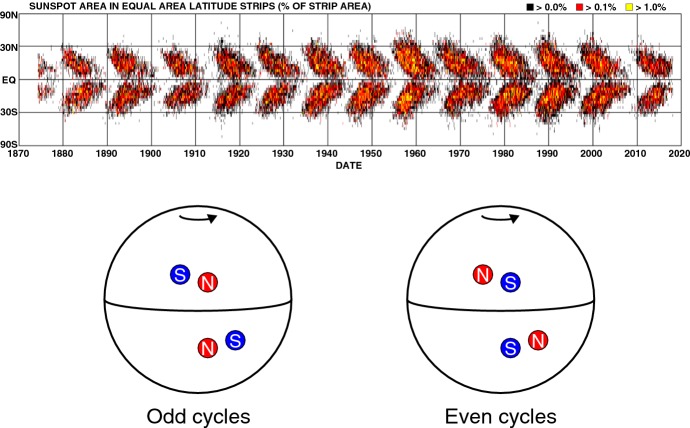
(Top) Sunspot butterfly diagram showing the total spot area as a function of time and latitude. Image courtesy of Hathaway. In each cycle, the latitudes of ARs shifts to the equator (Spörer’s law). (Bottom) Schematic diagram showing the polarity alignments. The preceding spots appear closer to the equator than the following spots (Joy’s rule). In each cycle, the preceding polarities on one hemisphere are the same and are opposite to those on the other hemisphere, and the order of the polarities reverses in the successive cycle (Hale–Nicholson rule). These are merely the overall trends and there exist many exceptional ARs

The number of sunspots varies with the 11 year solar activity cycle (Schwabe 1843; Hathaway 2015). Early in a cycle, the spots appear in higher latitudes up to $40^{\circ }$ and, throughout the cycle, the latitude gradually drifts lower to the equator (Spörer’s law: Carrington 1858). This behavior is illustrated by the Maunder butterfly diagram (Fig. 8 top). In each bipolar AR, the preceding spot tends to appear closer to the equator than the following spot (Joy’s rule: Hale et al. 1919). As the magnetic observation started in the beginning of twentieth century (Hale 1908), Hale’s polarity rule was discovered: for each cycle, the bipolar ARs are aligned in the east–west orientation with opposite preceding magnetic polarities on the opposite hemispheres. Soon, they also noticed that the polarities of the preceding spots alternate between successive cycles and these features are now altogether called Hale–Nicholson rule (Fig. 8 bottom: Hale and Nicholson 1925).

**Fig. 9:**
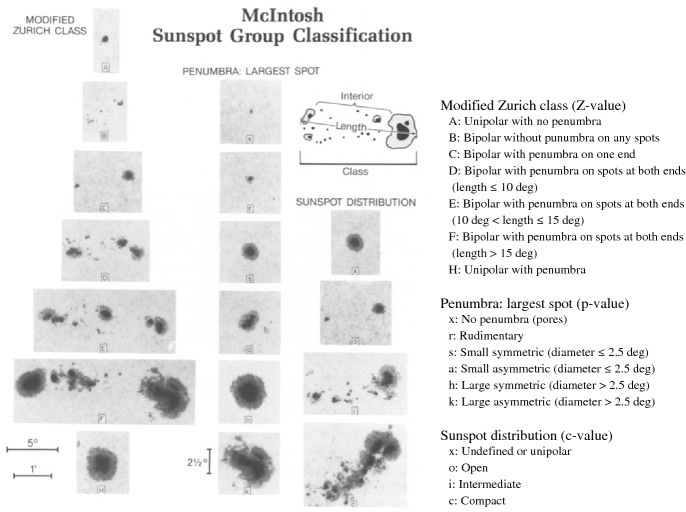
Example spot images for the three indices of McIntosh classification. Image reproduced by permission from McIntosh (1990), copyright by Kluwer

Along with such long-term characteristics, which impose strong constraints on dynamo models, the structure of each sunspot group is also recognized as an important factor (see reviews by Solanki 2003; Borrero and Ichimoto 2011). One method of categorizing the sunspots is the Zurich classification (Cortie 1901; Waldmeier 1938), which was further developed as the McIntosh classification (McIntosh 1990). The McIntosh classification uses three letters to describe the white-light properties of the spots, which are the size, penumbral type, and distribution (see Fig. 9). The combination of the three letters shows the *morphological complexity* of ARs and, according to Bornmann and Shaw (1994), the flare production rate increases along the diagonal line in the 3D parameter space from the simplest corner “A/B/Hxx” to the most complex end “Fkc”. Other studies show essentially a consistent result: morphologically complex ARs produce more flares (e.g., Atac 1987; Gallagher et al. 2002; Ternullo et al. 2006; Norquist 2011; Lee et al. 2012; McCloskey et al. 2016). The primary advantage of this method is that the spots are categorized simply from the white light observation and thus it requires no magnetic measurement.^4^

**Fig. 10:**
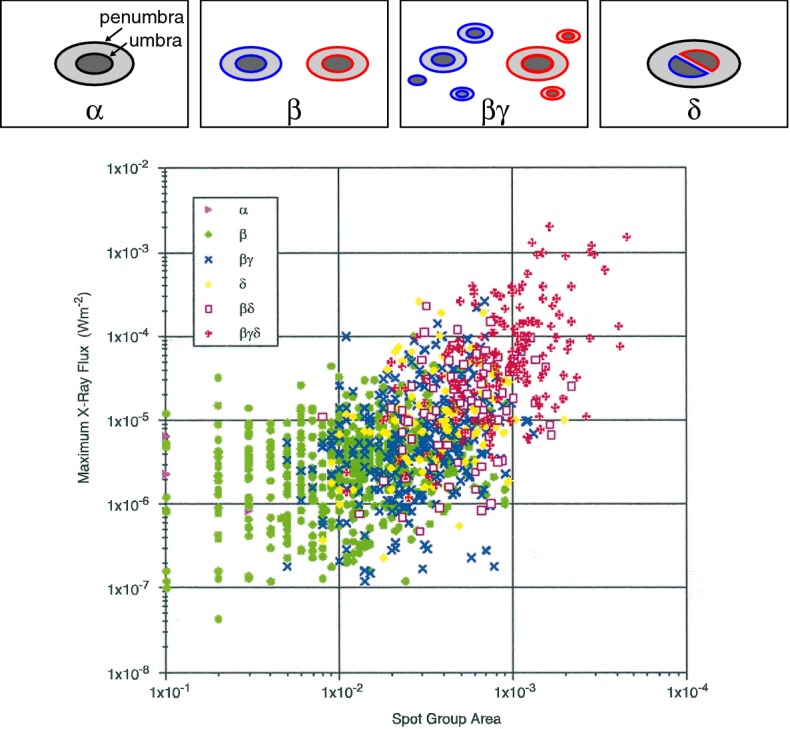
(Top) Sample diagrams of the Mount Wilson classification. (Bottom) Peak flare magnitudes as a function of maximum sunspot area. Image reproduced by permission from Sammis et al. (2000), copyright by AAS. Note that the tick marks of the horizontal axis should be corrected as, from left to right, $1\times 10^{-5}$, $1\times 10^{-4}$, $1\times 10^{-3}$, and $1\times 10^{-2}$ in the unit of the hemisphere, or equivalently, 10, 100, 1000, and 10,000 MSH

Another categorization method is the Mount Wilson classification, which refers to the *magnetic* structures of ARs. The original scheme of this method has the following three identifiers (Fig. 10 top: Hale et al. 1919; Hale and Nicholson 1938):

- $\alpha$, a unipolar spot group;
- $\beta$, a simple bipolar spot group of both positive and negative polarities; and
- $\gamma$, a complex spot group in which spots of both polarities are distributed so irregularly as to prevent classification as a $\beta$ group.

Often more than one identifier is appended to each AR to indicate even more complex structures, such as $\beta \gamma$, a bipolar spot group which is so complex that preceding or following spots are accompanied by minor polarities. It was shown that the flare productivity is related to this categorization. Giovanelli (1939) found that the probability of the flare eruption is proportional to the spot area and it increases with the spot complexity (in the order of $\alpha$, $\beta$, $\beta \gamma$, and $\gamma$). Consistent results were reported by Kleczek (1953), Bell and Glazer (1959) and Greatrix (1963).

Later, the $\delta$ group, a spot group in which umbrae of opposite polarities are separated by less than 2$^{\circ }$ and situated within the common penumbra, was added to the Mount Wilson classification by Künzel (1960, 1965). In this scheme, the most complex ARs are the spots appended with $\beta \gamma \delta$. Ever since Künzel (1960) showed that the $\delta$-spots are highly flare-productive, a number of statistical investigations have been carried out and showed consistent results (e.g. Mayfield and Lawrence 1985; Sammis et al. 2000; Tian et al. 2002; Ternullo et al. 2006; Guo et al. 2014; Toriumi et al. 2017b; Yang et al. 2017b). The bottom panel of Fig. 10 is a diagram of the peak GOES soft X-ray flux versus the maximum sunspot area for various ARs by Sammis et al. (2000). Here, one may easily find the clear positive correlation that the flare magnitude increases with the spot area. However, this diagram also shows that more complex regions produce stronger flares. For example, all $\ge$ X4-class flares occur in ARs of area greater than 1000 MSH and classified as the most complex $\beta \gamma \delta$. Other studies show the correlations and associations between the $\delta$-spots and the production of proton flares (here meaning that flares that emit energetic protons: Warwick 1966; Sakurai 1970), white-light flares (Neidig and Cliver 1983), $\gamma$-ray flares (Xu et al. 1991), and fast CMEs (Wang and Zhang 2008).

Yet another important finding is that the *inverted* or *anti-Hale* spot groups, i.e., the ARs violating Hale’s polarity rule, are flare productive (Smith and Howard 1968; Zirin 1970; Tang 1982). In most cases, polarities of ARs follow the Hale–Nicholson rule described earlier in this subsection and the spot groups violating this rule are very small in number (appearance rate being 3–9%; Richardson 1948; Wang and Sheeley 1989; Khlystova and Sokoloff 2009; Stenflo and Kosovichev 2012; McClintock et al. 2014). However, it is known that once this structure is created, an AR tends to produce strong flares. For example, Tian et al. (2002) selected the 25 most violent ARs in Cycles 22 and 23 based on five criteria: the largest spot area $>1000\, \mathrm{MSH}$; X-ray flare index (related to the sum of peak flare intensities) $>5.0$; 10.7 cm radio flux $>1000\, \mathrm{s.f.u.}$; proton flux ($>10\, \mathrm{MeV}$) $>400\, \mathrm{p.f.u.}$; and geomagnetic $A_{p}$ index $>50$. They found that most of them (68%) violate the Hale–Nicholson rule. Surveying 104 $\delta$-spots, Tian et al. (2005a) showed that about 34% violate the Hale’s rule but follow the hemispheric current helicity rule, which describes the dominance of negative (positive) current helicity in the northern (southern) hemisphere (e.g., Pevtsov et al. 1995, see also Sect. 3.3.3). Tian et al. (2005a) found that such ARs have a much stronger tendency to produce X-class flares.

**Fig. 11:**
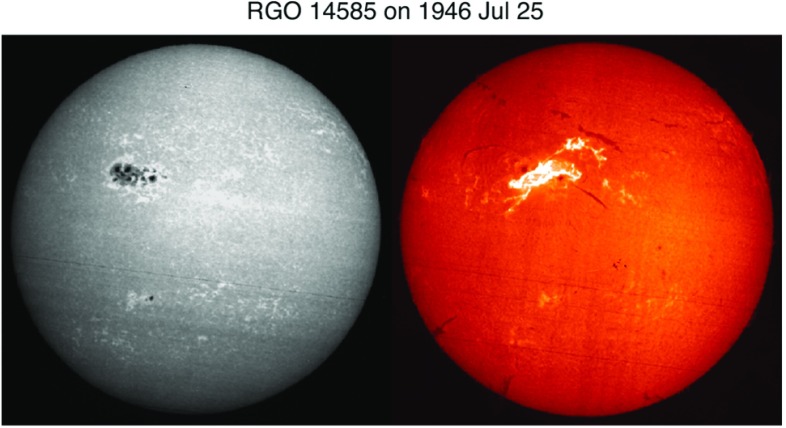
Great flare event in 1946 July 25 in RGO 14585, the fourth largest sunspot group since the late nineteenth century. A gorgeous two-ribbon flare breaks out in the huge, compact sunspot region. (Left) Sunspots observed in Ca ii K1v. (Right) Very large flare ribbons observed in H$\alpha$. Image reproduced by permission from Toriumi et al. (2017b), copyright by AAS and Paris Observatory

In this subsection, we reviewed several schemes of sunspot categorization and showed that ARs producing larger flares tend to have: a larger spot area; morphological and magnetic complexity, which is qualitatively indicated by McIntosh and Mount Wilson schemes; and anti-Hale alignment. However, for producing strong flares, probably it is not enough to satisfy just one of these conditions. For example, the largest-ever sunspot since the late nineteenth century, RGO (Royal Greenwich Observatory) 14886 on April 1947 (maximum spot area of 6132 MSH), is reported as flare quiet. The spot image shown in Fig. 3 of Aulanier et al. (2013) indicates that this region has a simple bipolar structure ($\beta$-spot). On the other hand, the fourth largest in history, RGO 14585 on July 1946 (4279 MSH) as in Fig. 11, produced great flares and geomagnetic storms with a ground-level enhancement (Ellison 1946; Forbush 1946; Dodson and Hedeman 1949). The spot image reveals that this region is strongly packed as if it is a $\delta$-spot and, judging from the Mount Wilson drawing, it is very likely true. Therefore, it is important to find if there exist critical conditions for the strong flares and, if so, what they are, by conducting observational and theoretical studies of any kinds to investigate the magnetic structure of flaring ARs and their evolution.

## Long-term and large-scale evolution: observational aspects

Observationally, the changes of magnetic fields that are associated with flares are often divided into two regimes: the long-term, gradual evolution of large-scale fields and the rapid changes associated with (i.e., in the time scales comparable to) the flare occurrence. In what follows (Sects. 3 and 4), we review the first topic, the long-term evolution, which is essentially related to the energy build-up process in the pre-flare state.

### Formation and development of $\delta$-spots

The role of long-term magnetic development in flare production was first recognized by Martres et al. (1968), who pointed out that the flares are often associated with evolving magnetic structures (Structure magnétique évolutive) of opposite polarities, in which one is growing and the other decreasing. Through accumulating a vast amount of observational data, observers gradually found certain regularities of flare-productive ARs. After 18 years of observations at Big Bear Solar Observatory (BBSO), Zirin and Liggett (1987) summarized and classified the formation of $\delta$-spots that produce great flares in three ways:

- Type 1: A complex of spots emerging all at once with different dipoles intertwined. This type is tightly packed with a large umbra and called “island $\delta$ sunspot”;
- Type 2: A single $\delta$-spot produced by emergence of satellite spots near large older spots; and
- Type 3: A $\delta$-configuration formed by collision between two separate but growing bipoles. The overall polarity layout is quadrupolar and the preceding spot of one bipole collides with the following spot of the other.

**Fig. 12:**
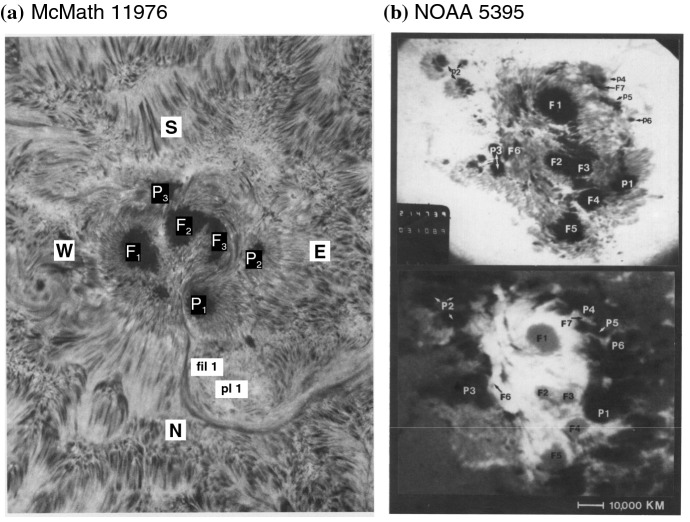
Examples of Type 1 $\delta$-spots. **a** AR McMath 11976 in August 1972. H$\alpha -0.5$ Å image on August 3. Umbrae numbered F1, F2, F3, P1, P2, and P3 all share a common penumbra. Image reproduced by permission from Zirin and Tanaka (1973), copyright by D. Reidel. **b** NOAA 5395 in March 1989. He D3 image and magnetogram on March 10. Image reproduced by permission from Wang et al. (1991), copyright by AAS

Figure 12 shows two typical examples of Type 1. The AR in Fig. 12a, McMath 11976, appeared in August 1972 and produced great flares (Zirin and Tanaka 1973). This region emerged as a tight complex of sunspots with inverted magnetic polarity (i.e., anti-Hale region). The negative spot P1 pushed into the positive spots (F1, F2, and F3) and caused steep magnetic gradient on the central PIL. The filament on the north (fil 1), which may be the extension of the central PIL, repeatedly erupted due to the continuous spot motion. Another example is NOAA 5395 in March 1989 (Fig. 12b: Wang et al. 1991). This region also had a closely packed structure of multiple spots and produced great flares including X4.5 (March 10) and X10 (March 12). This region is known to produce the geomagnetic storm that triggered the severe power outage in Quebec, Canada, on March 13 to 14 (e.g., Allen et al. 1989; Cliver and Dietrich 2013). The analysis shows that, at one edge of the large positive spot F1, negative polarities successively emerged and moved around the main spots, creating a clockwise spiraling penumbral fields around it (Wang et al. 1991; Tang and Wang 1993; Ishii et al. 1998). The series of strong flares occurred along the PIL surrounding the main positive spots. Similar island-$\delta$ sunspots are observed to show significant flaring activity, such as flares in McMath 13043 (July 1974), X20 event in NOAA 5629 (August 1989), X13 in NOAA 5747 (October 1989), and X12 in NOAA 6659 (June 1991) (Tanaka 1991; Tang and Wang 1993; Schmieder et al. 1994).

**Fig. 13:**
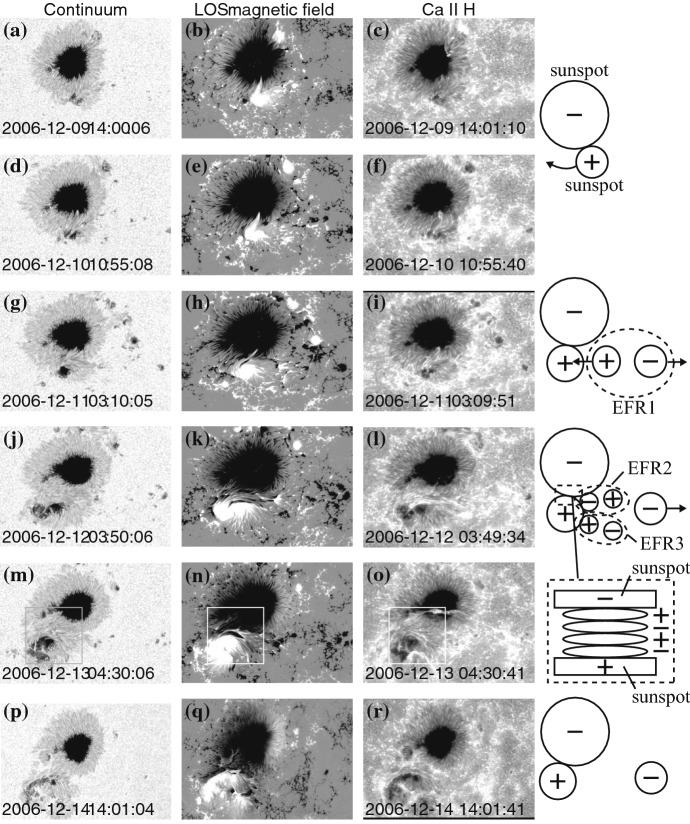
AR NOAA 10930 in December 2006 as the example of Type 2 $\delta$-spot obtained by Hinode/SOT. Daily evolution of continuum, magnetic fields, and Ca ii H is shown over the field of view of $128''\times 96''$. Images reproduced by permission from Kubo et al. (2007), copyright by ASJ

Type 2 events are the flare eruptions caused by the newly emerging satellite spots in the penumbra of an existing spot (Rust 1968) and Zirin and Liggett (1987) classified spot groups Mount Wilson 19469 and 20130 into this category (Patterson and Zirin 1981; Tang 1983). Figure 13 shows a clear example of this type, NOAA 10930 in December 2006 (Kubo et al. 2007). Within the southern penumbra of the main negative spot, a positive spot appears and drifts around to the east with showing a counter-clockwise rotation. As a result, an X3.4-class flare occurred on December 13 at the PIL between the main and the satellite spots (also refer to Fig. 6 and its corresponding movie).

**Fig. 14:**
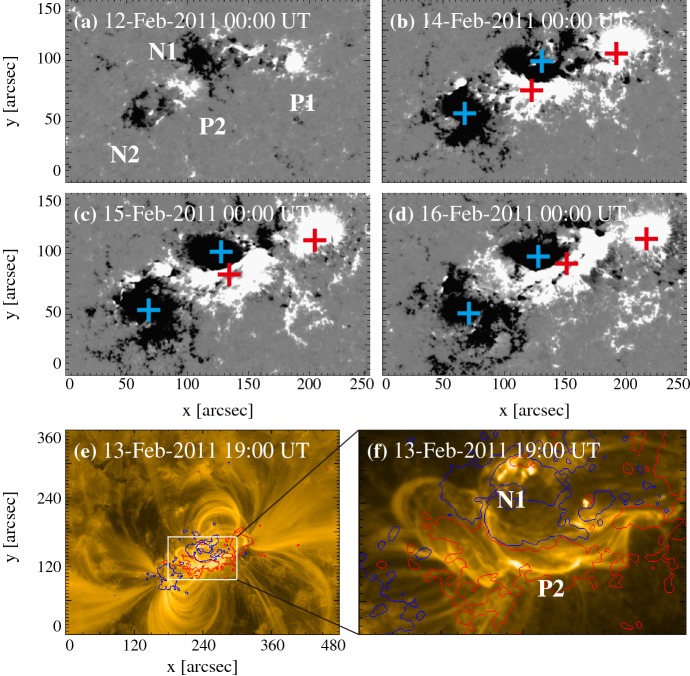
AR NOAA 11158 in February 2011 as the example of Type 3 $\delta$-spot. Image reproduced by permission from Toriumi et al. (2014b), copyright by Springer. Two emerging bipoles P1–N1 and P2–N2 collide against each other and produced a sheared PIL within a $\delta$-spot at the region center. The series of flares occur at the extended PIL between N1 and P2. Plus signs indicate the magnetic flux-weighted centroids of the four polarities. EUV images (panels **e** and **f**) show the field connectivity between N1 and P2

Figure 14 shows NOAA 11158 in February 2011, the typical case of Type 3 $\delta$-spot (Toriumi et al. 2014b). Because of the collision of two emerging bipoles P1–N1 and P2–N2, a highly sheared PIL with steep magnetic gradient is produced in the central $\delta$-spot (N1 and P2) and a series of flares including the X2.2-class event (February 15) occur. Similar structures are seen in a variety of ARs, such as NOAA 8562/8567, 6850, 7220/7222, 10314, and 10488 (van Driel-Gesztelyi et al. 2000; Kálmán 2001; Morita and McIntosh 2005; Poisson et al. 2013; Liu and Zhang 2006).

**Fig. 15:**
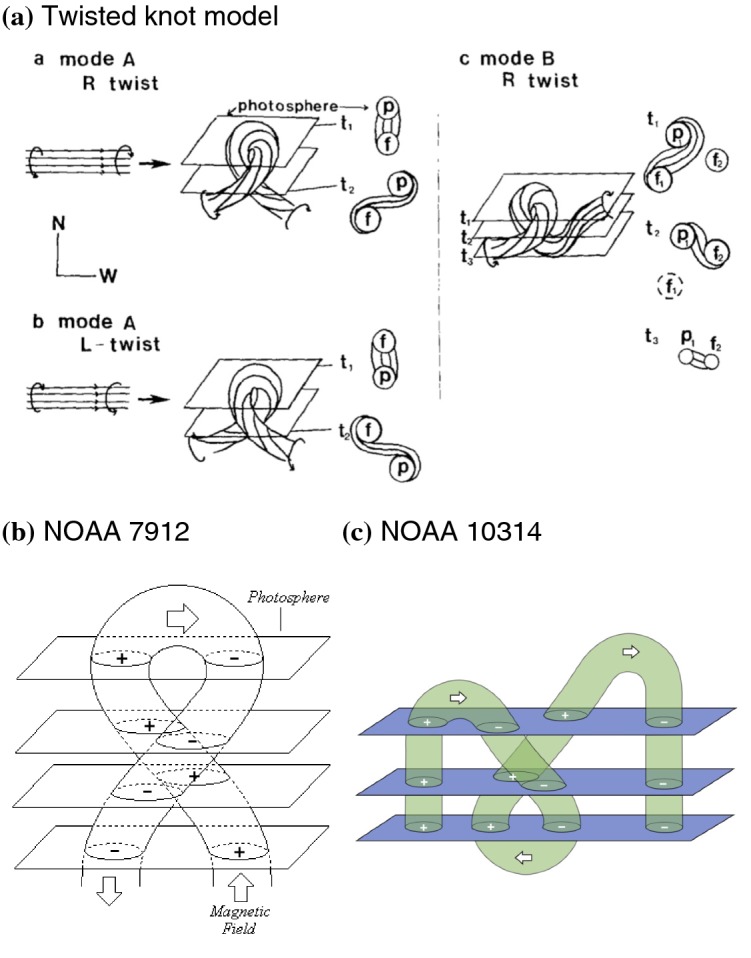
**a** Evolution patterns responsible for great flare occurrence and their explanations by an emerging twisted knot model. Mode A is a shearing process with spot growth and Mode B is an unshearing process with spot disappearance. Intersections represent the photosphere at times $t_{1}$, $t_{2}$ and $t_{3}$. Image reproduced by permission from Tanaka (1991), copyright by Kluwer. **b**, **c** Inferred 3D topologies for NOAA 7912 and 10314. Images reproduced by permission from López Fuentes et al. (2000) and Poisson et al. (2013), copyrights by AAS and COSPAR, respectively

**Fig. 16:**
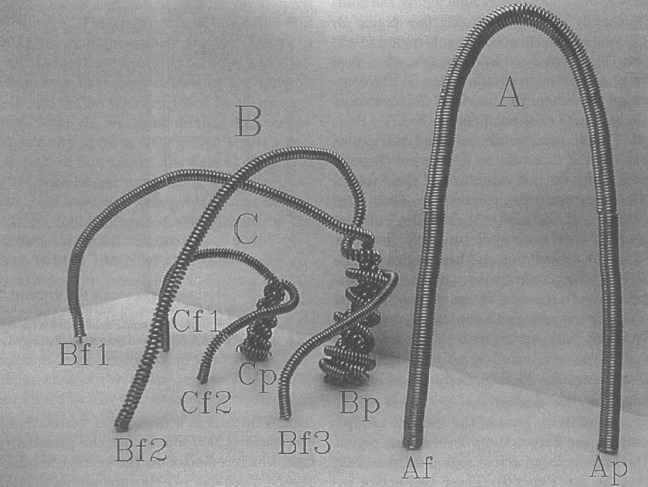
3D model made of flexible wires for explaining the evolution of NOAA 4021. Image reproduced by permission from Ishii et al. (2000), copyright by ASJ

How are these complex structures formed? Zirin and Liggett (1987) mentioned that “because Types 1 and 2 erupt in the same place, and Type 3 requires large dipoles that are not close by mere accident, the $\delta$ configuration must be the product of a subsurface phenomenon.” However, we cannot directly observe below the surface.

One way to reconstruct the 3D topology of emerging magnetic fields is to study it using sequential images (e.g., white light and magnetograms). For example, Tanaka (1991) studied the evolution of flare-active Type 1 $\delta$-spots McMath 13043 and 11976 and explained the observed proper motions, the non-Hale spots turning to obey it, by the emergence of knotted twisted flux tubes (twisted knot model: Fig. 15a). This scenario was supported by many successive researchers (e.g., Fig. 15b) and it was suggested that the deformation of emerging $\varOmega$-loops is due to the helical kink instability (e.g., Lites et al. 1995; Leka et al. 1996; López Fuentes et al. 2000, 2003; Holder et al. 2004; Tian et al. 2005a, b; Nandy 2006; Takizawa and Kitai 2015) (see Sect. 4.1.1 for theoretical investigations on the kink instability and “Appendix” for the story of the original advocates of this instability as the formation mechanism of the $\delta$-spots). Poisson et al. (2013) explained the formation of Type 3 $\delta$-spot NOAA 10314 as the ascent of a single large $\varOmega$-loop whose top is curled downward and has a U-loop below the photosphere (Fig. 15c; see also Pevtsov and Longcope 1998; van Driel-Gesztelyi et al. 2000; Takizawa and Kitai 2015). Ishii et al. (2000) and Kurokawa et al. (2002) even used flexible wires to manually model the inferred 3D configurations (Fig. 16). From vertically stacked sequential magnetograms, Chintzoglou and Zhang (2013) inferred the subsurface topology of NOAA 11158 (Fig. 14). These observations consistently show that the emerging flux tubes of $\delta$-spots do not have a simple $\varOmega$-shape but are deformed within the convection zone, prior to emergence.

**Fig. 17:**
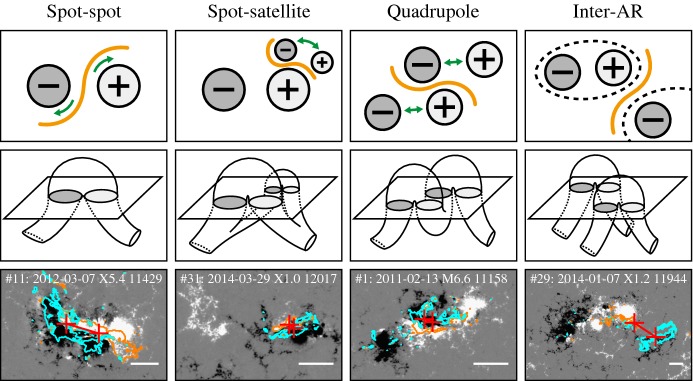
Classification of flaring ARs. Image reproduced by permission from Toriumi et al. (2017b), copyright by AAS. (Top) Polarity distributions. Magnetic elements (spots) are indicated by circles with plus and minus signs. The PIL or filament involved in the flare is shown with an orange line, while proper motions of the polarities are indicated with green arrows. (Middle) Possible 3D structures of magnetic fields. Solar surface is indicated with a horizontal slice. (Bottom) Sample events. Gray scale shows magnetogram, overlayed by temporally stacked flare ribbons (orange and turquoise). Red plus signs show the area-weighted centroids of the ribbons. The white lines at the bottom right indicate the length of $50''$

Toriumi et al. (2017b) surveyed all $\ge$ M5-class flares within 45$^{\circ }$ from disk center for six years from May 2010 and classified the host ARs into four groups depending on their developments (Fig. 17): (1) Spot-spot, a complex, compact $\delta$-spot, in which a large long, sheared PIL extends across the whole AR (equivalent to Type 1 $\delta$-spot); (2) Spot-satellite, in which a newly emerging bipole appears in the vicinity of a preexisting main spot (i.e., Type 2); and (3) Quadrupole, a $\delta$-spot is created by the collision of two bipoles (i.e., Type 3). However, they also noticed that even X-class events do not require $\delta$-spots or strong-gradient PILs. Instead, some events occur between two independent ARs, situations called (4) Inter-AR events (Dodson and Hedeman 1970). For example, the X1.2 event on 2014 January 7 occurred between NOAA 11944 and 11943 (Möstl et al. 2015; Wang et al. 2015). Figure 17 also provides possible 3D topologies, which were later modeled by numerical simulations (see Sect. 4.1.5).

Through the analysis of Mount Wilson classifications from 1992 to 2015, Jaeggli and Norton (2016) discussed the possible production mechanism of complex ARs. They found that while the fractions of $\alpha$- and $\beta$-spots remain constant over cycles (about 20% and 80%, respectively), that of complex ARs appended with $\gamma$ and/or $\delta$ increases drastically from 10% at solar minimum to more than 30% at maximum. According to the authors, this may indicate that complex ARs are produced by the collision of simpler ARs around the surface layer through the higher rate of flux emergence during solar maximum. This idea may be related to the successive emergence model (Kurokawa 1987) and perhaps to the concepts of “complexes of activities” and “sunspot nests” (Bumba and Howard 1965; Gaizauskas et al. 1983; Castenmiller et al. 1986; Gaizauskas et al. 1994).

### Photospheric features

#### Strong-field, strong-gradient, highly-sheared PILs and magnetic channels

Because flares are the release of magnetic energy via magnetic reconnection, it is natural that these events are observed around the PILs, where the electric currents are strongly enhanced (see, e.g., Fig. 6). Since this fact was first pointed out by Severny (1958), the importance of the PILs in the flare occurrence has been repeatedly emphasized (e.g., Zirin and Tanaka 1973; Hagyard et al. 1984; Wang et al. 1996; Schrijver 2007). The photospheric characteristics of the flaring PILs are summarized as follows.

**Fig. 18:**
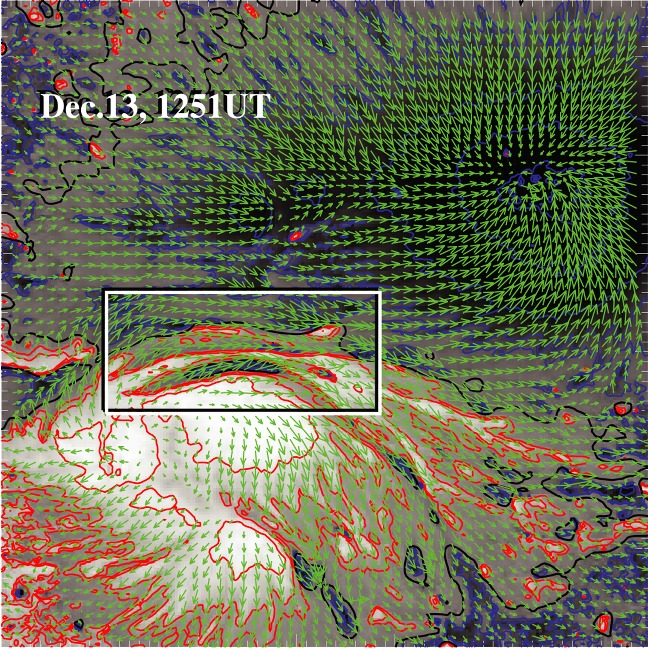
Hinode/SOT/SP vector magnetogram of AR NOAA 10930, which produced the X3.4-class flare (see Figs. 6, 13). The image shows the LOS magnetic fields (gray scale), transverse fields (green arrows), positive and negative polarities (red and blue contours), and the PILs (black contours). The FOV is $66''\times 66''$. The area around the sheared PIL is marked with a rectangular box. Image reproduced by permission from Wang et al. (2008), copyright by AAS

- Strong field: Both the vertical fields surrounding the PIL and the transverse fields along the PIL are very strong. Tanaka (1991) and Zirin and Wang (1993b) reported on the detection of strong transverse fields of up to 4300 G (see also Jaeggli 2016; Wang et al. 2018a). Livingston et al. (2006) also pointed out that part of the exceptionally strong fields they found are likely related to the transverse fields in light bridges of $\delta$-spots (i.e., PILs). Okamoto and Sakurai (2018) noticed the fields as high as 6250 G in a PIL, which is probably the highest value ever measured on the Sun including the sunspot umbrae.
- Strong gradient: The horizontal gradient of the vertical field across the PIL is steep, indicating that positive and negative polarities are tightly pressed against each other (Moreton and Severny 1968; Wang et al. 1991, 1994b). The gradient is sometimes up to several $100\, \mathrm{G\ Mm}^{-1}$ (Wang and Li 1998; Jing et al. 2006; Song et al. 2009).
- Strong shear: The transverse field is directed almost parallel to the PIL. The shear angle is often measured in the frame where $0^{\circ }$ is the azimuth of a potential field (Hagyard et al. 1984; Lu et al. 1993), and large shears of $80^{\circ }$–$90^{\circ }$ are observed at flaring PILs (Hagyard et al. 1990; Hagyard 1990). Figure 18 clearly shows that the transverse fields at the PIL of NOAA 10930 are along the direction of the PIL (marked by the box).

The strong-field, strong-gradient, highly-sheared PILs may be the direct manifestation of non-potentiality of magnetic fields and, therefore, these features are often used for the prediction of flares and CMEs. Falconer et al. (2002, 2006) measured the lengths of PILs of, e.g., strong transverse field ($>150\, \mathrm{G}$), large shear angle ($>45^{\circ }$), and steep gradient ($> 50\, \mathrm{G\ Mm}^{-1}$) and demonstrated that these parameters predict the occurrence of CMEs. Schrijver (2007) evaluated the total unsigned flux near the strong-gradient PILs and showed that it gives the upper limit of possible GOES flare class.

**Fig. 19:**
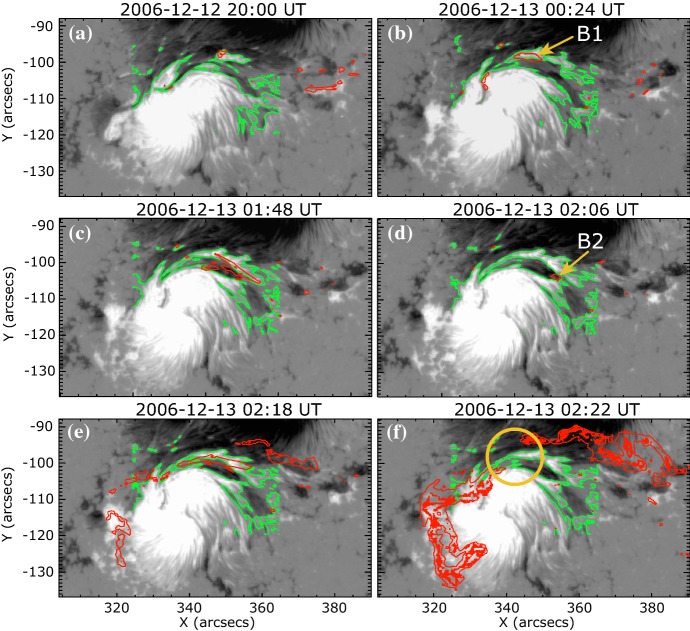
Temporal evolution of the X3.4-class flare in AR NOAA 10930. Background shows the LOS magnetogram, over which the PILs are plotted with green lines. The red contours show the Ca ii H line enhancement. The pre-flare brightening (such as B1) continuously occurs around the central PIL (yellow circle). The flare ribbons originate and expand from this region (see, e.g., progenitor brightening of B2). Image reproduced by permission from Bamba et al. (2013), copyright by AAS

Another important feature of the flaring PILs is the “magnetic channel”, which is an alternating pattern of elongated positive and negative polarities (Zirin and Wang 1993a; Wang et al. 2002a). Figure 18 displays the magnetic channel in NOAA 10930 (see PIL marked by the box). Wang et al. (2008) and Lim et al. (2010) showed that high resolution with high polarimetric accuracy is needed to adequately resolve such small-scale structures (width $\lesssim 1''$). Figure 19 clearly shows that the pre-flare brightening continues around this structure and the flare ribbons originate from here (see also the movie of Fig. 6). From these observations, Bamba et al. (2013) suggested that such fine-scale magnetic structures galvanize the whole system into producing flare eruptions (Toriumi et al. 2013a; Bamba et al. 2017; Bamba and Kusano 2018).

**Fig. 20:**
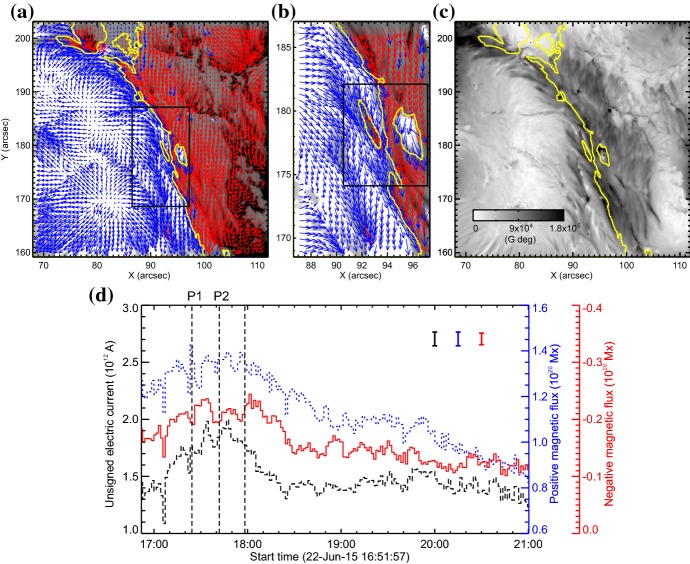
BBSO/GST observation of magnetic field in AR NOAA 12371 before the M6.5-class flare at 18:23 UT on 2015 June 22. **a**, **b** GST/NIRIS photospheric vertical magnetic field (scaled between $\pm \, 1500\, \mathrm{G}$) at 17:35 UT, superimposed with arrows representing horizontal magnetic field vectors. The box in **a** denotes the FOV of **b**, in which the magnetic channel structure can be obviously observed. **c** Distribution of magnetic shear in terms of a product of the field strength and shear angle. The overplotted yellow contour in **a**–**c** is the PIL. **d** Temporal evolution of total positive (blue dotted line) and negative (red solid line) magnetic fluxes and the unsigned electric current (black dashed line), calculated over the magnetic channel region enclosed by the box in **b**. The first two vertical dashed lines indicate the times of two flare precursor episodes. Image reproduced by permission from Wang et al. (2017b), copyright by Macmillan

The significance of the sheared PIL, magnetic channel, and small-scale trigger was also verified by a super high-resolution observation by BBSO/GST. Figure 20 shows the GST/NIRIS magnetogram of AR NOAA 12371. Here, Wang et al. (2017b) found that the field is highly sheared with respect to the PIL, especially in the precursor brightening region [panels (a) and (b)]. This signifies a high degree of non-potentiality, as reflected by the concentration of magnetic shear along the PIL [panel (c)]. In the region around the initial precursor brightening enclosed by the box in panel (b), they observed a miniature version of a magnetic channel with a scale of only 3000 km, which can also be recognized as the flare-triggering field. Importantly, the evolutions of both polarities within the channel are temporally associated with the occurrence of precursor episodes [panel (d)].

#### Flow fields and spot rotations

Given the high-$\beta$ condition in the photosphere, it was speculated that such flaring PILs are generated by the sheared, converging flow fields around it. In fact, Harvey and Harvey (1976) observed strong shear flows along the flaring PILs and associated these flows with the occurrence of flares (Meunier and Kosovichev 2003; Yang et al. 2004; Deng et al. 2006; Shimizu et al. 2014). Also, Keil et al. (1994) showed that the flare kernels correspond to the locations of convergence in the horizontal flows. The converging flow and the sustained cancellation of positive and negative polarities on the two sides of the PIL are thought to be the key process in building up a magnetic flux rope (van Ballegooijen and Martens 1989, see also Sect. 3.3.1 of this article for detailed discussion).

The large-scale spot motions drive the flow fields around the PILs and, because of the frozen-in state of the field, the magnetic structures are reconfigured. For instance, Krall et al. (1982) revealed that the shear flow in the PIL is in association with rapid spot motions, which enhances the magnetic shear at the PIL and leads to the series of flares. Wang (1994) observed that magnetic shear development is intrinsically related to the newly emerging flux.

**Fig. 21:**
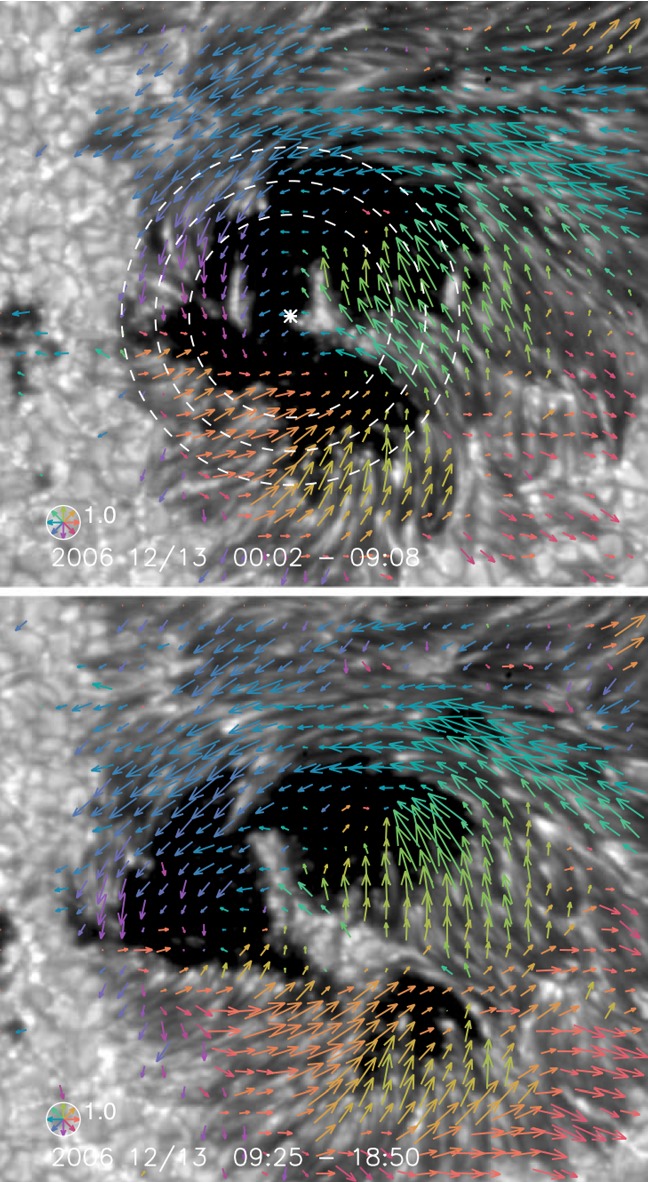
Velocity field of the southern sunspot in AR NOAA 10930 over the FOV of $42''\times 38''$. The radius of the circle in the lower-left corner corresponds to a speed of $0.22\, \mathrm{km\ s}^{-1}$, and the color of an arrow corresponds to its direction. Image reproduced by permission from Min and Chae (2009), copyright by Springer

Strong spot rotations (both the spot rotating around its center and the spot rotating around its counterpart in the same AR) are also often observed in the pre-flare state. Figure 21 is a clear example of rotating sunspots in AR NOAA 10930 (Min and Chae 2009). This figure highlights that the southern spot rotates in the counter-clockwise direction before the X3.4-class flare occurs. Brown et al. (2003) analyzed rotating sunspots in seven ARs and found that the spots rotate around their umbral centers up to 200$^{\circ }$ in 3–5 days. The coronal loops are twisted as the spot rotates, and six of them showed flares and/or CMEs (Régnier and Canfield 2006; Zhang et al. 2007, 2008; Vemareddy et al. 2012; Ruan et al. 2014; Vemareddy et al. 2016). Brown et al. (2003) considered that the spot rotation is caused by the flux tube emergence (see Sect. 4.1 for the discussion). The observed association of spot rotations and eruptions is consistent with the theoretical suggestion by Stenflo (1969) and Barnes and Sturrock (1972) that such spot rotations accumulate flare energy in the atmosphere. Yan et al. (2008) surveyed 186 rotating sunspots in 153 ARs and statistically investigated the relationship between the spot rotation and the flare productivity. They found that ARs with sunspots of rotation direction opposite to the global differential rotation are in favor of producing M- and X-class flares.

These flow fields and spot motions strongly suggest the possibility that the flaring ARs, if not all, are produced by the emergence of magnetic flux with a strong twist. Through these processes, the magnetic flux transports the energy and magnetic helicity (Sect. 3.2.3) from the subsurface layer to the atmosphere.

#### Injection of magnetic helicity

Magnetic helicity is a measure of magnetic structures such as twists, kinks, and internal linkage (Elsasser 1956) and is a useful tool to quantify and characterize the complexity of flaring ARs. The magnetic helicity of the magnetic field ${\mathbf {B}}$ fully contained in a volume *V* (i.e., the normal component $B_{n}$ vanishes at any point of the surface *S*) is defined as

4 $$\begin{aligned} H=\int _{V} {{\mathbf {A}}}\cdot {{\mathbf {B}}}\, dV, \end{aligned}$$

where ${{\mathbf {A}}}$ is the vector potential of ${{\mathbf {B}}}$, i.e., ${{\mathbf {B}}}=\nabla \times {{\mathbf {A}}}$. *H* is invariant to gauge transformations and, in ideal MHD, *H* is a conserved quantity. Even under resistive MHD where magnetic reconnection can occur, it is shown that dissipation of *H* is much slower than dissipation of magnetic energy (Berger 1984). However, in many practical situations, the field lines cross the surface of the volume of interest *S* (e.g., the photosphere) and thus it is convenient to use the relative magnetic helicity (Berger and Field 1984; Finn and Antonsen Jr 1985):

5 $$\begin{aligned} H_{\mathrm{R}}=\int _{V} ({{\mathbf {A}}}+{{\mathbf {A}}_{0}})\cdot ({{\mathbf {B}}}-{{\mathbf {B}}_{0}})\, dV, \end{aligned}$$

where ${{\mathbf {A}}}_{0}$ and ${{\mathbf {B}}}_{0}$ are the reference vector potential and magnetic field, respectively (${{\mathbf {B}}}_{0}$ has the same $B_{n}$ distribution on *S*). $H_{\mathrm{R}}$ is also a gauge-invariant quantity, and often the potential field ${{\mathbf {B}}}_{\mathrm{p}}$ $(=\nabla \times {{\mathbf {A}}}_{\mathrm{p}})$ is chosen as the reference field:

6 $$\begin{aligned} H_{\mathrm{R}}=\int _{V} ({{\mathbf {A}}}+{{\mathbf {A}}_{\mathrm{p}}})\cdot ({{\mathbf {B}}}-{{\mathbf {B}}_{\mathrm{p}}})\, dV. \end{aligned}$$

One way to calculate the relative helicity in the coronal volume is to rely on 3D magnetic extrapolations as it is not yet possible to fully measure the magnetic fields in the atmosphere (Sect. 4.3.1). Alternatively, it is also possible to monitor the helicity flux (helicity injection rate) through the photosphere over the AR,^5^

7 $$\begin{aligned} \frac{dH_{\mathrm{R}}}{dt} = 2\int \left[ ({{\mathbf {A}}}_{\mathrm{p}}\cdot {{\mathbf {B}}})v_{n} -({{\mathbf {A}}}_{\mathrm{p}}\cdot {{\mathbf {v}}})B_{n} \right] \, dS, \end{aligned}$$

where ${{\mathbf {v}}}$ is the velocity of the plasma and $v_{n}$ is the component normal to the surface. This parameter has been used more commonly to investigate the accumulation of helicity during the course of AR evolution (Chae 2001; Chae et al. 2001; Green et al. 2002; Nindos et al. 2003; Chae et al. 2004). Note that in the last equation, the first and second terms in the bracket are called the “emergence term” and “shear term,” respectively.

**Fig. 22:**
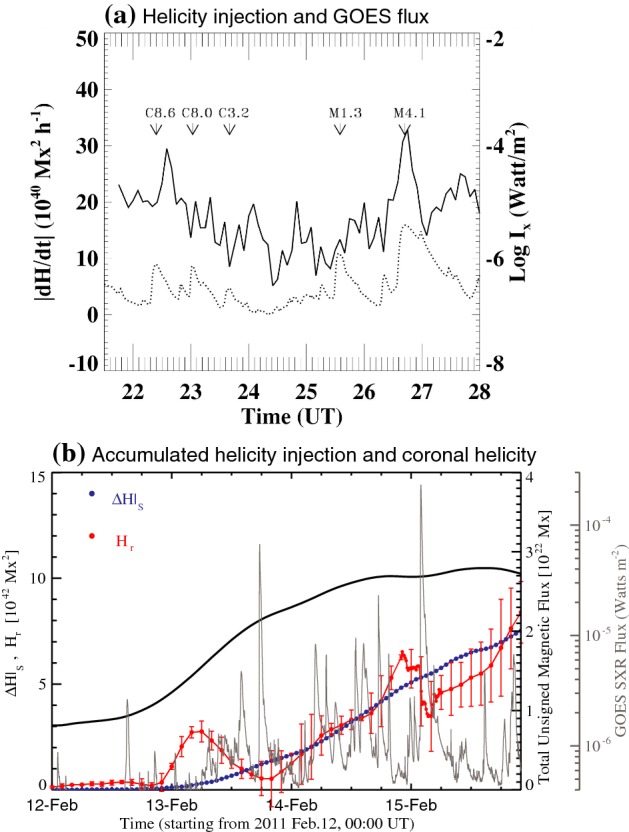
**a** Temporal evolution of the magnetic helicity injection rate (solid line) and the GOES soft X-ray flux (dotted line) over 6.5 h. The arrows indicate the X-ray intensity peak of homologous flares in AR NOAA 8100. Image reproduced by permission from Moon et al. (2002a), copyright by AAS. **b** Temporal variation of magnetic helicity. Plotted are the coronal helicity derived from the NLFFF extrapolation $H_{\mathrm{r}}$ (red dots), the accumulated amount of helicity injection through the photosphere $\varDelta H|_{S}$ (blue dots), total unsigned magnetic flux (black) and GOES flux (gray). The uncertainty in $H_{\mathrm{r}}$ is indicated by the error bars. The uncertainty in $\varDelta H|_{S}$ is generally 0.5% that is too small to be plotted. Image reproduced by permission from Jing et al. (2012), copyright by AAS

Many observational studies have shown the temporal relationship between the helicity injection and the occurrence of flares and CMEs (Moon et al. 2002a, b; Chae et al. 2004; Magara and Tsuneta 2008; Park et al. 2008, 2012). For instance, Moon et al. (2002a, b) revealed that the significant amount of helicity was impulsively injected around the peak time of X-ray flux of the flare events they studied, especially for the strong ones (Fig. 22a). The authors attributed the observed impulsive helicity injection to the horizontal velocity anomalies near the PIL. However, because the location of helicity injection is near the flaring site (e.g., H$\alpha$ flare ribbons), the possibility can not be ruled out that the observation is affected by an artifact of the magnetogram (SOHO/MDI) due to emission caused by particle precipitation that changes the spectral line’s shape.

From long-term monitoring, Park et al. (2008, 2012) found that the helicity first increases monotonically and then remains almost constant just before the flares. Some events show the sign of injected helicity reverses and, in such cases, the flares are more energetic and impulsive and the accompanying CMEs are faster and more recurring. Park et al. (2010a) and Jing et al. (2012) compared the accumulated helicity injection measured by integrating Eq. (7) over time and the coronal helicity derived from the NLFFF extrapolation (Sect. 4.3.1) and found close correlations between the two parameters (see Fig. 22b).

From the viewpoint of helicity budget, the CME works as a carrier of helicity that is taken away from a flaring AR and leads the magnetic system of the AR to lower energy states (see illustration in Fig. 7b: Rust 1994; Démoulin et al. 2002; Green et al. 2002). However, accumulated helicity may also be reduced by annihilation of two magnetic systems of opposite helicity sign (through magnetic reconnection). Several observations show that magnetic systems with oppositely singed helicity commonly exist in a given AR and the interaction of these systems play a key role in driving flares and CMEs (Kusano et al. 2002; Wang et al. 2004c; Chandra et al. 2010; Romano et al. 2011; Zuccarello et al. 2011). This scenario is further supported by MHD simulations by Kusano et al. (2004, 2012), in which the emergence of reversed shear near the PIL triggers the eruption.

**Fig. 23:**
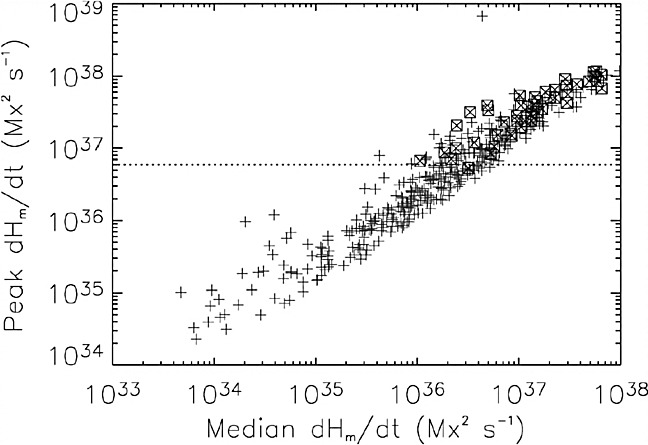
Peak helicity injection rate during the observing interval versus the median helicity flux over the interval. Non-X-flaring reference regions (345) are plotted as plus signs and X-flare regions (48) as boxed crosses. The necessary condition for the production of an X-flare is a peak helicity flux $>6\times 10^{36}\, \mathrm{Mx}^{2}\, \mathrm{s}^{-1}$. Image reproduced by permission from LaBonte et al. (2007), copyright by AAS

Statistical investigations on a number of ARs clearly demonstrate the tendency that flare-productive ARs have a significantly higher amount of helicity than flare-quiet ARs (Nindos and Andrews 2004; Park et al. 2010b). LaBonte et al. (2007) compared 48 X-flare-producing ARs and 345 non-X-flaring regions and derived an empirical threshold for the occurrence of an X-class flare that the peak helicity flux exceeds a magnitude of $6\times 10^{36}\, \mathrm{Mx}^{2}\, \mathrm{s}^{-1}$ (see Fig. 23). Tziotziou et al. (2012, 2014) found a consistent monotonic scaling between the relative helicity and the free magnetic energy for both observational data sets and MHD simulations (Moraitis et al. 2014). However, it should be noted that these results do not take into account the area of ARs. Because the magnetic helicity in a flux system scales as the square of that system’s magnetic flux, we can compare, by normalizing the magnetic helicity by the flux squared, how much the magnetic configuration is stressed in ARs of the same size (Démoulin and Pariat 2009).

As mentioned above, flaring ARs exhibit a fairly complicated distribution of both positive and negative signs of magnetic helicity. The helicity flux distribution can be measured by computing and mapping the density of helicity flux in Eq. (7): $G_{A}=2[({{\mathbf {A}}}_{\mathrm{p}}\cdot {{\mathbf {B}}})v_{n}-({{\mathbf {A}}}_{\mathrm{p}}\cdot {{\mathbf {v}}})B_{n}]$, or simply $G_{A}=-2({{\mathbf {A}}}_{\mathrm{p}}\cdot {{\mathbf {v}}})B_{n}$. However, Pariat et al. (2005) showed that $G_{A}$ is not a proper helicity flux density as $G_{A}$ can be non zero ($G_{A}$ map can show variation) even with simple translational motions that do not inject any magnetic helicity. Then, they proposed an alternative proxy of the helicity flux density, $G_{\varPhi }$, which takes into account the magnetic field connectivity and thus requires 3D magnetic extrapolations. Dalmasse et al. (2013, 2014) developed a method to compute $G_{\varPhi }$ and applied it to observational data of the complex flaring AR NOAA 11158 (Fig. 14), showing that this proxy reliably and accurately maps the distribution of photospheric helicity injection.

#### Magnetic tongues and importance of structural complexity

**Fig. 24:**
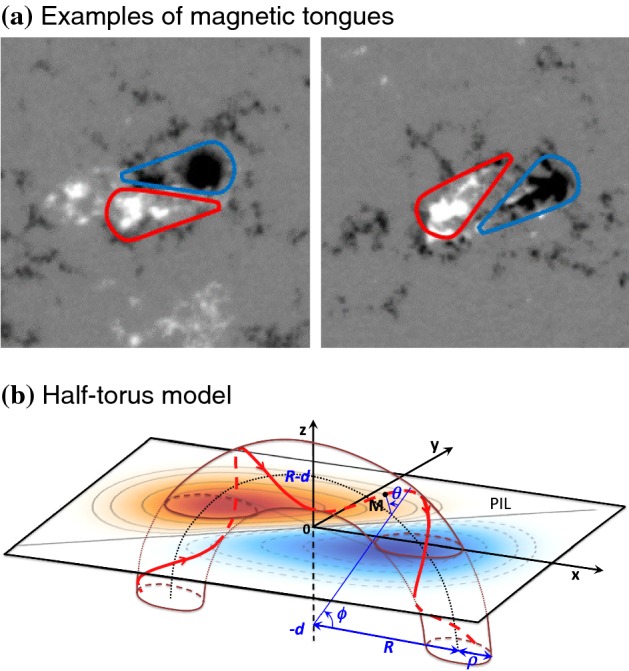
**a** Sample images of magnetic tongues resembling the yin-yang pattern. The left panel shows the tongue with negative helicity (left-handed twist), while the right panel is for positive helicity (right-handed twist). Image reproduced by permission from Takizawa and Kitai (2015), copyright by Springer. **b** Model of a twisted flux tube with a half-torus shape. The magnetic tongue (red-blue), separated by the PIL (straight line), is explained by the emergence of a twisted flux tube. In this case, the magnetic tongue has positive helicity due to the emergence of a flux tube with right-handed twist. Image reproduced by permission from Poisson et al. (2016), copyright by Springer

In vertical (or LOS) magnetograms, the newly emerging regions, especially of AR scales, display “magnetic tongue” structures, the extended magnetic polarities at both sides of the PIL (Fig. 24a), first mentioned by López Fuentes et al. (2000). The magnetic tongues that resemble the yin-yang pattern are thought to be the vertical projection of the poloidal component of the twisted emerging magnetic flux tube (Fig. 24b), and thus, the layout of tongues and the direction of PILs are used as proxies of magnetic helicity sign of emerging fields (Sect. 3.2.3: Luoni et al. 2011; Takizawa and Kitai 2015; Poisson et al. 2015, 2016). Multiple observational studies showed that such yin-yang tongues are seen in flaring ARs, along with other observational characteristics including sigmoids, sheared coronal loops, and J-shaped flare ribbons (Li et al. 2007; Green et al. 2007; Canou et al. 2009; Chandra et al. 2009; Mandrini et al. 2014). This may indicate that the flaring ARs tend to possess substantial magnetic helicity.

One of the important conclusions from the series of statistical investigations in Sect. 2.3 was that magnetic fields of flare-productive ARs exhibit higher degrees of *complexity*. While classical sunspot categorizations (e.g., McIntosh and Mount Wilson schemes) simply provide qualitative indices of the ARs’ complexity, one well-studied quantitative measure of the complexity is the fractal dimension, an indication of self-similarity of structures (Mandelbrot 1983). From the fractal dimension analysis using full-disk magnetograms over 7.5 years, McAteer et al. (2005) found that the flare productivity, in terms of both GOES magnitude and frequency, has a good correlation with fractal dimension. They showed a threshold fractal dimension of 1.2 and 1.25 as a necessary requirement for an AR to produce M- and X-class flares, respectively, within next 24 h period. Interestingly, McAteer et al. (2005) also found that the frequency distributions of the fractal dimension for different Mount Wilson classes ($\alpha$, $\beta$, $\beta \gamma$, $\beta \gamma \delta$) are similar to each other with a mean fractal dimension of 1.32. Perhaps this result indicates that, for the production of strong flares, the complexity of mid-to-small scales (smaller than the whole AR: detected by the fractal dimension analysis) has to exist along with the large-scale complexity (AR size: characterized by the Mount Wilson class).

**Fig. 25:**
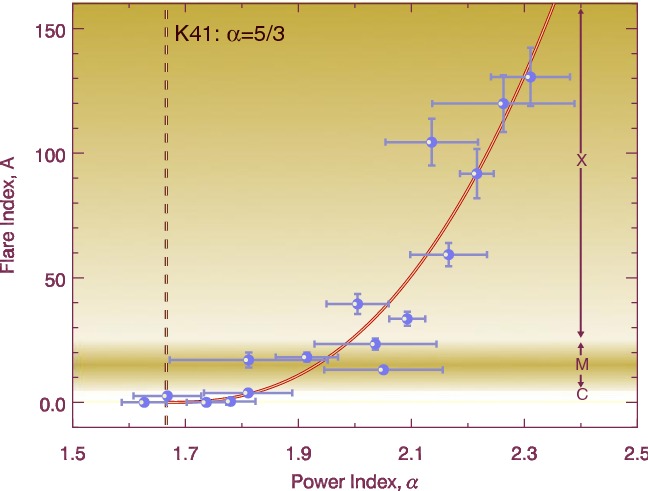
Power-law index $\alpha$ for 16 ARs of different flare index (denoted as *A* in this panel). The dashed vertical line indicates $\alpha =5/3$ for the Kolmogorov’s turbulence theory. The positive relationship between the flare productivity and the power-law index is clearly illustrated. Image reproduced by permission from Abramenko (2005), copyright by AAS

Importance of structural complexity in the flare production is also demonstrated by plotting the power spectra of magnetograms. Abramenko (2005) calculated the power-law index $\alpha$ of the magnetic power spectrum $E(k)\sim k^{-\alpha }$ of the magnetograms for 16 ARs, where *k* being the spatial wavenumber, and compared $\alpha$ with the flare index *FI*, which represents the flare productivity of a given AR:

8 $$\begin{aligned} FI=\frac{1}{\tau } \left[ 100\sum _{i}I_{X}+10\sum _{j}I_{M}+1.0\sum _{k}I_{C}+0.1\sum _{l}I_{B} \right] , \end{aligned}$$

where $I_{X}$, $I_{M}$, $I_{C}$, and $I_{B}$ are the GOES magnitudes of X-, M-, C-, and B-classes, respectively, that occurred in a given AR in the period of $\tau$ days, and indices *i*, *j*, *k*, and *l* designate flares in each class. As shown in Fig. 25, it was revealed that higher flare productivity is associated with steeper spectrum: the power-law index is $\alpha >2.0$ for ARs producing X-class flares and is $\alpha \approx 5/3$ for flare-quiet ARs (i.e., regime of classical Kolmogorov turbulence; Kolmogorov 1941). Although not mentioned in Abramenko (2005), the above result might also be explained by the observation that larger ARs tend to produce stronger flares (e.g., Sammis et al. 2000): the spatial power spectrum of a large AR would have more power at low wavenumbers but have the same power at higher wavenumbers, which leads to a steeper power spectrum for a larger AR.

The works introduced in this subsubsection essentially show the fractal, multi-fractal, and/or turbulent nature of flaring ARs (Abramenko et al. 2002, 2003; Abramenko and Yurchyshyn 2010; McAteer et al. 2010; Georgoulis 2012). Regarding the practical flare prediction, Georgoulis (2005) revealed, however, that the fractal dimension does not have significant predictability. Rather, they suggested that the temporal evolution of the fractal diagnostics may be practically useful in flare prediction.

#### (Im)balance of electric currents

Magnetic energy that is released in solar flares stems from the non-potential, magnetic field associated with electrical currents. An important and long-standing question about the electric current is whether or not the current is neutralized in ARs, and, if not, to what extent and how (e.g., Melrose 1991, 1995, 1996; Parker 1996).

For the violation of current neutralization, two basic mechanisms have been proposed, which are (1) the magnetic field lines are stressed and twisted by photospheric and sub-photospheric flow motions (e.g., Klimchuk and Sturrock 1992; Török and Kliem 2003; Dalmasse et al. 2015); and (2) the current is provided by the emergence of twisted, i.e., current-carrying flux tubes (e.g., Leka et al. 1996; Longcope and Welsch 2000; Fan 2001b).

The current neutralization is investigated by examining whether the total electric current integrated over a single magnetic polarity of an AR vanishes. This is equivalent to whether the main (direct) current of a flux tube is surrounded by the shielding (return) current of equal strength and opposite direction. A number of observers have tried to address this issue by measuring the longitudinal (vertical) component of electric current density from the vector magnetogram,

9 $$\begin{aligned} j_{z}=\frac{c}{4\pi } \left[ {\mathbf {\nabla }}\times {{\mathbf {B}}} \right] _{z} =\frac{c}{4\pi } \left( \frac{\partial B_{y}}{\partial x} - \frac{\partial B_{x}}{\partial y} \right) , \end{aligned}$$

where *c* is the speed of light. Whereas Wilkinson et al. (1992) stated that their data do not convincingly show a non-neutralized current system, many observations have consistently suggested the existence of twisted flux systems, in favor of the scenario (2) (see a variety of observations introduced in previous sections). To cite a case, Wheatland (2000) examined vector magnetograms for 21 ARs and found that the electric currents in the positive and negative polarities significantly deviated from zero in more than half of the ARs studied, indicating that the AR currents are typically not neutralized. Using vector magnetograms of the highest quality by Hinode/SOT/SP, Georgoulis et al. (2012) investigated the distribution of currents in a flaring/eruptive AR (NOAA 10930) and a flare-quiet one (NOAA 10940). They found that substantial non-neutralized currents are injected along the photospheric PILs and that more intense PILs yield stronger non-neutralized currents. From statistical studies, Liu et al. (2017b) and Kontogiannis et al. (2017) showed that the flare- and CME-producing ARs are characterized by strong non-neutralized currents.

However, because the measurement of electric currents is strongly hampered by the limited resolution and ambiguities of magnetogram, it has always been a challenging task to accurately evaluate the distribution of currents as in Eq. (9). Therefore, to figure out whether the ARs are born with net currents, it is desirable to enlist the aid of numerical modeling (Török et al. 2014, see Sect. 4.1).

**Fig. 26:**
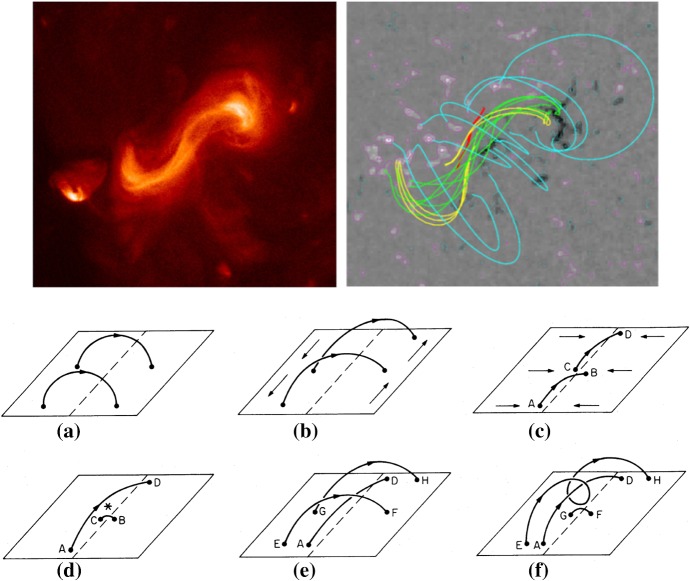
(Top left) Hinode/X-Ray Telescope (XRT; Golub et al. 2007) image of the sigmoid observed on February 12, 2007. (Top right) Field lines traced from the NLFFF extrapolation model. The cyan field lines belong to the potential arcade. The yellow J-shaped and the green S-shaped field lines are part of the flux rope, and the short red field lines lie under the flux rope. The background shows the LOS magnetogram. Image reproduced by permission from Savcheva et al. (2012a), copyright by AAS. (Bottom) Filament formation model based on the flux cancellation scenario. Field lines above the PIL (dashed line) become sheared and converged due to the photospheric motions (panels **a**–**c**). Magnetic reconnection then produces a long overlying loop (A–D in panel **d**) and a short field line that submerges (B–C). Overlying arcades are further sheared and converged to produce a flux rope (panels **e** and **f**). Image reproduced by permission from van Ballegooijen and Martens (1989), copyright by AAS

### Atmospheric and subsurface evolutions

#### Formation of flux ropes: sigmoids and filaments

In flare-productive ARs, free magnetic energy is stored in non-potential coronal fields that harbor significant amount of shear and twist. When observed in soft X-rays, these coronal fields display forward or inverse S-shaped structures, which was first observed by Acton et al. (1992) and are called “sigmoids” (Rust and Kumar 1996): see review by Gibson et al. (2006). Figure 26 (top) shows a typical example of a sigmoid. One may find that its structure is in good agreement with the extrapolated coronal fields, which shows the form of a magnetic flux rope. From the statistical analysis of the data from Yohkoh’s Soft X-ray Telescope (SXT; Tsuneta et al. 1991), Canfield et al. (1999) revealed that ARs are significantly more likely to be eruptive if they are either sigmoid or large: 51% of all ARs analyzed are sigmoid and they account for 65% of the observed eruptions. This result attracted interest in sigmoids as precursors of flare eruptions, and the trend was confirmed later by Canfield et al. (2007), Savcheva et al. (2014) and Kawabata et al. (2018).

Sigmoids are often accompanied by H$\alpha$ filaments (e.g., Pevtsov et al. 1996; Pevtsov 2002), and they form above and along the PILs in the evolving ARs. It is therefore important to understand the formation mechanism of sigmoids in relation to the large-scale/long-term evolution of the photospheric fields (as we saw earlier in Sects. 3.1 and 3.2). In fact, the series of sigmoid observations indicate that they are created in the manner anticipated in the filament formation model by van Ballegooijen and Martens (1989) [see Fig. 26(bottom)], in which the shearing and converging flow around the PIL drives flux cancellation and twists up the arcade fields to create a flux rope (see also Martens and Zwaan 2001).^6^

**Fig. 27:**
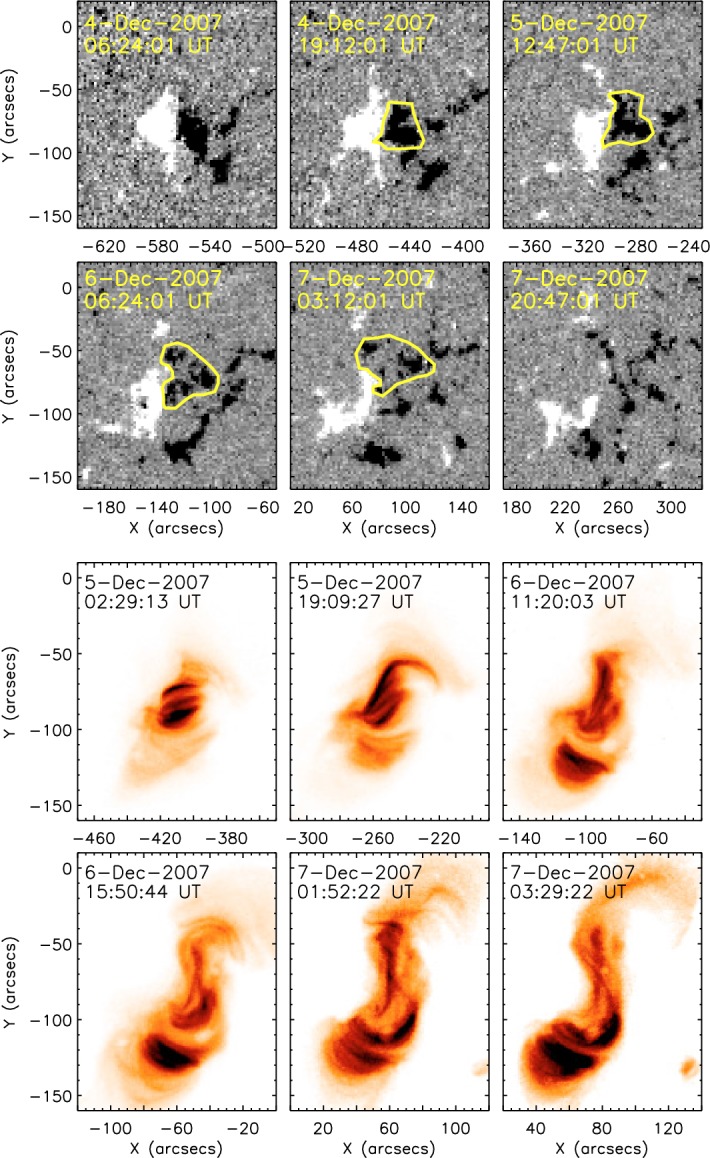
Day to day evolution of AR NOAA 10977. (Top) SOHO/MDI magnetogram saturating at $\pm \, 100\, \mathrm{G}$. (Bottom) Hinode/XRT C Poly filter images showing the transition from a sheared arcade to a sigmoid. Images reproduced by permission from Green et al. (2011), copyright by ESO

Figure 27 is one of the most compelling examples of the sigmoid formation through spot evolution (Green et al. 2011). At the central PIL of this AR, about one third of the magnetic flux cancels in 2.5 days before the flare eruption and the photospheric field shows an apparent shearing motion (top panels). At the same time, the coronal structure transforms first from a weakly to a highly sheared arcade then to a sigmoid that lies over the PIL (bottom panels). The sigmoid flux rope erupts eventually during the GOES B1.4-class flare, leaving an arcade structure in soft X-ray images (Sterling and Hudson 1997; Hudson et al. 1998; Sterling et al. 2000). A similar long-term transition of coronal fields from a sheared arcade or a pair of J-shaped loops to the sigmoid was also observed by Tripathi et al. (2009), Green and Kliem (2009) and Savcheva et al. (2012b). From these observations, one can infer that the twisted flux rope in a flaring AR is formed above the PIL due to the photospheric driving before the eruption.

Then, it is natural to speculate that magnetic helicity is the cause of the flux rope structure. To this end, Yamamoto et al. (2005) analyzed three sigmoid ARs and found that in two regions, the magnetic helicity injected through the sigmoid footpoints is comparable to the helicity content of the sigmoid loops. However, this is not true for the other AR, which may be because the sigmoid consists of multiple loops. They concluded that, excluding the latter complex AR, the magnetic twist of sigmoids is consistent with the helicity injected from the sigmoid footpoints. Investigating various filament eruption events associated with sigmoids, Green et al. (2007) showed that the structure of a sigmoid agrees with the helicity of a filament (e.g., forward S-shaped sigmoid for positive helicity filament) and that the rotation of a filament apex during the eruption is consistent with the helicity of the filament (e.g., clockwise rotation for positive helicity filament). The authors found that these behaviors agree with the kink instability scenario as numerically modeled by Török and Kliem (2005).

**Fig. 28:**
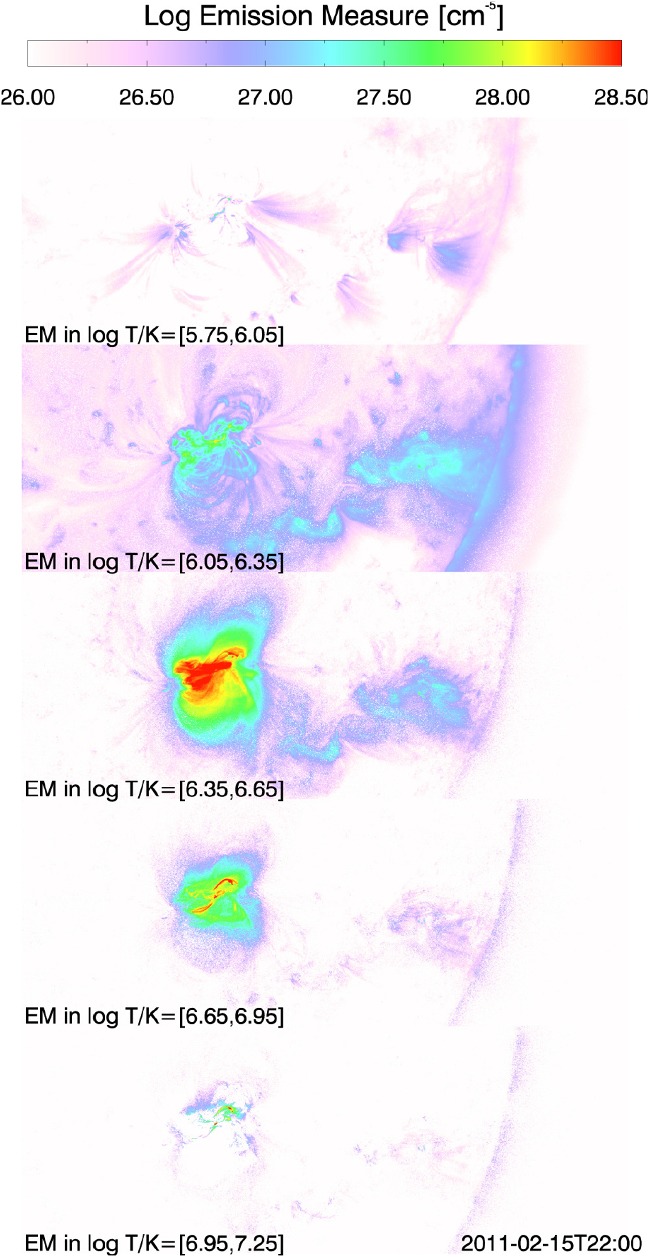
DEM maps of AR NOAA 11158 with the FOV of $1200''\times 480''$ centered at $(600'', -\,268'')$. The color indicates the total EM contained within a $\log {(T\, \mathrm{[K]})}$ range indicated in the bottom left corner of each panel. Image reproduced by permission from Cheung et al. (2015), copyright by AAS

Thermal structures of sigmoid ARs have been investigated by differential emission measure (DEM) analysis (for detailed account of this method, see Sects. 7 and 8 of Del Zanna and Mason 2018). For instance, the DEM maps of AR NOAA 11158 in Fig. 28, calculated from six EUV images of SDO/AIA by Cheung et al. (2015), clearly reveals that a hot core structure is embedded in the center of AR ($\log {(T \mathrm{[K]})}>6.6$) and covered by cooler overlying loops ($\log {(T \mathrm{[K]})}\lesssim 6.3$). Syntelis et al. (2016) analyzed the pre-eruptive phase of NOAA 11429, which is responsible for the two consecutive X-class flares with fast CMEs, using data from both AIA and Hinode’s EUV Imaging Spectrometer (EIS; Culhane et al. 2007). They found that the mean DEM of the flux ropes in the temperature range of $\log {(T \mathrm{[K]})}=6.8$–7.1 gradually increased by an order of magnitude about 5 h before the CME eruption. This increase was associated with the rising of the flux rope and may be related to the observed heating in CME cores (Cheng et al. 2012; Hannah and Kontar 2013), although the physical relationship with instabilities is not clear.

#### Broadening of EUV spectral lines prior to flares

Another possible atmospheric response to the photospheric evolution is the pre-flare non-thermal broadening of coronal EUV spectral lines. The observed line width consists of thermal width, instrumental width, and non-thermal (excess) broadening, which are related via

10 $$\begin{aligned} W_{\mathrm{obs}}^{2}=W_{\mathrm{inst}}^{2}+4\ln {2} \left( \frac{\lambda }{c} \right) ^{2} \left( v_{\mathrm{t}}^{2}+v_{\mathrm{nt}}^{2} \right) , \end{aligned}$$

where $W_{\mathrm{obs}}$ and $W_{\mathrm{inst}}$ are the observed and instrumental widths, respectively, $\lambda$ the wavelength of the emission line, *c* the speed of light, $v_{\mathrm{t}}$ the thermal velocity, and $v_{\mathrm{nt}}$ the non-thermal velocity.

**Fig. 29:**
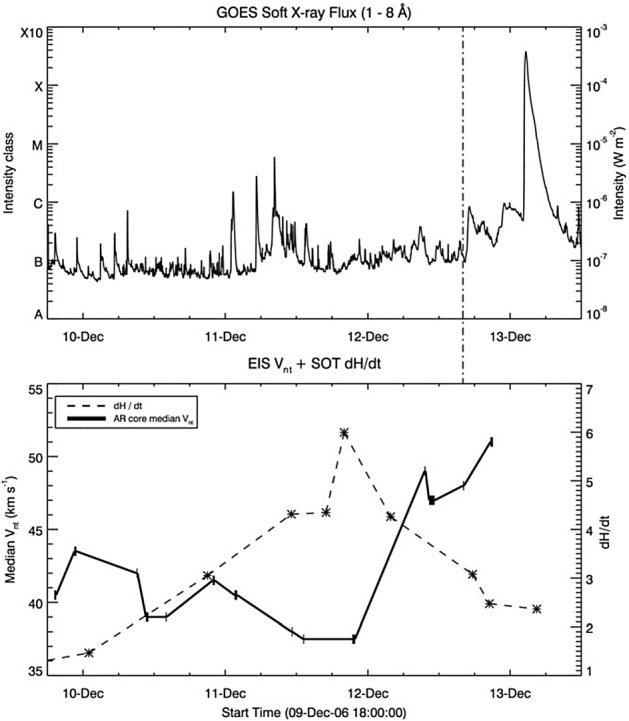
(Top) GOES soft X-ray light curve from December 9 to 13, 2006. The X3.4-class flare occurs at 02:14 UT on December 13. (Bottom) Helicity injection rate ($dH_{\mathrm{R}}/dt$) in the unit of $10^{36}\, \mathrm{Mx}^{2}\, \mathrm{s}^{-1}$, measured by Hinode/SOT/SP by Magara and Tsuneta (2008) (asterisks with dashed line). The median of the top 95th percentile of non-thermal velocities observed in the AR core ($v_{\mathrm{nt}}$) for Hinode/EIS Fe xii 195 Å line is also plotted (solid line). The vertical dash-dotted line denotes the time of the third EIS measurement of December 12. Image reproduced by permission from Harra et al. (2009), copyright by AAS

Alexander et al. (1998), Ranns et al. (2000) and Harra et al. (2001) showed that the non-thermal broadening peaks in the early phase of, or even tens of minutes, before the flare occurrence, and suggested that the broadening indicates turbulence that is related to the flare triggering mechanism. However, Harra et al. (2009) revealed that the pre-flare broadening starts much earlier. They measured the non-thermal velocity of Fe xii 195 Å line using Hinode/EIS and found that, as shown in Fig. 29, the increase in the line width begins up to one day before the X-class flare occurs after the helicity injection saturates (Magara and Tsuneta 2008). Imada et al. (2014) revisited this event and showed that this pre-flare broadening occurs in concurrence with upflow of about 10–$30\, \mathrm{km\ s}^{-1}$. They speculated that the upflow indicates the expansion of outer coronal loops and this rising motion (observed as the Doppler blueshift) causes the excess broadening.

#### Helioseismic signatures in the interior

**Fig. 30:**
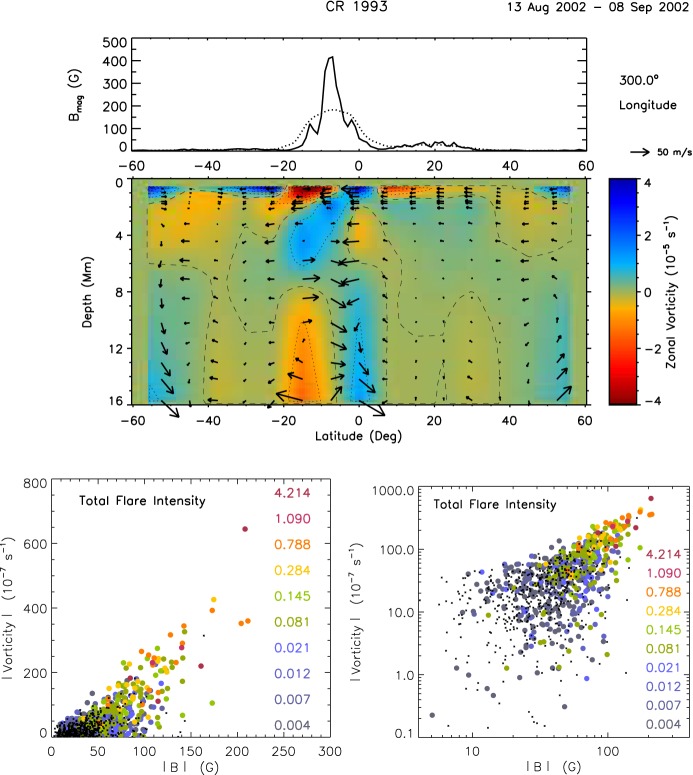
(Top) Vorticity distribution beneath a sample AR. The upper panel shows the latitudinal distribution of the unsigned magnetic flux across AR NOAA 10096 (solid) and that binned over 15$^{\circ }$ (dashed), whereas the lower panel displays the zonal vorticity component (the east–west component: $\omega _{x}$) as a function of latitude and depth, with arrows denoting the meridional flows. The strong zonal vorticity of opposite sign is concentrated at the location of the AR. Image reproduced by permission from Mason et al. (2006), copyright by AAS. (Bottom) Total flare intensity of ARs during their disk passage (in the unit of $10^{-3}\, \mathrm{W\ m}^{-2}$, i.e. relative to an X10 flare) as a function of unsigned maximum magnetic flux density and unsigned subsurface vorticity at $-\,12\, \mathrm{Mm}$, plotted in linear scale to focus on large values (left) and logarithmic scale to focus on small ones (right). The colors indicate the maximum intensity of each subset. Black symbols are non-flaring ARs. Image reproduced by permission from Komm and Hill (2009), copyright by AGU

Given the complex features of magnetic fields in flaring ARs, it is natural to ask if there is any subsurface counterpart. One of the earliest attempts to apply the local helioseismology techniques to search for the statistical relation between the subsurface flow field and the flare occurrence was done by Mason et al. (2006): Fig. 30 (top). They applied the ring-diagram method to 408 ARs from the Global Oscillation Network Group (GONG) data and 159 ARs from the SOHO/MDI data to measure the vorticity of flows (${\varvec{\omega }}=\nabla \times {{\mathbf {v}}}$) and compared it with the total flare intensity [equivalent to the flare index *FI*: Eq. (8)]. It was found that the maximum unsigned vorticity components at a depth of about 12 Mm, calculated from a synoptic maps of global subsurface flows that are generated by averaging the ring-diagram flow fields over 7 days (Haber et al. 2002), are correlated well with the flare intensity greater than $3.2\times 10^{-5}\, \mathrm{W\ m}^{-2}$. For flare activity below this value, the relation was not apparent. Komm and Hill (2009) expanded the analysis to 1009 ARs including non-flaring ones. As shown in the bottom panels of Fig. 30, they demonstrated a clear relation between the magnetic flux density (total magnetic flux averaged over area: in the unit of G) and vorticity for flaring ARs (correlation coefficient $CC=0.75$). The non-flaring ARs show a similar trend but the correlation is weaker ($CC=0.5$) and the mean values of flux and vorticity are smaller. The authors concluded that the inclusion of vorticity helps to distinguish between flaring and non-flaring regions.

Reinard et al. (2010) put more focus on the temporal evolution of subsurface flow fields. By analyzing 1023 ARs with the ring-diagram method, they showed that (1) at first, about 2–3 days before the flare occurrence, the kinetic helicity density, ${{\mathbf {v}}}\cdot {\varvec{\omega }}={{\mathbf {v}}}\cdot (\nabla \times {{\mathbf {v}}})$, has a large spread in values with depth, but the spread decreases on the days of the flares, and that (2) the degree of shrinking is greater for stronger flares. The observed tendency lends support to the interpretation that the subsurface rotational turbulent flows twist the magnetic fields into unstable configurations and drives the flare eruptions. Komm et al. (2011a) further applied discriminant analysis to various magnetic and subsurface flow parameters and found that the subsurface parameters improve the ability to distinguish between the flaring and non-flaring ARs. The most important parameter is the structure vorticity, which estimates the horizontal gradient of the horizontal vorticity components.

As an independent ring-diagram study, Lin (2014) compared the flare activity levels of 77 ARs and the quantities that describe the subsurface structural disturbances. According to the author, there was no remarkable correlation between these parameters.

Another approach is to apply time-distance helioseismology. Using the sequential SDO/HMI data of five flare-productive ARs, Gao et al. (2012, 2014) compared the kinetic helicity density measured from the subsurface velocity maps and the current helicity density calculated from the photospheric vector magnetograms, ${{\mathbf {B}}}\cdot (\nabla \times {{\mathbf {B}}})$,^7^ and found a good correlation between the two values. They found that eight out of a total of 11 events show a drastic amplitude change of the kinetic helicity density, and five of them are accompanied by flares stronger than M5.0 level within 8 h, either before or after the amplitude change. The spread of the kinetic helicity density in depth also showed strong variations, which confirms the observational result of Reinard et al. (2010).

Braun (2016) used helioseismic holography to more than 250 ARs observed between 2010 and 2014. They found that individual ARs show mostly variations associated with non-flare related evolution, although correlations between the flare soft X-ray flux and subsurface flow indices are in general similar to those found previously by Komm and Hill (2009). Moreover, they detected no remarkable precursors or other temporal changes that are specifically associated with the flare occurrences.

It should be pointed out that whereas not a small number of results have been reported, there is no clear physical model that explains the statistical correlations found between flaring and various properties of subsurface flows. For instance, it is not clear why the subsurface vorticity is correlated with AR flux, better for the flaring ARs than for the non-flaring ARs (Fig. 30). Therefore, further investigation, probably with the aid of numerical simulations, is required to interpret the observational results.

The difficulty resides also in the observational techniques. In many cases, the existence of strong magnetic flux (i.e. ARs) is assumed as a small perturbation when solving the linear inverse problem in seismology. However, this may not be true (see Gizon and Birch 2005, Sect. 3.7). Development of seismology techniques, again with the assistance of modeling, may overcome this shortcoming and deepen our understanding of subsurface evolutions.

### Summary of this section

In this section, we have reviewed the important observational characteristics that are created in the long-term and large-scale evolution of flare-productive ARs. Many of these characteristics manifest the morphological and magnetic complexity of such ARs and prove the inherent high non-potentiality of the magnetic system.

The $\delta$-spots, in which the umbrae of both polarities share a common penumbra (Sect. 2.3), are formed in three ways (Sect. 3.1): Type 1 (Spot-spot), the tightly packed sunspot with multiple bipoles intertwined; Type 2 (Spot-satellite), where a newly emerging flux appears in close proximity to a pre-existing spot; and Type 3 (Quadrupole), the head-on collision of two neighboring bipoles. However, X-class flares also emanate from between two separated ARs, albeit rarely (Inter-AR). The $\delta$-spots develop the strong-field, strong-gradient, highly-sheared PILs, which sometimes show a magnetic channel, a narrow lane structure consisting of elongated flux threads of opposite polarities (Sect. 3.2.1). These magnetic evolutions are caused by the shearing and converging flows around the PIL, where as remarkable sunspot rotations, both the self and mutual rotations, are also observed (Sect. 3.2.2).

Injection of magnetic helicity is found to have temporal correlation with flare productivity, while X-class flares require a significantly higher amount of helicity injection (Sect. 3.2.3). The magnetic tongue structure is thought to be the manifestation of emergence of twisted magnetic flux and is used as a proxy of magnetic helicity sign (Sect. 3.2.4). In studies addressing the old question of whether AR currents are neutralized or not, the preponderance of recent evidence supports the view that electric currents are not neutralized, particularly in regions prone to exhibit large flares (Sect. 3.2.5).

Twisted flux ropes, observed as H$\alpha$ filaments and soft X-ray sigmoids, can be produced in the atmosphere above the PILs due to the shearing and converging flows and helicity injection, which eventually erupt in the flares and evolves into CMEs (Sect. 3.3.1).

Though more extensive surveys are desired, several works have shown that flaring ARs have steeper power spectra, probably reflecting the morphological and magnetic complexity (Sect. 3.2.4), coronal upflows with excess broadening of EUV emission lines in response to the helicity injection (Sect. 3.3.2), and properties of vorticity in the convection zone (Sect. 3.3.3).

**Fig. 31:**
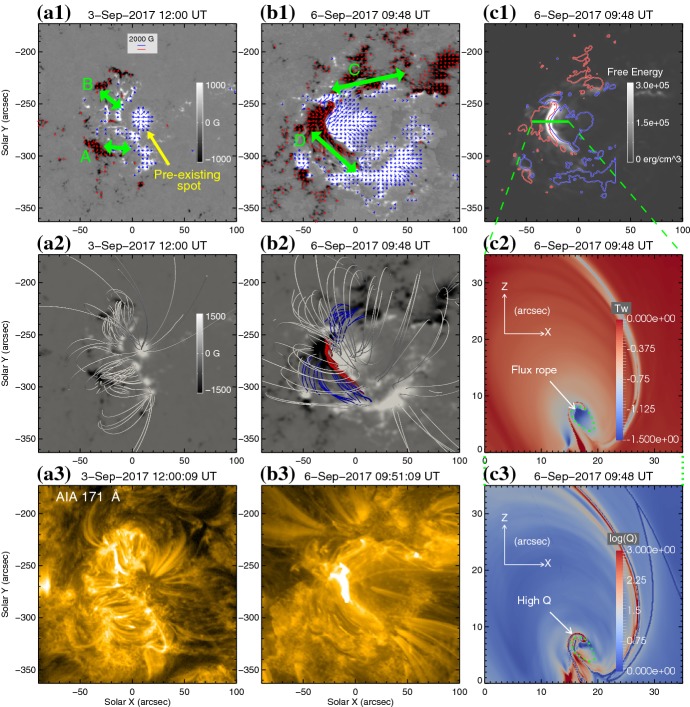
Evolution of AR NOAA 12673 and the formation of the flaring PIL. Image reproduced by permission from Yang et al. (2017a), copyright by AAS. **a1**–**a3** SDO/HMI vector magnetograms at 12:00 UT on September 3, top view of the extrapolated field lines, and corresponding AIA 171 Å image, respectively. **b1**–**b3** Similar to panels **a1**–**a3**, but for the time at 09:48 UT on September 6. In panels **a1** and **b1**, green arrows are overlaid to indicate bipoles A, B, C, and D, and yellow arrow shows the pre-existing sunspot. **c1** Free energy density corresponding to panel **b1** overlaid with the vertical magnetic field contours at $\pm \, 800\, \mathrm{G}$. Twist number $T_{w}$ (Berger and Prior 2006) and squashing factor *Q* (Demoulin et al. 1996; Titov et al. 2002) distribution in the *x*–*z* plane along the cut labeled in panel **c1**. In panel **b2**, the blue field lines connect the opposite patches of bipole C and bipole D, respectively, and the red field lines indicate a flux rope along the PIL. In panels **c2** and **c3**, the green dotted curves outline the general shape of the flux rope

AR NOAA 12673, which appeared in September 2017 and produced numerous flares including the X9.3-class event, is characteristic of the important features introduced in this section. Figure 31 by Yang et al. (2017a) shows the overall evolution and the formation of the flaring PIL. This AR rotates on to the visible disk as a simple $\alpha$-spot of positive polarity. On September 3, two bipolar systems A and B suddenly emerge to the east of the pre-existing central spot (panels a1–a3), and two additional bipoles C and D emerge more in the north–south direction within the first two pairs, forming a highly complex $\delta$-spot (panels b1–b3). This evolution reminds us of a Type-2 $\delta$-spot, but at the same time the collision of the secondary bipoles C and D is also reminiscent of the Type-3 structure. Sun and Norton (2017) pointed out that the emergence rate of this AR is one of the fastest emergence events ever observed.

**Fig. 32:**
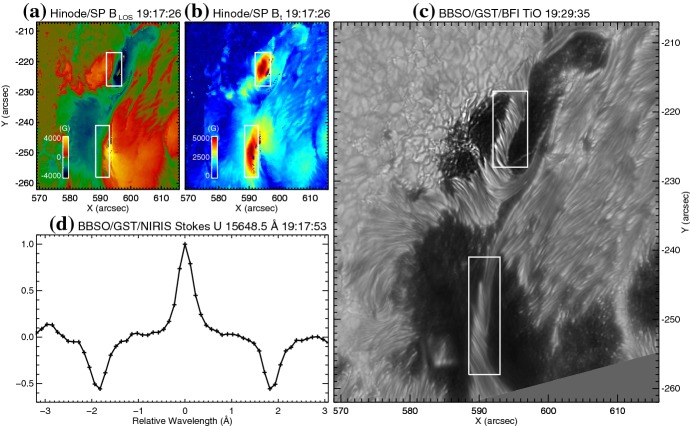
High-resolution observations of the flaring PIL of AR NOAA 12673. **a**, **b** Hinode/SOT/SP LOS and transverse magnetic field strength, respectively. Note that in many pixels near the PIL, transverse fields are saturated at 5000 G due to the limitation of inversion algorithm. **c** BBSO/GST TiO image. The two white boxes in **a**–**c** mark the two strong transverse field areas at the PIL, where twisted photospheric light-bridge structures of the $\delta$-configuration are present. **d** NIRIS Stokes-U profile of a selected strong transverse field pixel at the PIL within the northern box. The direct measurement of Zeeman splitting yields a field strength of 5570 G. Image reproduced by permission from Wang et al. (2018a), copyright by AAS

As the negative polarity of D rapidly intrudes into the positive polarities, it produces a strong-field, strong-gradient, highly-sheared PIL (Fig. 31: location where free energy is enhanced in panel c1). According to Yang et al. (2017a), because the pre-existing central spot blocks the free development of the newly-emerging fields, the $B_{z}$ gradient at the PIL becomes much enhanced. As Fig. 32 illustrates, Wang et al. (2018a) detected exceptionally strong transverse fields of up to $5570\, \mathrm{G}$ around this PIL. In the corona above this PIL, a flux rope structure is clearly reproduced by the NLFFF modeling (Fig. 31: red field lines in panel b2), which agrees well with the sigmoidal structure. Moreover, Verma (2018), Yan et al. (2018) and Vemareddy (2019) reported on the PIL shear flows, spot rotations, and helicity injection, respectively, which combined seems to activate the X9.3 flare.

## Long-term and large-scale evolution: theoretical aspects

As we saw in the preceding sections, in its long history of solar observation, a vast amount of key observational features that differentiate the flare-productive ARs from the quiescent ones have been discovered. The essential questions we have are, of course, how are they created and what is the underlying physics? The other side of solar physics, the theoretical and numerical studies, may provide answers to these questions.

Because there have already been substantial number of simulation models to date, in order to offer the reader a guideline, we introduce three genres of modeling, following the discussion in Cheung and Isobe (2014). The first group is the *data-inspired* models, which assume an ideal simulation setup that is “inspired” by the observations. Flux emergence and flux cancellation models fall into this group. The second group is the *data-constrained* models, in which the models use observational data at a single moment to drive computations. The series of extrapolated magnetic fields, computed from the sequential photospheric magnetogram, is one representative model of this group. However, it is less likely that such static solutions are applicable to flare-producing, i.e., dynamically evolving ARs. So, another way of the data-constrained models is to use the extrapolated field as the initial condition and solve the time-dependent MHD equations to trace the temporal evolution. The third group, the *data-driven* models, even utilizes a temporal sequence of observational data, such as the series of magnetograms, to drive the models.

The flux emergence and flux cancellation models are introduced in Sects. 4.1 and 4.2, respectively. The data-constrained and data-driven models, which are still rather the newcomers, are jointly shown in Sect. 4.3.

### Flux emergence models

The fundamental premise of the formation and evolution of flaring ARs is that solar ARs are produced ultimately by emerging flux from the convection zone. Therefore, it is not surprising that many theoretical models have focused on the evolution process of flaring ARs from below the surface of the Sun, which we call the flux emergence models. These models leverage the 3D flux emergence simulations, such as those in Sect. 2.1.4, and try to capture some aspects of observed magnetic features of flaring ARs. In fact, even classical models that configure a simple $\varOmega$-loop can explain some of the observed features.

- Magnetic tongues: As the series of observational studies predicted, magnetic tongues, the extended magnetic patches on the both sides of the PIL, are well reproduced by the emergence of a twisted flux tube (see, e.g., Fig. 4e). Archontis and Hood (2010) compared the magnetogram of AR NOAA 10808 and that produced in their numerical simulation and showed that the pattern of magnetic tongues depends on the azimuthal field of the emerging flux tube.
- Flux ropes and sigmoids: It was Manchester et al. (2004) who first reproduced the flux rope structures self-consistently in the 3D flux emergence simulation. In their model, where the buoyant segment of the flux tube is shorter than that of Fan (2001b)’s model, the upper part of the emerged flux tube becomes detached from the main body and forms a coronal flux rope that erupts into the higher atmosphere as in a CME. Archontis and Török (2008) explained the formation of a flux rope as magnetic reconnection between a set of emerging loops. Because the original flux tube is twisted, the emerged loops are sheared above the PIL and reconnect with each other, forming a flux rope structure. Archontis et al. (2009) revealed that the electric current sheets, which originally have a pair of J-shaped configurations, are joined to form a sigmoid structure as observed in soft X-rays. Similar sigmoid structure was observed in the models by, e.g., Magara (2006), Fan (2009b) and Archontis and Hood (2012).
- Shear flows: The essential driver of the shear flows in the emergence simulations is the Lorentz force on the two sides of the PIL in opposite directions (Manchester 2001). When the twisted flux tube emerges into the atmosphere, the rapid expansion deforms the field lines of the flux tube and drives the shear flows around the PIL. Fan (2001b) and Manchester et al. (2004) explained the twisting up of the coronal field as a shear Alfvén wave propagating upward, while Fan (2009b) interpreted it as a torsional Alfvén wave. The horizontal velocity vector of Fig. 4e clearly displays the shear flows around the PIL.
- Helicity injection: Injection of magnetic helicity flux through the photosphere was investigated by Magara and Longcope (2003), who revealed that in the earliest stage, the emergence term dominates, which then reduces and the shear term becomes the main source of the helicity injection for the rest of the period (see Sect. 3.2.3 for the definition of the terms). The helicity transport by the shear term is explained by the horizontal shearing and rotational motions at the footpoints of the emerged magnetic fields (Longcope and Welsch 2000; Fan 2009b).
- Spot rotation: This can be considered as the subtopic of the helicity injection. Longcope and Welsch (2000) proposed a theoretical model that treats both the expanded twisted flux tube in the corona and that remaining in the convection zone. In this model, as a twisted tube emerges, the torsional Alfvén wave propagates downward into the convection zone due to the mismatch of twists between the two layers and causes the spot rotation. Magara and Longcope (2003) and Magara (2006) found that the rotational flows are formed in each of the spots soon after the rising flux tube becomes vertical, whereas Fan (2009b) shows that significant vortical motions develop as a torsional Alfvén wave propagates along the flux tube. Sturrock et al. (2015) used a toroidal tube model (Hood et al. 2009) and revealed that two sunspots do undergo rotation (not an apparent effect). They explained the rotation by unbalanced torque produced by magnetic tension.
- (Im)balance of electric currents: Török et al. (2014) considered the emergence of a flux tube that contains neutralized electric currents (i.e., the situation where the direct current along the axis is balanced with the return current at the tube’s periphery). As the significant emergence to the surface begins, the current rapidly deviates from the neutralized state and the total direct current remains several times larger than that of the return current throughout the whole evolution. They suggested that when the tube approaches the surface, the return current is pushed aside by the direct current. Also, most of the return currents remain beneath the surface because the tube does not undergo a bodily emergence. It was therefore concluded that ARs are born on the surface with substantial net electric currents.

The above features are formed as parts of relaxation processes in which the twist of the flux tube is released through the emergence from the convection zone to the corona. However, in most of the these numerical models that assume a simple buoyant emergence of flux tubes, other important characteristics of flaring ARs, such as tightly-packed $\delta$-spots with strong-field, strong-gradient, highly-sheared PILs, are not reproduced. The two photospheric footpoints of the emerging $\varOmega$-loops are prone to separation in a monotonous fashion and never form a converged, $\delta$-shaped structure. Therefore, to overcome this difficulty, one needs to assume subsurface magnetic fields with *not-so-simple* configurations.

#### Kinked tube model

The idea of the emergence of a kink-unstable magnetic flux tube is inspired by the observations of flare-productive ARs, especially of Type 1 $\delta$-spots (see Sect. 3.1). These regions have compact morphology and strong twists, and the tilt often deviates so much from parallel to the equator that sometimes it even violates Hale’s polarity rule. The 3D configurations inferred from the proper motion of the spots strongly suggest the emergence of “a knotted twisted flux tube” (Tanaka 1991, see Fig. 15a of this article).

**Fig. 33:**
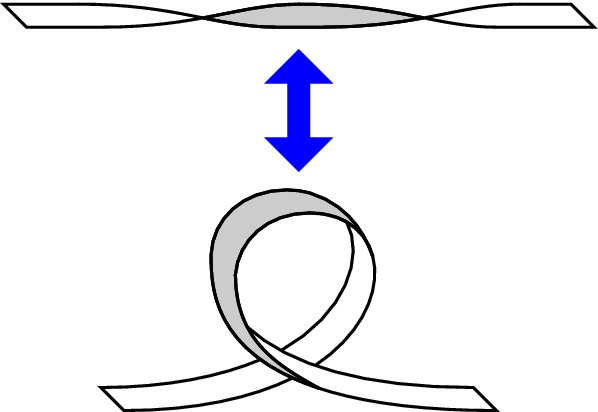
Conversion of twist and writhe. When a straight twisted ribbon (top) is loosened, the original twist converts into the writhe of the coiled ribbon (bottom). In an analogous way, a twisted flux tube deforms into a curled shape if the twist is sufficiently strong, which is the helical kink instability

According to Kurokawa (1991), it was Piddington (1974) who first proposed the concept of emerging twisted flux tubes for the energy source in the Alfvén wave theory of solar flares. In “Appendix”, we show the history about who suggested the kink instability first as the formation mechanism of the $\delta$-spots.

The helical kink instability is the instability of a highly-twisted flux tube, in which the twist of the tube (turning of the field lines around the tube’s axis) is converted to writhe (turning of the axis itself) due to the helicity conservation (see Fig. 33: Berger and Field 1984; Moffatt and Ricca 1992). It was applied to laboratory plasma (e.g., Shafranov 1957; Kruskal et al. 1958) and to coronal plasma (e.g., Gold and Hoyle 1960; Anzer 1968; Raadu 1972; Hood and Priest 1980, 1981), before Linton et al. (1996) considered the kink instability of flux tubes in a high-$\beta$ plasma.^8^ For a uniformly twisted cylindrical flux tube with the axial and azimuthal fields of $B_{x}(r)$ and $B_{\phi }(r)=qrB_{x}(r)$, respectively, where *r* is the radial distance from the tube’s axis and the twist *q* is constant, the flux tube becomes unstable against the kink instability when *q* exceeds a critical value

11 $$\begin{aligned} q_{\mathrm{cr}}=a^{-1}, \end{aligned}$$

where $a^{-2}$ is the coefficient for the $r^{2}$ term in the Taylor series expansion of the axial field $B_{x}$ about the flux tube: $B_{x}(r)=B_{\mathrm{tube}}(1-a^{-2}r^{2}+\cdots )$. In the case of commonly used Gaussian flux tubes, in which

12 $$\begin{aligned} B_{x}(r)=B_{\mathrm{tube}}\exp {\left( -\frac{r^{2}}{R_{\mathrm{tube}}^{2}}\right) } \end{aligned}$$

and

13 $$\begin{aligned} B_{\phi }(r)=qrB_{x}(r), \end{aligned}$$

with $R_{\mathrm{tube}}$ being the typical radius of the tube, the critical twist is simply expressed as $q_{\mathrm{cr}}=R_{\mathrm{tube}}^{-1}$. Linton et al. (1996) also argued that, as the flux tube rises through the convection zone, the originally stable tube may become unstable because the tube expands ($R_{\mathrm{tube}}$ increases) due to the decreasing surrounding pressure, which lowers the critical twist ($q_{\mathrm{cr}}$ decreases).

**Fig. 34:**
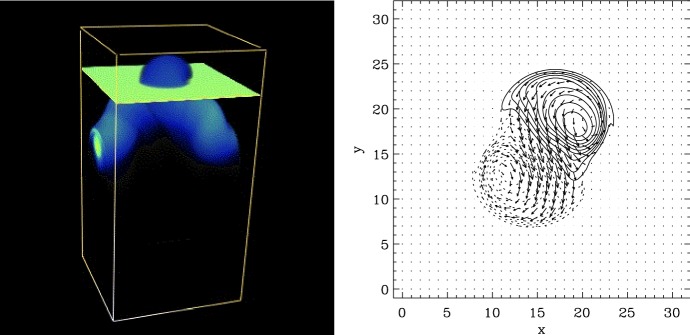
Emergence of a kink-unstable flux tube. Image reproduced by permission from Fan et al. (1998b), copyright by AAS. (Left) Snapshot of the flux tube during its rise as viewed from the side. The color shading indicates the absolute magnetic field strength. (Right) Horizontal cross-section of the upper portion of the flux tube (indicated by yellow plane in the left panel). The contours denote the vertical magnetic field $B_{z}$ with solid line (dotted line) contours representing positive (negative) $B_{z}$. The arrows show the horizontal magnetic field

The first 3D non-linear simulation of the kink-unstable emergence was done by Matsumoto et al. (1998) for reproducing the sequence of sigmoid ARs (top left panel of Fig. 26). Linton et al. (1998, 1999) performed linear and nonlinear calculations of the kink instability in a uniform medium without taking into account the effects of gravity and stratification of external plasma. Using the 3D anelastic MHD code, Fan et al. (1998b, 1999) calculated the emergence in an adiabatically stratified atmosphere representing the solar convection zone (Fig. 34) and found that, due to the kink instability, the writhing of the tube increases the buoyancy at the apex and accelerates the emergence. The horizontal cross-section of the tube shows a compact bipolar pair of $B_{z}$ with a highly sheared horizontal field along the PIL, and the line connecting the two polarities is deflected by more than 90$^{\circ }$ from its original orientation. These structures are highly reminiscent of the $\delta$-spots.

**Fig. 35:**
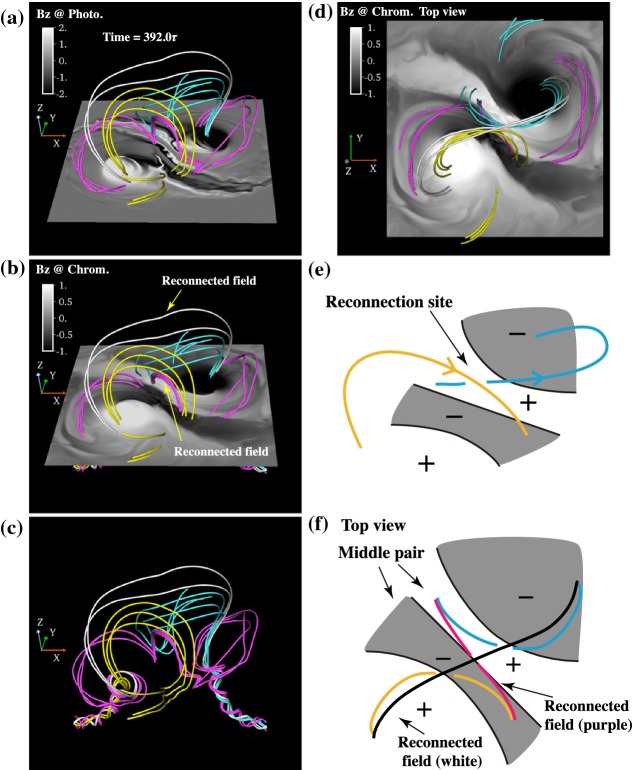
3D magnetic structure and photospheric and chromospheric fields $B_{z}$. Yellow and blue field lines denote the field lines passing by the current sheet between the two arcades. White field lines denote those enveloping the arcade. Purple and white field lines denote those created by reconnection between the blue and yellow magnetic loops. **a**–**c** Bird’s eye view. **d** Top view. **e** Schematic diagram of the magnetic field lines. **f** Schematic diagram of the magnetic field structure shown in panel **d**. Images reproduced by permission from Takasao et al. (2015), copyright by AAS. (For movie see Electronic Supplementary Material)

However, because these emergence simulations were confined to the convection zone, it remained unclear if the kinked tubes can really produce observed characteristics when they emerge into the atmosphere. To overcome this issue, Takasao et al. (2015) performed a fully compressible MHD simulation in which a subsurface kink-unstable flux tube rises from the convection zone seamlessly into the solar corona. In their model, the rising flux tube develops a knotted structure as in the previous simulations (e.g., Fan et al. 1998b; Linton et al. 1999) and, at the top-most convection zone, it undergoes a strong horizontal expansion due to the strong stratification and deforms into a pancake-like shape (two-step emergence, a commonly observed feature of large-scale flux emergence models: see Sect. 2.1.1 and Fig. 2). Interestingly, as opposed to the simple bipolar structure observed in the kinked tube simulations limited to the convection zone (right panel of Fig. 34), the photospheric magnetogram in Fig. 35 shows a quadrupolar structure consisting of the main bipolar pair of large roundish spots that appears in the earlier phase and the narrow, elongated middle pair formed later. The middle pair is created due to the submergence of dipped fields, which is a part of the emerged magnetic fields (see also the accompanying movie). The field lines in Fig. 35 show that magnetic reconnection takes place between the two emerging loops (blue and yellow field lines) and creates lower-lying and overlying post-reconnection field lines (purple and white field lines, respectively). Here, the lower-lying fields are almost parallel to the central PIL. It is also found that, as a consequence of Lorentz force exerted by the two emerging loops (expanding arcades) on both sides of the central PIL, a strong converging flow is excited around it and the horizontal magnetic field becomes aligned more parallel to it.

Later, Knizhnik et al. (2018) surveyed the evolution of kink-unstable tubes with varying the twist intensity. They revealed, for example, that the separation of both polarities on the surface becomes smaller (i.e., more compact) with increasing the twist, which underpins the kink instability as a promising candidate for explaining $\delta$-spot formation.

It should be noted that the assumed twists in these simulations may be too strong compared to the twists of the actual ARs. Pevtsov et al. (1994, 1995) quantified the twist of ARs by calculating the force-free parameter $\alpha$, the constant of a force-free field $\nabla \times {{\mathbf {B}}}=\alpha {{\mathbf {B}}}$ (see Sect. 4.3.1) measured from the vector field as

14 $$\begin{aligned} \alpha =\frac{\left[ \nabla \times {{\mathbf {B}}}\right] _{z}}{B_{z}} =\frac{1}{B_{z}} \left( \frac{\partial B_{x}}{\partial y}-\frac{\partial B_{y}}{\partial x} \right) , \end{aligned}$$

and averaging it over the AR to obtain one global estimate of the twist. The observed $\alpha$ is typically of the order of 0.01–$0.1\, \mathrm{Mm}^{-1}$ (e.g., Pevtsov et al. 1995; Leka et al. 1996; Longcope et al. 1998), which yields $q\lesssim 0.1\, \mathrm{Mm}^{-1}$ under the simple relation of $\alpha \approx 2q$ (Longcope and Klapper 1997), though there remains a possibility that the observed ARs are inclined to regular, flare-quiet ones due to selection bias. On the other hand, the threshold twist for the kink instability is, say, $q_{\mathrm{cr}}=1\, \mathrm{Mm}^{-1}$ for the typical tube radius of 1 Mm in the deeper convection zone. Therefore, the twists of the flux tubes assumed in the simulations, $q>q_{\mathrm{cr}}=1\, \mathrm{Mm}^{-1}$, are at least one order of magnitude larger than the observed AR twists, $q\lesssim 0.1\, \mathrm{Mm}^{-1}$, even though each elementary bipole in ARs may satisfy the assumed condition (Longcope et al. 1999).

#### Multi-buoyant segment model

Type 3 $\delta$-spots like the quadrupolar AR NOAA 11158 (Fig. 14), in which two emerging bipoles collide against each other to form a $\delta$-structure with a flaring PIL in between, are redolent of a subsurface linkage of the two bipoles. That is, the observed bipoles are the two emerging sections of a single subsurface flux system, distorted perhaps by convective buffeting during its rise (Fig. 15c).

**Fig. 36:**
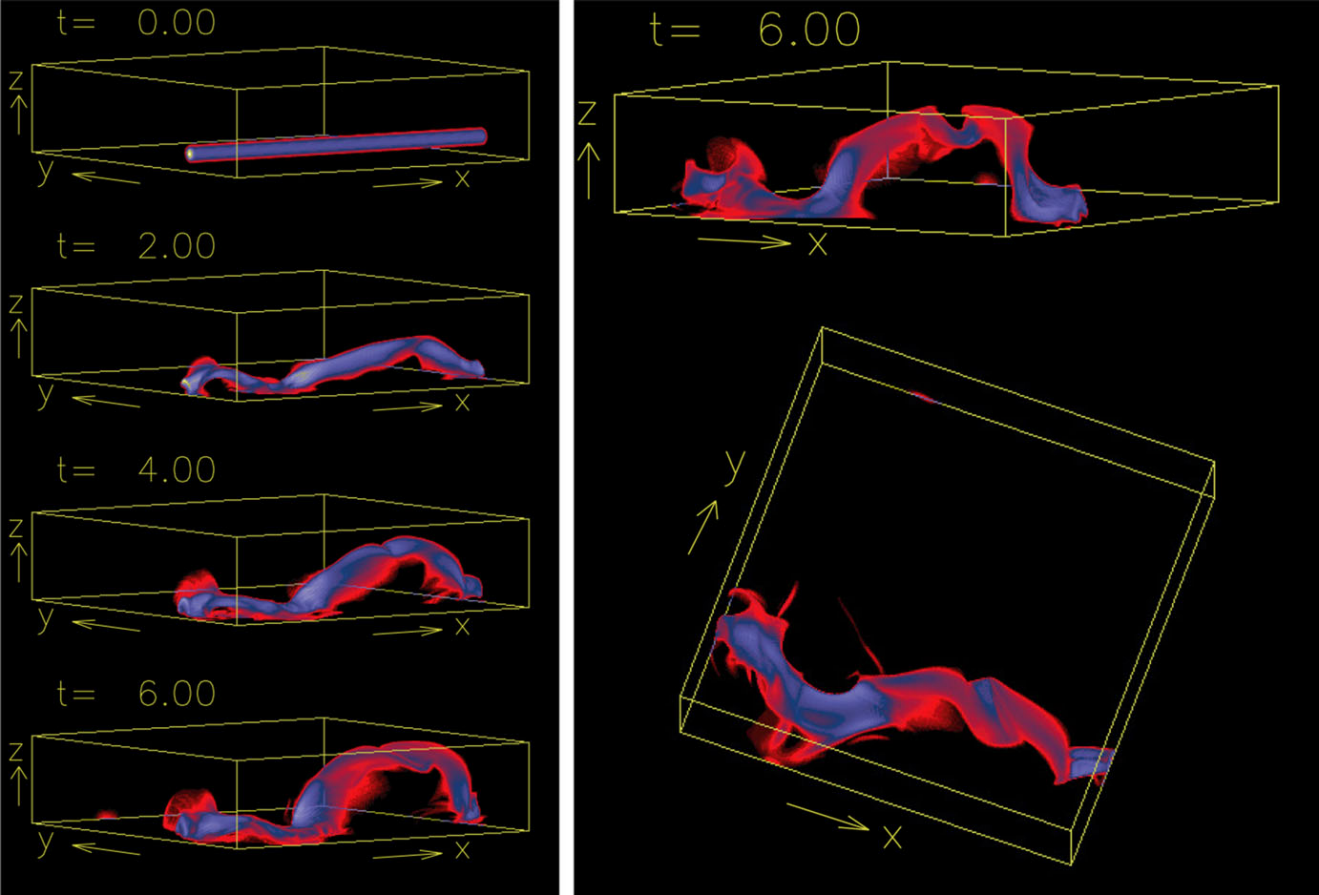
(Left) Evolution of the buoyant flux tube in the 3D convective flow for the case where the initial axial field is comparable to the equipartition field ($B_{\mathrm{tube}}=B_{\mathrm{eq}}$). The image shows the volume rendering of the absolute magnetic field strength of the flux tube. (Right) Two different views of the same tube at the final state, showing that the apex is pushed down by a local downflow. Image reproduced by permission from Fan et al. (2003), copyright by AAS

An emerging flux tube can be affected by the convection when the hydrodynamic force dominates the restoring magnetic tension of the bent flux tube (Fan 2009a):

15 $$\begin{aligned} \frac{B_{\mathrm{tube}}^{2}}{4\pi L} \lesssim C_{\mathrm{D}} \frac{\rho v^{2}}{\pi R_{\mathrm{tube}}}, \end{aligned}$$

which yields

16 $$\begin{aligned} B_{\mathrm{tube}} \lesssim \left( \frac{C_{\mathrm{D}}}{\pi } \frac{L}{R_{\mathrm{tube}}} \right) ^{1/2} B_{\mathrm{eq}} \sim \mathrm{a\ few}\ B_{\mathrm{eq}}, \end{aligned}$$

where $B_{\mathrm{eq}}=(4\pi \rho )^{1/2}v$ is the equipartition field strength, at which the magnetic energy density is comparable to the kinetic energy density of convective flows, $B_{\mathrm{eq}}^{2}/(8\pi )=\rho v^{2}/2$, *L* and *v* are the size scale and speed of the convection, respectively, and $C_{\mathrm{D}}$ is the aerodynamic drag coefficient, which is of order unity. At the bottom of the convection zone, $(L/R_{\mathrm{tube}})^{1/2}=3$–5 and $B_{\mathrm{eq}}\sim 10\, \mathrm{kG}$ (Fan 2009a). In fact, Fan et al. (2003) numerically demonstrated that flux tubes of $B_{\mathrm{tube}}\sim B_{\mathrm{eq}}$ are significantly influenced by turbulent convection. As Fig. 36 shows, the section of the emerging flux tube within convective upflows is strongly pushed up while the downdraft sections are pinned down. To make things intriguing, the apex of the rising $\varOmega$-tube encounters another local downdraft and takes an M-shaped structure.

**Fig. 37:**
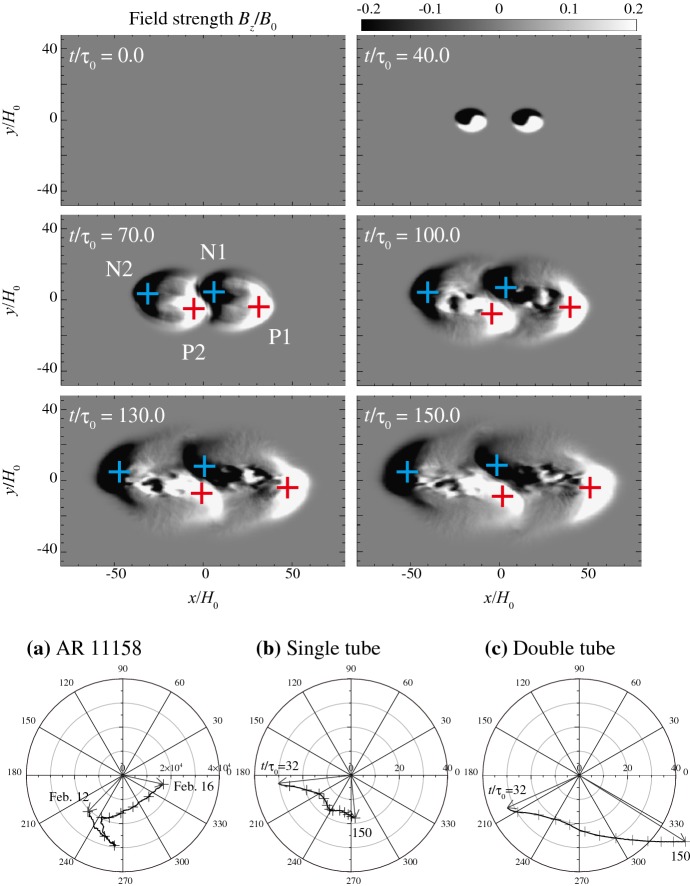
(Top) Emergence of a double-buoyant segment flux tube. The shown are the temporal evolution of vertical fields at the surface (photospheric magnetogram). Two emerging bipoles P1–N1 and P2–N2 collide at the center and form a sheared PIL with a compact $\delta$-spot structure. (Bottom) Relative motion of the photospheric polarities N1 and P2 for **a** AR NOAA 11158 (Fig. 14), **b** the simulation with a single double-buoyant-segment tube (i.e., top panels), and **c** another simulation with two parallel tubes. The center of each diagram indicates the position of N1 and the horizontal axis is parallel to the *x*-axis. Approaching of the two polarities in NOAA 11158 is reproduced only in the single tube model. Image reproduced by permission from Toriumi et al. (2014b), copyright by Springer

Such a situation was modeled by Toriumi et al. (2014b), who reproduced NOAA 11158 (Fig. 14) by simulating the emergence of a single horizontal flux tube that rises at two sections along the tube. As the photospheric magnetogram of Fig. 37 (top) displays, the two buoyant segments produce a pair of emerging bipoles P1–N1 and P2–N2, and the inner polarities (N1 and P2) become tightly packed to create a $\delta$-spot. The strong confinement of the central polarities happens because the two emerging loops (P1–N1 and P2–N2) are joined by a dipped field beneath the photosphere.

These authors also modeled the emergence of two buoyant flux tubes that are placed closely in parallel (but not connected). In this case, the inner polarities of the two emerging bipoles move closer but just fly-by and never form a compact $\delta$-spot. Bottom of Fig. 37 compares the relative motion of the two inner polarities (time evolution of the vector from N1 to P2) for NOAA 11158, the single tube case, and the double tube case. In the actual AR (see also Fig. 14), P2 continuously drifts along the southern edge of N1 from east to west in a counter-clockwise direction and becomes closer to N1, producing a highly-sheared, strong-gradient PIL. Between the two simulation cases, only the single tube case shows the monotonic decrease of the distance. Therefore, they concluded that this Type 3 quadrupolar AR is, between the two scenarios, more likely to be created from a single multi-buoyant-segment flux tube.

**Fig. 38:**
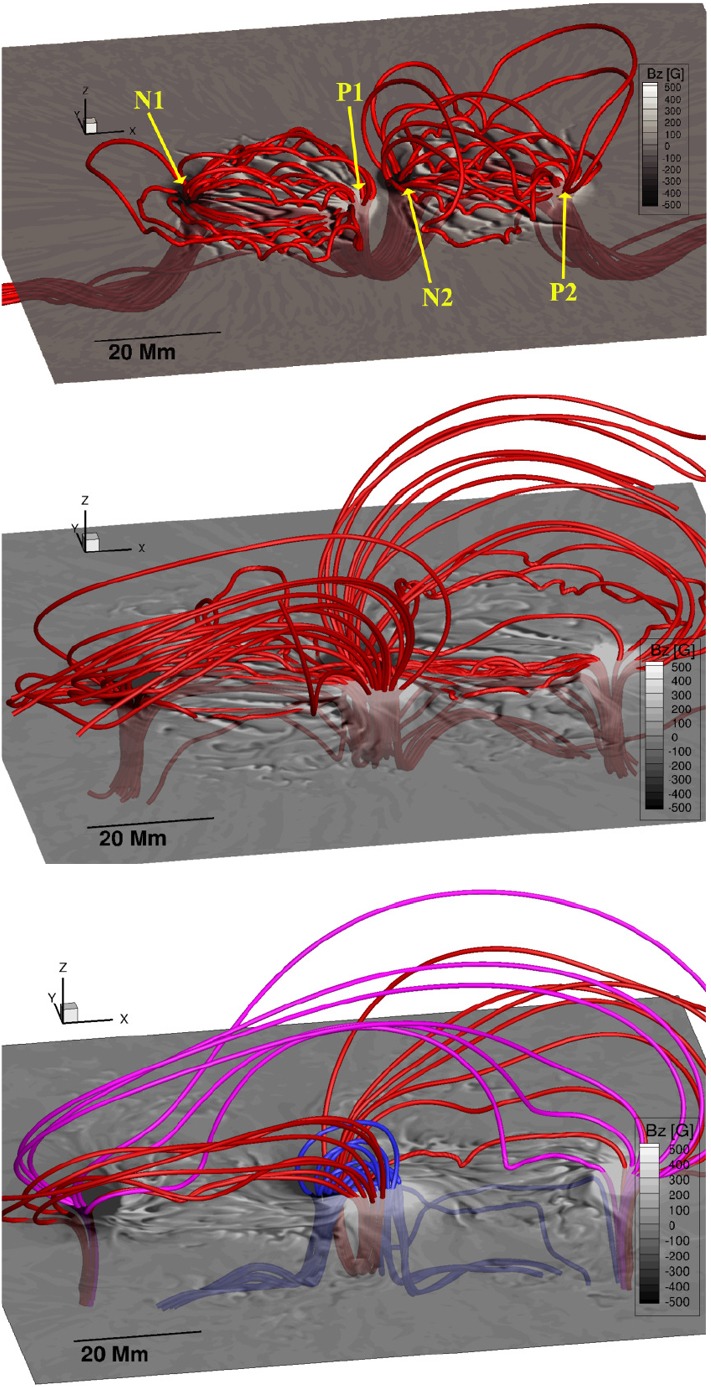
Simulation results by Fang and Fan (2015), showing 3D structure of the M-shaped emerging loops (red lines) at three different time steps. The plane shows the photospheric magnetogram. Note that the notation of the four polarities is different from that in Figs. 14 and 37. In the final state, magnetic reconnection between the two loops (red) produces overlying (magenta) and low-lying (blue) field lines. Image reproduced by permission, copyright by AAS

Exactly the same situation was investigated later by Fang and Fan (2015), but in a much larger computational domain of a realistic AR size with an adaptive mesh refinement code to resolve fine-scale structures. Figure 38 shows three snapshots from their simulation, which clearly shows that the M-shaped emerging loop produces two arcades in the corona and, through magnetic reconnection, overlying and lower-lying field lines, which is expected from the coronal observation of NOAA 11158 (see bottom panels of Fig. 14). The striking consistency between the more realistic simulation and the observation further supports the scenario of multi-buoyant-segment flux tubes for the Type 3 $\delta$-spots.

#### Interacting tube model

Another possible origin of the complexity of ARs is the subsurface interaction of multiple rising flux systems. Based on the study of potential flow around circular cylinders, Parker (1978, 1979b) predicted that when two cylindrical flux tubes are rising in a fluid one above the other, the lower tube is attracted toward the other because of the wake of the tube ahead and, when rising side by side, the tubes attract each other due to the Bernoulli effect. However, from 2D simulations on the cross-sectional evolution, Fan et al. (1998a) found that the interaction of the two tubes is much more complicated. When the tubes rise side by side, because the wake behind each tube interacts with that of the other, each tube sheds a succession of eddies of alternating signs and gains Magnus force in the lateral direction, leading to the repeated attractive and repulsive motions during their ascents. On the other hand, when the tubes do not have the same initial height, the tube behind is drawn into the wake of the tube ahead and eventually merges with it. At the interface between the two tubes, dissipation of oppositely directed field components (twists) occurs.

**Fig. 39:**
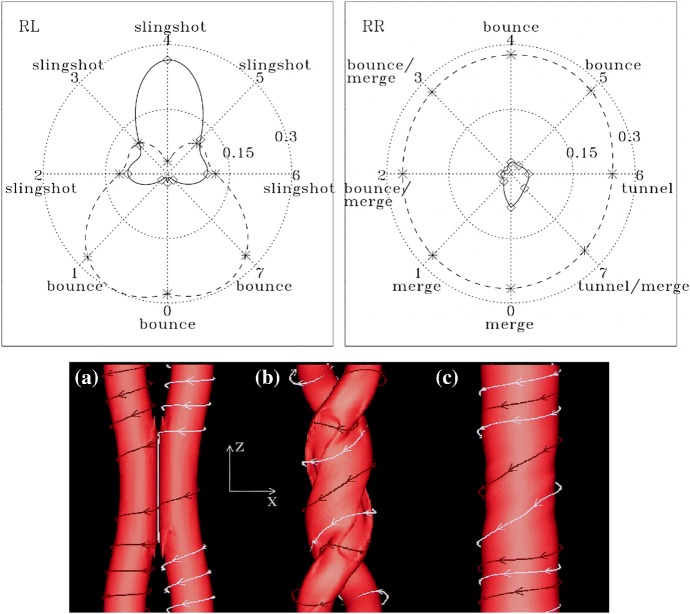
(Top) Polar plots showing the types of interaction of right-handed (R) and left-handed (L) twist tubes. Each radial spoke corresponds to a simulation RL*i*, where one R tube is in the reference position and another tube is in front of it, rotated by an angle $i\pi /4$ clockwise to it in such a way that RL0/RR0 is parallel and RL4/RR4 is anti-parallel. The solid curves show $2(\mathrm{KE}_{\mathrm{peak}}-\mathrm{KE}_{0})/\mathrm{ME}_{0}$, where KE$_{\mathrm{peak}}$ is the peak global kinetic energy during the simulation, KE$_{0}$ is the initial global kinetic energy, and ME$_{0}$ is the initial global magnetic energy. The dashed curves show the global magnetic energy near the end of the simulations normalized by ME$_{0}$. The dotted circles are the normalized energy levels of 0.15 and 0.3. (Bottom) Merge interaction of RR0. Isosurface of $|B|_{\mathrm{max}}/3$ and field lines for three time steps are shown. Image reproduced by permission from Linton et al. (2001), copyright by AAS

Linton et al. (2001) focused more on magnetic reconnection between two strongly-twisted flux tubes in the 3D low-$\beta$ volume (i.e., the solar corona) to study the triggering of flares and eruptions. They found that, depending on the helicity (twist handedness) and the relative angle of the tube axes, the interaction can be classified into four distinct classes (see Fig. 39): (1) bounce, in which the two tubes bounce off each other with very little reconnection, occurring for example between parallel counter-helicity tubes (RL0); (2) merge, in which the tubes merge due to reconnection of azimuthal components, e.g., between parallel co-helicity tubes (RR0: bottom of Fig. 39); (3) slingshot, in which the tubes reconnect and “slingshot” away in a manner analogous to the classical 2D reconnection, e.g., between anti-parallel counter-helicity tubes (RL4); and (4) tunnel, in which field lines of the tubes undergo reconnection twice and the tubes pass through each other, occurring when the co-helicity tubes are placed in the orthogonal direction like RR6. These interactions were also investigated by Sakai and Koide (1992). Linton and Antiochos (2005) and Linton (2006) demonstrated that the situations may differ depending on the level of twist and the balance of magnetic flux contained in the two tubes.

**Fig. 40:**
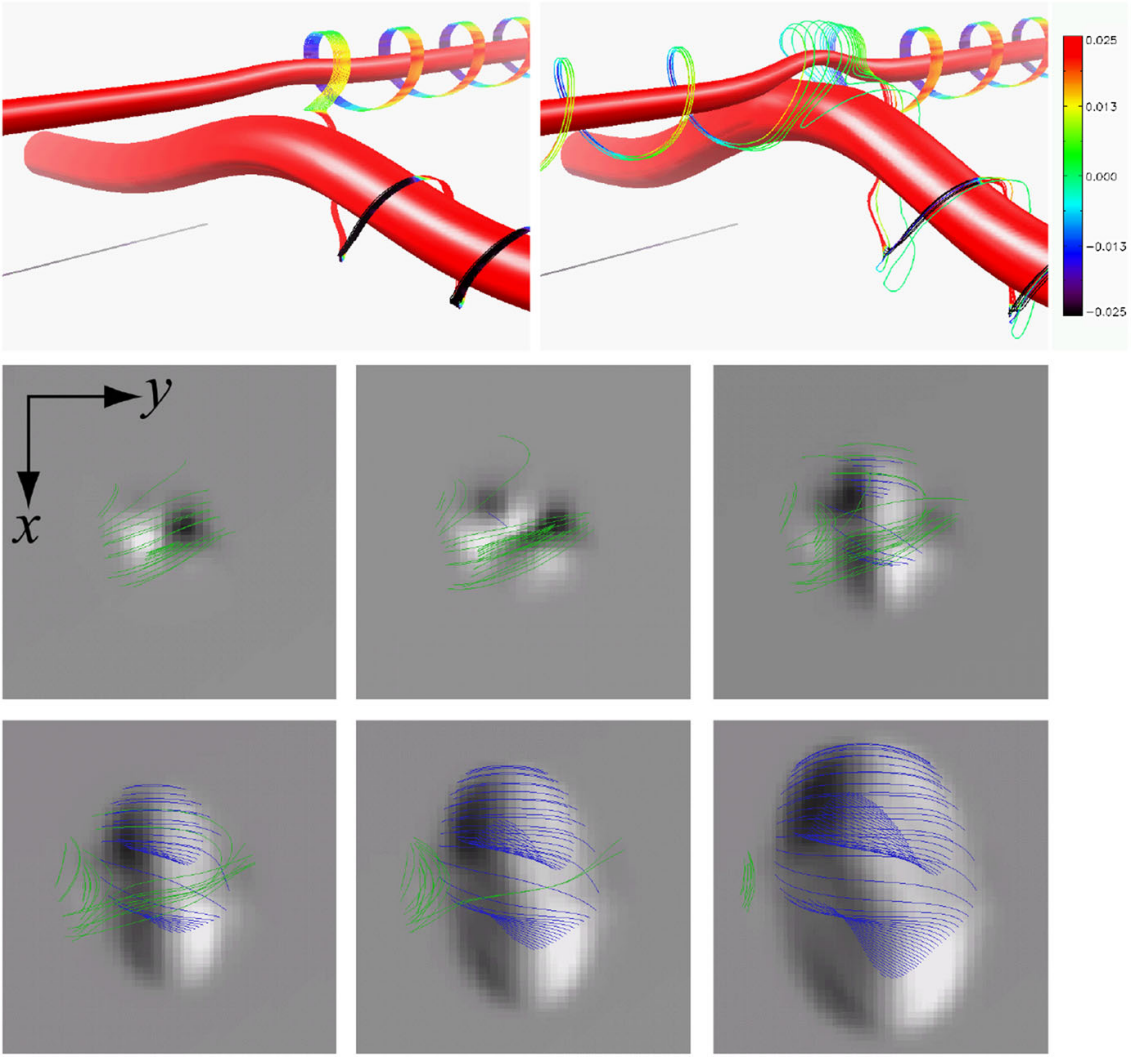
(Top) Two snapshots from the simulation of interacting orthogonal flux tubes. The field lines are colored according to local $B_{z}$, while the red isosurface gives a constant-|*B*| layer. (Bottom) Synthesized magnetogram at the photospheric height, in which darker and lighter colors represent $B_{z}<0$ and $B_{z}>0$, respectively. The green and blue lines are selected field lines, traced from the upper and lower tubes, respectively. Image reproduced by permission from Murray and Hood (2007), copyright by ESO

Murray and Hood (2007) simulated the interaction of emerging flux tubes in the stratified high-$\beta$ medium representing the solar interior. They examined the cases where two horizontal tubes are placed in such a way that the lower one is buoyant whereas the upper one remains stable. For the case of parallel tubes, or LL0 (the mirror symmetry of RR0) following the notation by Linton et al. (2001), they found that the tubes gradually merge, though not totally, and the photospheric magnetogram shows a simple ying–yang pattern similar to that of the single tube case (like in Fig. 4). Of more interest is the case with orthogonal tubes in Fig. 40, or LL2 (corresponding to RR6), where the two tubes are expected to perform a slingshot reconnection due to their lower degrees of twist (Linton 2006). The authors found that, as opposed to the expectation, the two tubes do not undergo a complete slingshot because the tubes differ much in strength. The resultant magnetogram becomes much more complicated. As Fig. 40 illustrates, the polarity layout is at first positive negative from left to right when the upper tube emerges. However, as the lower tube reaches the photosphere, the layout reveals a quadrupolar structure and transits to negative positive, eventually recovering the classical ying–yang pattern.

**Fig. 41:**
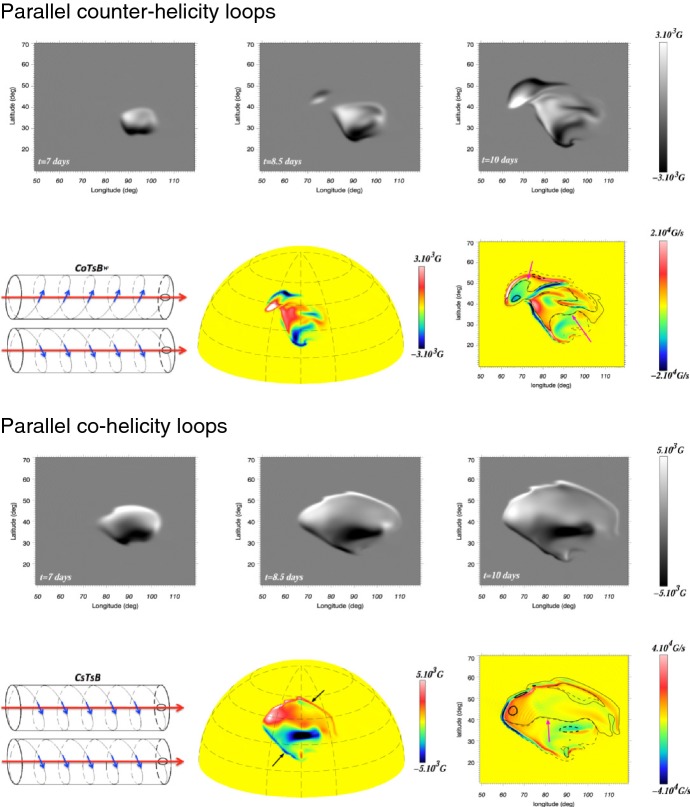
(Top) Simulation results of global-scale toroidal loops for the case with the same axial field but opposite handedness (RL0), which is illustrated as the cartoon. The panels in the first row and on the second middle indicate the radial magnetic field at the near-top layer at $0.93R_{\odot }$. The panel on the second right shows the radial current, on which the contours of the radial field at 80% (thick) and 20% (thin) of its maximum (solid) and minimum (dashed) are overplotted. The magenta arrows point to the PILs. Due to the bounce interaction of the emerging tubes, the surface magnetogram shows two emerging bipoles with different helicity signs. (Bottom) The same as the top panels but for the case with the same handedness and axial field (RR0). In this merging case, the emerging region consists of a large single bipole but shows a higher degree of non-neutralized currents. Image reproduced by permission from Jouve et al. (2018), copyright by AAS

The interaction of two emerging flux tubes inside the solar interior was also examined by Jouve et al. (2018) in a global scale. By extending their anelastic MHD models of the flux emergence in a spherical convective shell with large-scale mean flows (e.g., Jouve et al. 2013), they conducted simulations on the pairs of emerging toroidal loops that have different combinations of the twist handedness and axial direction. They found that if the two loops are given opposite handedness and the same axial direction or the same handedness but opposite axial direction, they bounce against each other through rising, which is in good agreement with RL0 and RR4 of Linton et al. (2001). Consequently, as in the top panels of Fig. 41, the map of the radial magnetic field near the top boundary (substituting the solar surface) shows a quadrupolar region constituted of two emerging bipoles. On the other hand, the case with parallel co-helicity loops (corresponding to RR0) yields a simple bipolar pattern due to the merging of the loops [Fig. 41(bottom)], just like the first model of Murray and Hood (2007). However, in such a case, the non-neutralized currents, suggested to be the origin of eruptive events (Sect. 3.2.5), are much more pronounced than the other cases because the return currents contained in the periphery of each loop are annihilated at the current sheet between the merging loops. From the series of simulation runs in Jouve et al. (2018), a variety of AR structures are formed by interaction of two rising flux tubes, from simple bipolar to complex quadrupolar ones. Since the magnetograms investigated in this study are at $0.93R_{\odot }$ (i.e., about 50 Mm below the actual surface of the Sun) due to the limitation of anelastic models, further investigations with the fully compressible calculations that enable the direct access to the surface are needed to elaborate how much of the emerging flux does reach the photosphere and what the possible AR configurations at the surface are.

ARs with much higher degree of complexity were modeled by Prior and MacTaggart (2016), who simulated the buoyant emergence of braided magnetic fields from the convection zone to the corona. For instance, their “pigtail” field, in which three flux tubes are entangled with each other, develops a magnetogram with a number of positive and negative polarities intertwined: see Fig. 13 of Prior and MacTaggart (2016).

#### Effect of turbulent convection

As we have discussed in Sect. 2.1 and above, thermal convection exerts a diverse range of impacts on the emerging flux, and the series of realistic simulations have revealed the dynamic interactions between the magnetic fields and convective flows, such as boost-up and pin-down of large-scale emerging fields (Fan et al. 2003; Jouve and Brun 2009), elongation of the surface granular cells (Martínez-Sykora et al. 2008; Cheung et al. 2008), and the local undulation of emerging fields (Tortosa-Andreu and Moreno-Insertis 2009; Fang et al. 2010; Cheung et al. 2010).

**Fig. 42:**
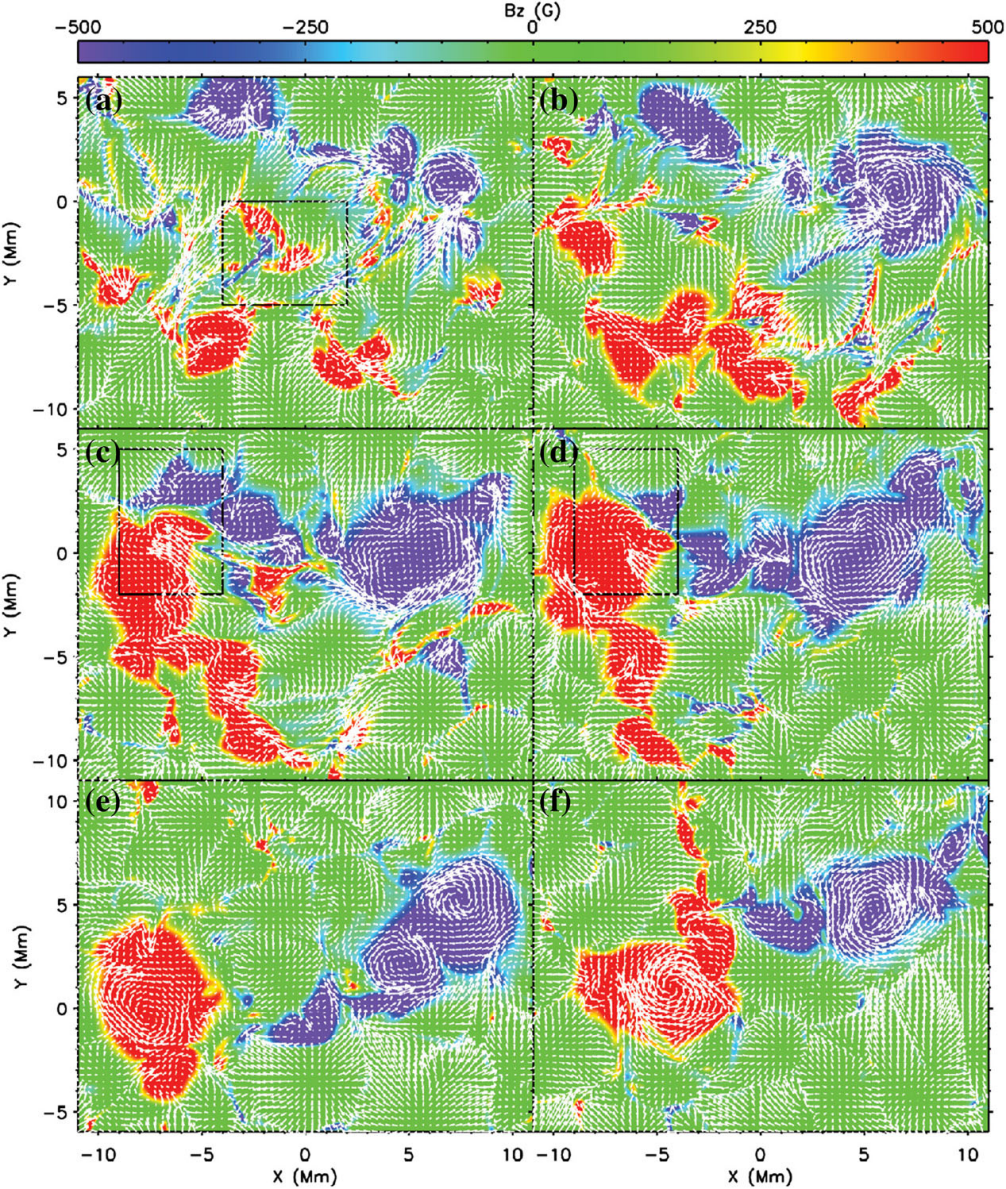
Temporal evolution of vertical magnetic field at the solar surface at **a** 3:45:00, **b** 4:15:00, **c** 5:10:00, **d** 5:35:00, **e** 6:23:00, and **f** 7:41:00 from the start of the simulation. Arrows show the horizontal velocity field. Noticeable shearing/converging flows are highlighted with the boxes. Image reproduced by permission from Fang et al. (2012b), copyright by AAS

**Fig. 43:**
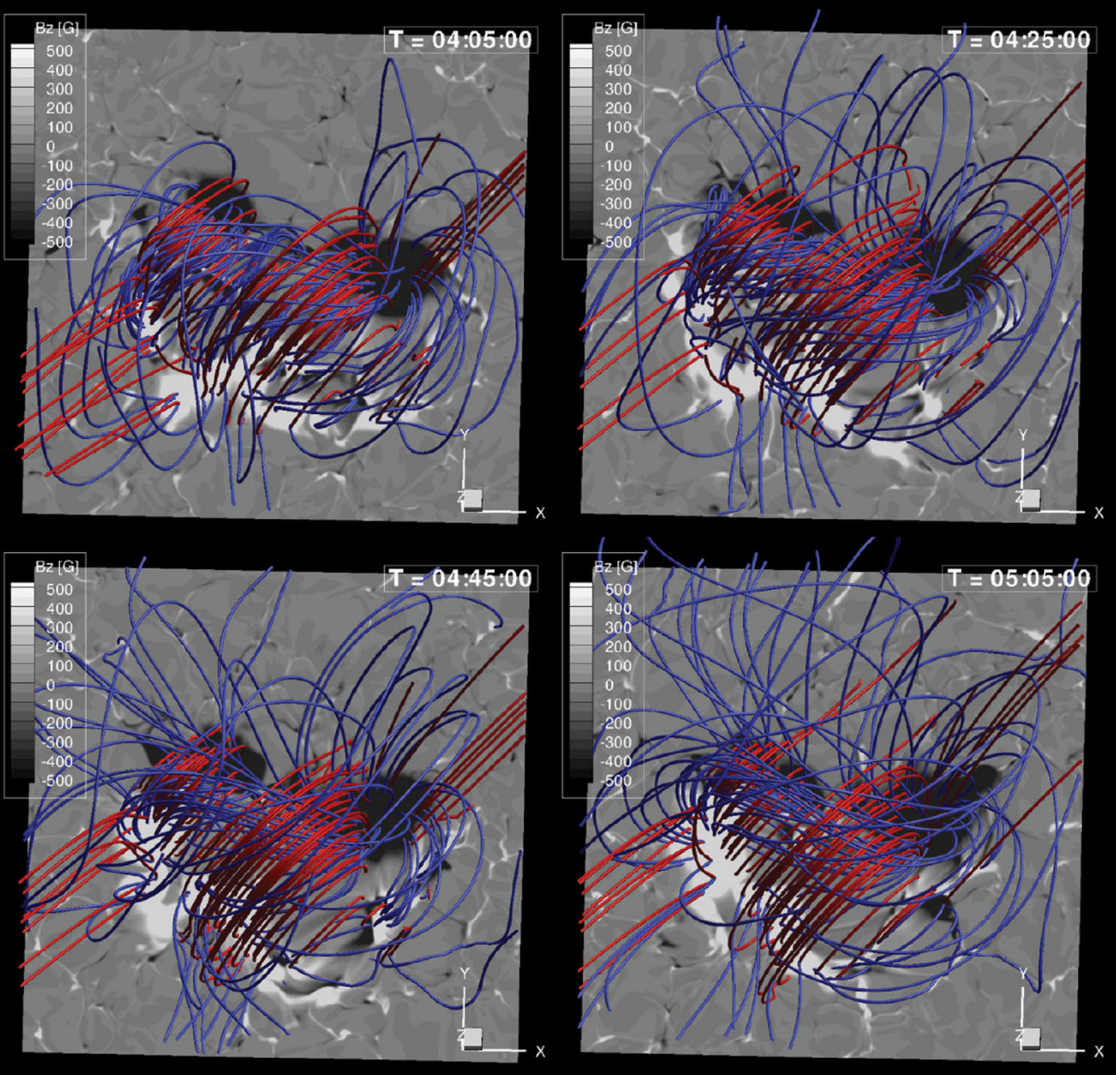
Comparison of the model field (blue) with the extrapolated potential field (red) at the times of 04:05:00, 04:25:00, 04:45:00, and 05:05:00 plotted on the photospheric magnetogram. The formation of non-potential sigmoidal field is clearly seen. Image reproduced by permission from Fang et al. (2012a), copyright by AAS

Fang et al. (2012a, b) simulated the buoyant rise of a twisted flux tube from the convection zone in which turbulent convection resides. Figure 42 shows the evolution of photospheric magnetograms, which reveals the rapid growth of magnetic concentrations (spots) with the unsigned total flux of up to $1.37\times 10^{21}\, \mathrm{Mx}$ (at $t=5$ h), the strong spot rotations (see the large negative spot at $x=6\, \mathrm{Mm}$), and the shearing and converging motions around the PIL. Here, both the shearing and rotational motions are driven by the Lorentz force and these motions transfer the magnetic energy and helicity into the corona (consistent with, e.g., Manchester 2001; Fan 2001b). The authors found that the convection-driven convergence flow produces a strong magnetic gradient and flux cancellation at the PIL. Together with the shear flow, the field lines above the PIL undergo a tether-cutting reconnection and produce long overlying sheared arcades and short submerging loops (Moore et al. 2001). Comparison of the model and extrapolated field lines in Fig. 43 clearly illustrates the development of non-potential, sigmoidal structure above the PIL that is covered by the more potential coronal loops.

Similar convective emergence simulation was also performed by Chatterjee et al. (2016), who employed a horizontal magnetic flux sheet instead of a tube at the start of the simulation. The flux sheet breaks up into several flux bundles due to the undular mode instability (Fan 2001a) and develops into a large-scale U-shaped loop, which appears in the photosphere as a pair of colliding flux concentrations (i.e., a $\delta$-spot). The strong cancellation between the two spots manifests as a series of flare eruptions with magnitudes comparable to GOES C- and B-class events (Korsós et al. 2018). Through the creation of a $\delta$-spot and the flaring activity, they observed the repeated formation of cool dense filaments above the PIL and the ejection of helical flux ropes.

Another intriguing possibility of $\delta$-spot formation was suggested by Mitra et al. (2014), who conducted the direct numerical simulation of the strong stratified dynamo with forced turbulence. Their 3D computation box holds two-layered turbulence, the helical and large-scale dynamo in the lower layer and the non-helical turbulence in the upper layer. As a result, they observed the formation of strong bipolar flux concentrations with super-equipartition fields, which sometimes move closer to take a $\delta$-spot configuration. While the large-scale magnetic field in the deeper layer is created through a large-scale dynamo ($\alpha$ effect), the spontaneous spot formation in the upper layer may be due to the so-called negative effective magnetic pressure instability (NEMPI), which is caused by suppression of the turbulent hydromagnetic pressure and tension due to the mean magnetic field (Brandenburg et al. 2011).

#### Toward the general picture

The numerical simulations introduced above have suggested the possibility that different types of flare-productive ARs have different subsurface origins and evolution histories (Zirin and Liggett 1987; Toriumi et al. 2017b). For example, the $\delta$-spots of Types 1 (Spot-spot) and 3 (Quadrupole) may be produced from the kinked and multi-buoyant-segment flux systems, respectively (Linton et al. 1999; Fan et al. 1999; Takasao et al. 2015; Toriumi et al. 2014b; Fang and Fan 2015).

**Fig. 44:**
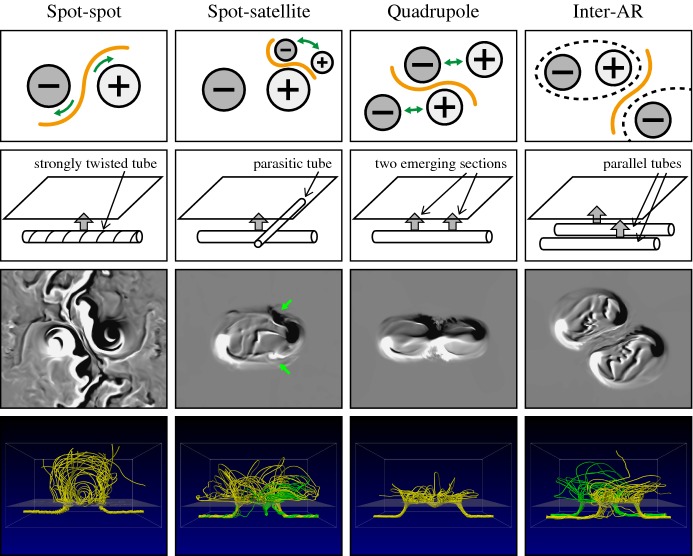
3D numerical simulations of the four representative types of flare-productive ARs, as introduced in Fig. 17. Images and movie reproduced by permission from Toriumi and Takasao (2017), copyright by AAS. (Top) Polarity distributions. (Second) Schematic diagrams showing the numerical setup. (Third) Surface vertical magnetic fields (magnetogram). The green arrows for the Spot-satellite case point to the satellite spots, which originate from the parasitic flux tube. (Bottom) Magnetic field lines. The green field lines are for the parasitic tube and the parallel tube. (For movie see Electronic Supplementary Material.) The accompanying movie shows the temporal evolutions for the four cases

In order to scrutinize the differences between the above three cases plus another type of X-flaring ARs, the Inter-AR case, created by two independent but closely neighboring episodes of flux emergence, Toriumi and Takasao (2017) conducted a systematic survey of flux emergence simulations by using similar numerical conditions with as little difference as possible, and explored the formation of $\delta$-spots, flaring PILs, and their evolution processes. Figure 44 summarizes the numerical conditions and results. For the Spot-spot case, the initial twist strength is intensified so as to exceed the critical value for the kink instability (Linton et al. 1996, see also Sect. 4.1.1). The Spot-satellite is modeled by introducing a parasitic flux tube above the main tube in a direction perpendicular to it, the situation similar to the interacting tube models in Sect. 4.1.3. The Spot-satellite may also be produced from a single bifurcating tube, which, however, was not considered for the sake of simplicity. The Quadrupole flux tube has two buoyant sections along the axis, resembling the simulations in Sect. 4.1.2. Finally, for the Inter-AR case, two flux tubes are placed in parallel.

As the movie of Fig. 44 demonstrates, all cases except for Inter-AR produce $\delta$-shaped polarities with strongly-sheared, strong-gradient PILs in their cores that are coupled with flow motions, but the most drastic evolution appears for the Spot-spot case. As discussed in Sect. 4.1.1, the knotted apex enhances the buoyancy that leads to the fastest emergence among the four cases. The total unsigned magnetic flux in the photosphere

17 $$\begin{aligned} \varPhi =\int _{z=0} |B_{z}|\, dS \end{aligned}$$

and the free magnetic energy stored in the atmosphere

18 $$\begin{aligned} \varDelta E_{\mathrm{mag}}\equiv E_{\mathrm{mag}}- E_{\mathrm{pot}} =\int _{z>0} \frac{{{\mathbf {B}}}^{2}}{8\pi }\, dV -\int _{z>0} \frac{{{\mathbf {B}}}_{\mathrm{pot}}^{2}}{8\pi }\, dV, \end{aligned}$$

where ${{\mathbf {B}}}_{\mathrm{pot}}$ is the potential field, are also largest for the Spot-spot case.

**Fig. 45:**
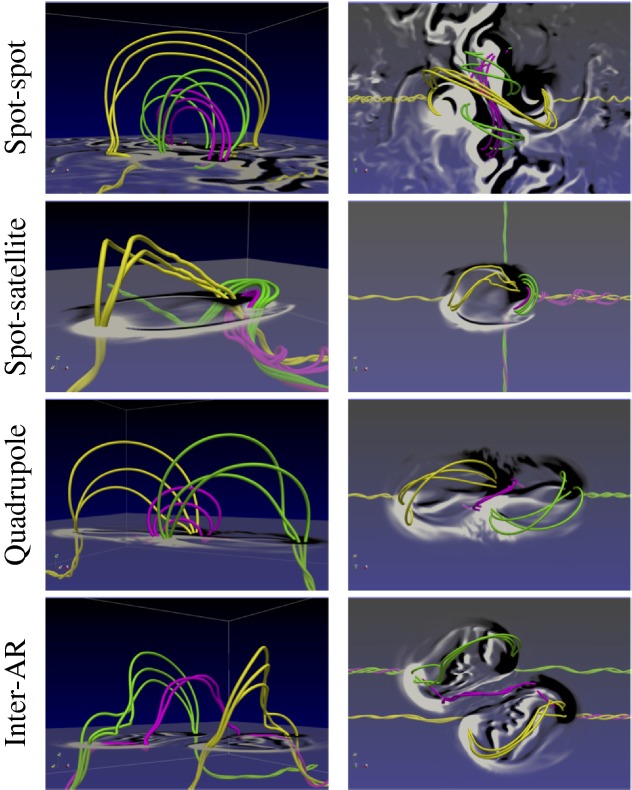
Modeled 3D magnetic structures for the four types of flare-producing ARs in Toriumi and Takasao (2017). The purple field lines are the newly formed flux rope structure, created through magnetic reconnection of emerged loops indicated with yellow and green lines. Except for the Spot-spot case, the flux ropes are exposed and have an access to the outer space. On the contrary, the Spot-spot flux rope is covered by the overlying arcade. Image reproduced by permission, copyright by AAS

It is also suggested from these models that the difference in initial simulation setup may determine the fate of a CME eruption. As shown in Fig. 45, in the case of Spot-satellite, Quadrupole, and Inter-AR, the newly formed flux rope above the sheared PIL is exposed to outer space, an ideal situation for successful CME eruption. However, in the Spot-spot case, the flux rope is trapped and confined by the overlying loops. Very strong confinement may explain the flare-rich but CME-poor nature of the Spot-spot AR NOAA 12192 (see Fig. 1 and discussion on successful and failed eruptions in Sect. 2.2).

In addition, this model is able to account for the formation of “magnetic channels,” another important feature of the flaring PILs (Zirin and Wang 1993a, see Sect. 3.2.1). In the magnetogram of the Spot-spot case (Fig. 44), one may find that the central PIL has an elongated alternating pattern of positive and negative polarities, resembling the magnetic channel. This structure is produced because the photospheric fields are highly inclined to horizontal and almost parallel to the PIL with slight undulations.

The series of simulations above provides a unified, general view of the birth of flare-productive ARs. Within the solar interior, probably due to convective evolution, the emerging flux systems that form $\delta$-spots are severely twisted to take on tortuous structures, partially pinned down to bear multiple rising segments, bifurcated into entangled branches, or hit against other flux systems to undergo mutual interactions. All of these processes are prone to enhancement of free magnetic energy. As the fluxes reach the photosphere, complex magnetic structures, prominently manifested by $\delta$-spots, sheared PILs, sheared coronal arcades, and flux ropes, develop. The $\delta$-spots are likely generated by multiple emerging loops instead of a single $\varOmega$-loop, and the different patterns of polarity layouts, such as Types 1, 2, and 3, stem from the difference in the subsurface evolution. Even two separated, seemingly independent ARs may intensify the free energy if located in the closer proximity (Inter-AR case). The stored free energy is, if accumulated enough, released in the form of flares and CMEs.

One possibility that was not considered in Toriumi and Takasao (2017) is the situation where a new, delayed flux emerges into a pre-existing flux system (i.e. the concepts of successive emergence, complexes of activities, and sunspot nests in Sect. 3.1). Schrijver (2007) interpreted the formations of flaring PILs with this idea and Welsch and Li (2008) overall agreed. This situation is qualitatively similar to the Spot-satellite case, in which a minor bipole appears in the close proximity to the major sunspot, but the scale is much larger. Therefore, toward a more complete view, we may need to take into account this successive emergence case.

### Flux cancellation models

It is thought that coronal flux ropes can also form post-emergence as a coronal response to photospheric driving. Antiochos et al. (1994) and DeVore and Antiochos (2000) demonstrated that a sheared arcade lying above a PIL, produced by shearing motion in the photosphere (without convergence), contains a dipped structure that supports the prominence material. In the theory of van Ballegooijen and Martens (1989) (see Fig. 26), coronal loops above the PIL become sheared and converged due to photospheric motions and eventually reconnect against each other to form a flux rope. Most of the simulations based on this theory, often referred to as the “flux cancellation” models, deal with the evolution of coronal field lines within the computational box above the photospheric surface, i.e., the situation after the magnetic flux is emerged.

**Fig. 46:**
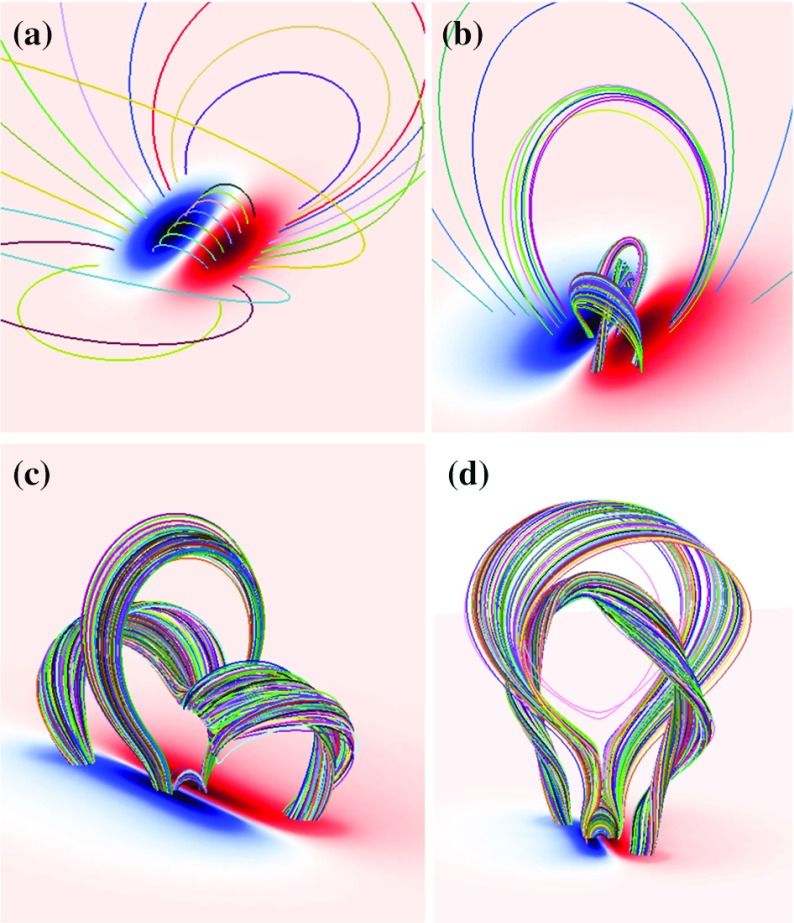
Flux cancellation model by Amari et al. (2003a). Image reproduced by permission, copyright by AAS. **a** Initial bipolar potential fields (i.e., $t=0$). A pair of counter-clockwise twisting motions is imposed at the bottom boundary from $t=0$ to $t_{\mathrm{s}}$, followed by a viscous relaxation from $t=t_{\mathrm{s}}$ to $t_{0}$. **b** Field lines of the magnetic configuration after the converging flow is applied from $t_{0}=400\tau _{\mathrm{A}}$ to $450\tau _{\mathrm{A}}$, where the unit $\tau _{\mathrm{A}}$ denotes the Alfvén transit time. Shown is the case for $t_{\mathrm{s}}=200\tau _{\mathrm{A}}$, in which the sheared loops are obvious around the PIL. **c** The state after the convergence is applied to $t=498\tau _{\mathrm{A}}$. A helical flux rope, low-lying arcade, and overlying arcade are now formed through magnetic reconnection between the sheared loops. **d** The convergence is further applied to $t=530\tau _{\mathrm{A}}$. The flux rope erupts upward with entraining the overlying arcades successively

Figure 46 shows the representative 3D calculation by Amari et al. (2003a). Here, the original potential field (panel a) is twisted by two co-rotating vortices imposed at the photospheric boundary. After the system is relaxed (panel b), converging motion is applied and magnetic reconnection between the sheared loops leads to the formation of a twisted flux rope, with a small low-lying arcade below, and an overlying arcade above (panel c). As the reconnection goes on, the unstable flux rope is ejected (panel d).

For instigating the flux cancellation of sheared loops, several types of mechanisms have been considered (see, e.g., Mackay et al. 2010; Aulanier 2014). Other than the convergence flow (Amari et al. 2003a; Aulanier et al. 2010), proposed mechanisms include decrease of photospheric flux through shearing motion (Amari et al. 2000, 2010), turbulent diffusion (Amari et al. 2003b; Mackay and van Ballegooijen 2006; Yeates and Mackay 2009; Aulanier et al. 2010), and reversal of magnetic shear (Kusano et al. 2004).

**Fig. 47:**
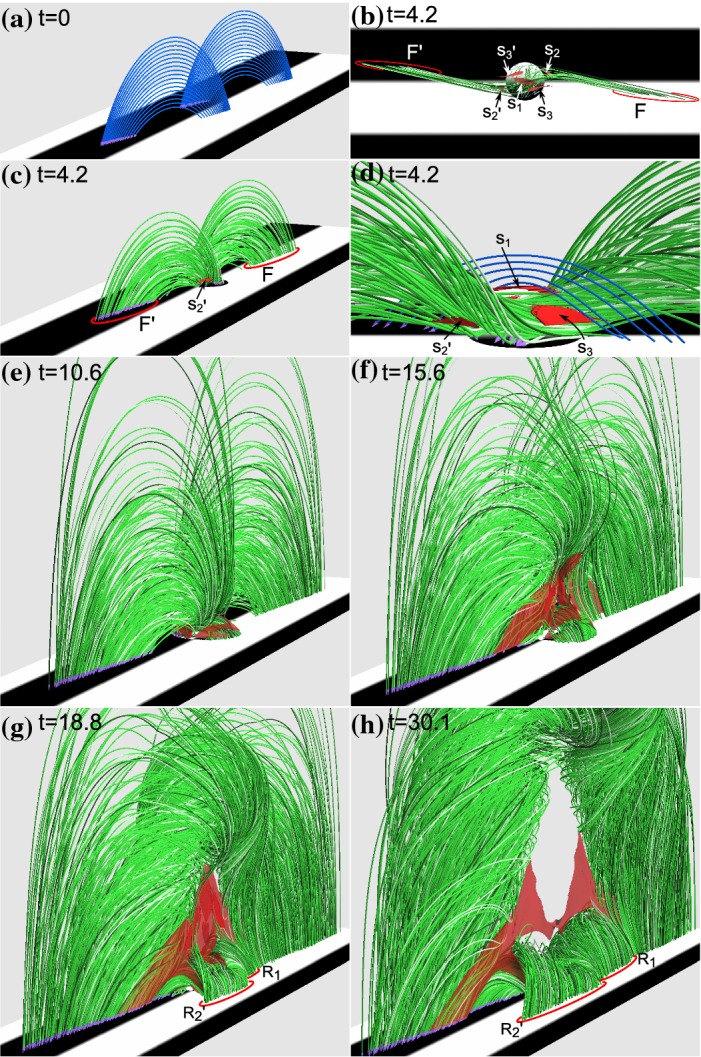
Flux rope formation and eruption by opposite-polarity type emerging flux. Image reproduced by permission from Kusano et al. (2012), copyright by AAS. Green tubes show the field lines with connectivity that differs from the initial state, while the blue tubes in panels **a** and **d** are the original sheared arcades. Gray scale at the bottom indicates the vertical field $B_{z}$ (white, positive; black, negative) and red contours denote the strong current layer. The initial sheared arcades (blue lines in panel **a**) go through reconnection triggered by the emerging flux at the bottom boundary and a helical flux rope is created (panels **b**–**d**). The flux rope is ejected leaving a current sheet underneath (panels **e**–**h**)

Kusano et al. (2012) investigated the process where the sheared arcade field above the PIL reconnects to create a flux rope and erupts, triggered by emerging flux from the photospheric surface (rather than the convergence flow or diffusion). This model sheds light on the importance of small-scale magnetic structures, which are often observed around flaring PILs, in the destabilization of the entire system (Toriumi et al. 2013a; Bamba et al. 2013; Wang et al. 2017b). In the particular simulation case of Fig. 47, emerging flux with the field direction opposite to that of the arcades triggers the reconnection and produces an erupting flux rope. From a systematic survey on the orientations of arcade and emerging flux, it was found that there exist two kinds of emerging flux capable of initiating the cancellation: the opposite-polarity type (shown as Fig. 47) and the reversed-shear type (comparable to Kusano et al. 2004).

As a more recent attempt, Xia et al. (2014), Xia and Keppens (2016) and Kaneko and Yokoyama (2017) performed 3D flux cancellation simulations that take into account the effect of thermodynamical processes. Due to the strong radiative cooling, coronal plasma within the helical field lines of the flux rope becomes condensed and piles up on the dipped part at the bottom. In this way, these authors successfully reproduced filaments (prominences) in a more realistic manner than those lacking in the thermodynamical processes.

### Data-constrained and data-driven models

#### Field extrapolation methods

One way to trace the development of coronal magnetic field is to sequentially compute the field lines from the routinely measured photospheric magnetograms by using extrapolation methods which neglect non-magnetic forces (such as pressure gradient) and assume that the Lorentz force vanishes, i.e., the force-free condition,

19 $$\begin{aligned} {\mathbf {j}}\times {{\mathbf {B}}}=0, \end{aligned}$$

where ${\mathbf {j}}$ is the current density

20 $$\begin{aligned} {\mathbf {j}}=\frac{c}{4\pi }{\mathbf {\nabla }}\times {{\mathbf {B}}}. \end{aligned}$$

The potential (current-free) field is the simplest approximation, under which $\nabla \times {{\mathbf {B}}}=0$. This can be replaced by

21 $$\begin{aligned} {{\mathbf {B}}}=-\nabla \psi , \end{aligned}$$

where $\psi$ is the scalar potential, and combined with the solenoidal condition ($\nabla \cdot {{\mathbf {B}}}=0$), further rewritten as

22 $$\begin{aligned} \nabla ^{2}\psi =0. \end{aligned}$$

The potential coronal field is calculated by solving this equation with using the normal component of the photospheric field $B_{z}$ as the boundary condition. Schrijver et al. (2005) and Schrijver (2016) assessed the non-potentiality of coronal fields of 95 and 41 ARs by comparing potential field extrapolations to the corresponding coronal images from the Transition Region and Coronal Explorer (TRACE; Handy et al. 1999) and SDO/AIA, respectively. They concluded that, in most cases, significant non-potentiality exists in ARs with newly emerging flux within ${\sim }\,30$ h or when opposite-polarity concentrations are evolving and in close contact.

The force-free condition, Eq. (19), is also expressed as

23 $$\begin{aligned} \nabla \times {{\mathbf {B}}}=\alpha {{\mathbf {B}}}, \end{aligned}$$

where $\alpha$ is called the force-free parameter. If $\alpha$ is constant everywhere in the coronal volume under consideration, the magnetic field is called a linear force-free field (LFFF); otherwise, a non-linear force-free field (NLFFF). In these models, all components of the vector magnetogram are used as the bottom boundary condition. As Figs. 26 and 31 show, the NLFFF extrapolations provide realistic coronal fields comparable to the actual observations. By applying NLFFF methods to the complex quadrupolar AR NOAA 11967, Liu et al. (2016b) and Kawabata et al. (2017) investigated the topology of coronal fields and elucidated the homologous occurrence of X-shaped flares. However, it has been shown that the NLFFF models are sensitive to the quality of photospheric boundary conditions, and thus do not faithfully reproduce observed coronal loop structures (e.g., DeRosa et al. 2009, 2015). Moreover, the input vector magnetograms are subject to the intrinsic ambiguity in the direction of the transverse magnetic field and this hampers fundamentally any magnetogram-driven coronal field reconstructions.

Representative NLFFF techniques include the optimization method, MHD relaxation method, and flux-rope insertion method. For the basis and comparison of various extrapolation methods, we refer the reader to DeRosa et al. (2009, 2015), Wiegelmann and Sakurai (2012) and Inoue (2016).

#### Data-constrained models

**Fig. 48:**
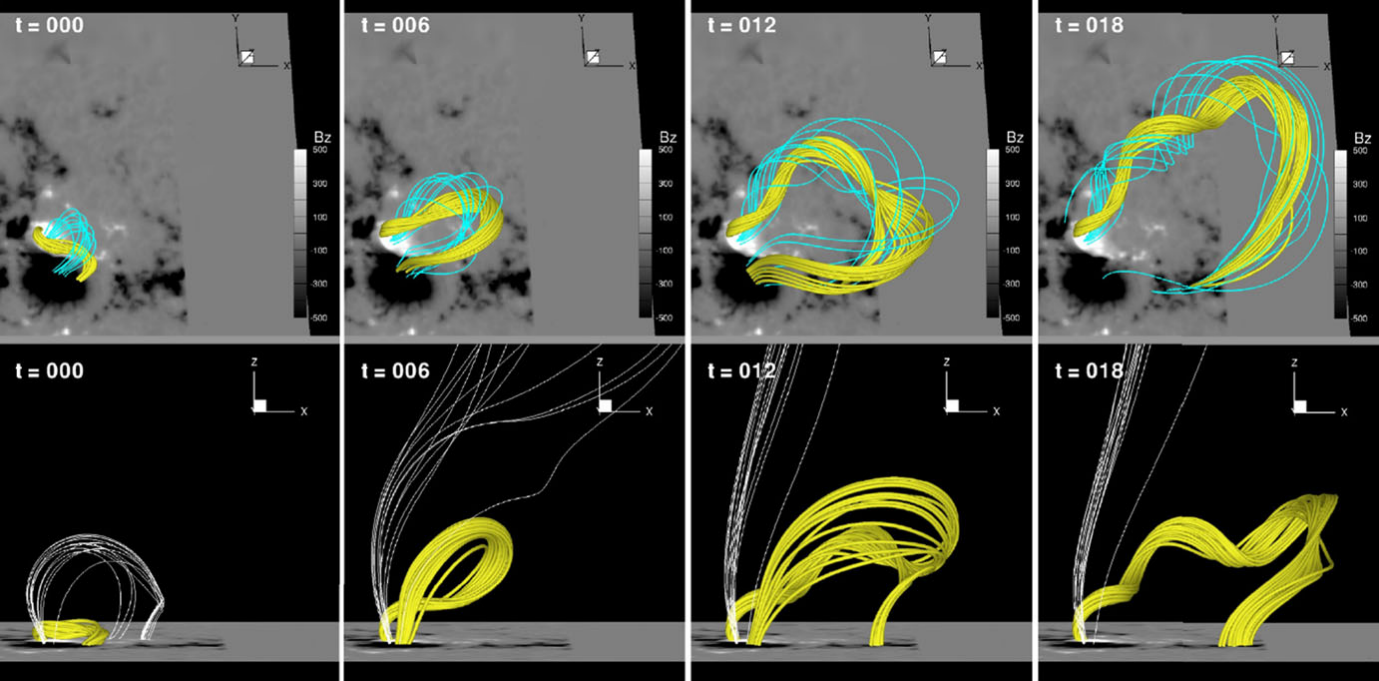
Data-constrained MHD simulation of the flux rope eruption in AR NOAA 11283. Yellow and cyan lines are the magnetic field lines traced from the same positive polarity. Another set of field lines (white) are those that pass through the null point, and reconnect and open. Bottom boundary is the photospheric magnetogram. The sigmoidal flux rope (yellow field lines at $t=0$, reproduced with NLFFF) becomes unstable and launched. Image reproduced by permission from Jiang et al. (2013), copyright by AAS

Even if one applies the most sophisticated technique of the NLFFF extrapolations to the accurate sequential magnetograms by Hinode/SOT and SDO/HMI, the obtained temporal evolution is still far from the real one because these models unavoidably assume a static state. One approach to overcome this issue is to use time-evolving data-constrained modeling. In this more physics-based method, the temporal evolution is obtained by solving the MHD equations with setting the reconstructed coronal field for the initial condition. Jiang et al. (2013) were the first to apply this method to the actual AR. As in Fig. 48, they reconstructed the initial coronal field of AR NOAA 11283 with the NLFFF model and demonstrated the CME eruption from this AR. According to the authors, due to small numerical errors in the extrapolation (i.e., their NLFFF was not perfectly force free), the system became unstable and the flux rope was erupted via the torus instability.

**Fig. 49:**
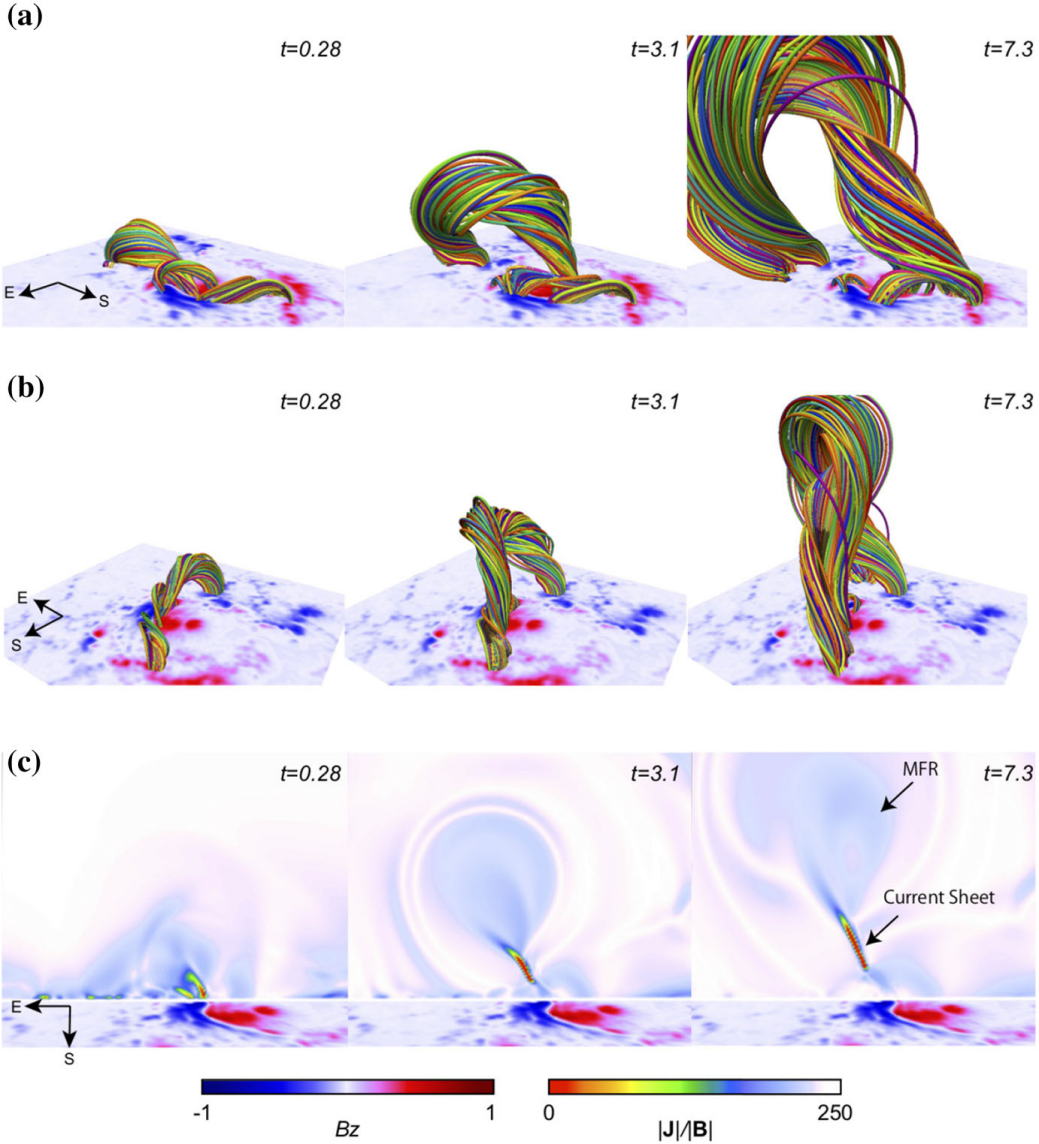
The formation and evolution of an eruptive flux rope in the X9.3-class flare in AR NOAA 12673. The top and second rows provide the field lines and magnetogram ($B_{z}$) that are viewed from two different angles and the bottom row shows the distribution of electric current in a vertical cross-section. In this model, multiple flux ropes along the PIL at the initial stage ($t=0.28$) reconnect and merge into a single flux rope ($t=3.1$), which eventually erupts into the higher atmosphere ($t=7.3$). Image reproduced by permission from Inoue et al. (2018b), copyright by AAS

Since then, the data-constrained approach has become the hot topic (Kliem et al. 2013; Amari et al. 2014). Inoue et al. (2014, 2015) modeled the X2.2-class event in NOAA 11158 (Fig. 14) and found that, interestingly, the flux rope at the core of this AR does not erupt directly but rather reconnects with ambient weakly twisted fields. Then, the ambient field transforms into a flux rope, which eventually exceeds the critical height of the torus instability. Muhamad et al. (2017) applied this method to NOAA 10930 (e.g., Figs. 6 and 19) and, by inserting emerging flux at the PIL from the bottom boundary, they succeeded in triggering the flux rope eruption, which is in line with the flare-triggering scenario by Kusano et al. (2012). The dramatic eruption in the X9.3 flare in NOAA 12673, which we introduced in Sect. 3.4, was modeled by Inoue et al. (2018b). They found that, as in Fig. 49, multiple compact flux ropes lying along the sheared PIL reconnect with each other and merge into a large, highly twisted flux rope that eventually erupts.

#### Data-driven models

Even more realistic reconstruction of the evolving coronal field is to sequentially update the photospheric boundary condition, which is called the data-driven model. The first approach of the data-driven models we show here is the magneto-frictional method (Yang et al. 1986), in which the magnetic field evolves due to the Lorentz force,

24 $$\begin{aligned} {{\mathbf {v}}}=\frac{1}{\nu c}\, {\mathbf {j}}\times {{\mathbf {B}}}, \end{aligned}$$

where $\nu$ is the frictional coefficient. In this formulation, the (pseudo) velocity is simply proportional to the Lorentz force. Cheung and DeRosa (2012) applied this method to the sequential magnetogram of NOAA 11158 and reproduced flux ropes that were ejected in the series of M- and X-class flares in this AR.

Another recent, yet nascent attempt is to directly solve the MHD equations with sequentially replacing the magnetogram to self-consistently reconstruct the coronal evolution (Wu et al. 2006). This was demonstrated by Jiang et al. (2016a, b) for ARs NOAA 11283 and 12192, respectively. Hayashi et al. (2018) calculated the photospheric electric field ${\mathbf {E}}$ from the sequential magnetogram ${{\mathbf {B}}}$ and drove the model of NOAA 11158 through Faraday’s law

25 $$\begin{aligned} \frac{\partial {{\mathbf {B}}}}{\partial t}=-c\nabla \times {\mathbf {E}}, \end{aligned}$$

instead of solving the induction equation

26 $$\begin{aligned} \frac{\partial {{\mathbf {B}}}}{\partial t}=\nabla \times ({{\mathbf {v}}}\times {{\mathbf {B}}}). \end{aligned}$$

**Fig. 50:**
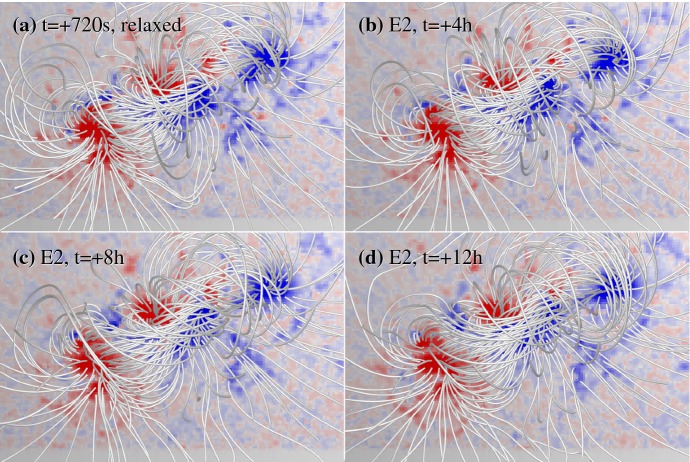
Data-driven model of NOAA 11158, performed with a time-evolving photospheric electric field. The initial relaxed coronal field (**a**) is stretched and sheared over time especially above the central PIL. Image reproduced by permission from Hayashi et al. (2018), copyright by AAS

Here, ${\mathbf {E}}$ is determined, for instance, by solving Ohm’s law (${\mathbf {E}}=-{{\mathbf {v}}}\times {{\mathbf {B}}}/c$) by using the velocity ${{\mathbf {v}}}$ obtained with flow tracking techniques (see Welsch et al. 2007, and references therein). As Fig. 50 displays, the initial coronal field, obtained by matching the potential field to the observed vector magnetogram and relaxing it, undergoes substantial elongation and twisting, especially above the central PIL, in response to the shear motion in the photosphere.

A data-driven, dynamic model is supposed to calculate the coronal field that matches the changing photospheric magnetogram. An accurate model would, in principle, produce a flare or eruption at the same time that the actual Sun does. Inevitable simplifications of the model and inaccuracies in its initial state, however, suggest that it may be difficult to reproduce flares or eruptions. This is because the observed, gradual photospheric change (before and around the flare onset) might be insufficient to cause any drastic change in the (inaccurate) model’s coronal field.

Another caveat is that the model is limited by the temporal frequency of the driving data. Using the flux emergence simulation as the ground-truth data set, Leake et al. (2017) performed a data-driven simulation with the assumption that the photospheric information is provided every 12 min (the default cadence of the SDO/HMI vector magnetogram). They showed that the data-driven models can reproduce the slowly emerging ARs over 25 h with only ${\sim }\, 1$% error in the free magnetic energy. However, the modeling was largely affected by rapidly evolving features. Even if one applies interpolation to the driving data, the coarse sampling generates a strobe effect, in which smoothly evolving features appear to jump across the photosphere. For an emerging bipole with a spatial extent of $L=1\, \mathrm{Mm}$ with an apparent horizontal velocity of $v_{\mathrm{h}}=20\, \mathrm{km\ s}^{-1}$, the sampling interval needs to be less than $L/v_{\mathrm{h}}=50\, \mathrm{s}$. Note that this may be partly overcome by using faster-cadence LOS magnetograms.

### Summary of this section

In this section, we presented theoretical investigations that try to address the subsurface origin and physical mechanisms behind the large-scale/long-term evolution of flare-producing ARs. We first showed in the beginning of Sect. 4.1 that classical flux emergence simulations of the $\varOmega$-loop emergence can explain several characteristics, such as magnetic tongues, formation of flux ropes and sigmoids, generation of shear flows and spot rotation, helicity injection, and non-neutralized currents. However, most of these models do not reproduce other important features of flaring ARs such as the highly-sheared PIL between closely neighboring opposite-polarity sunspots.

From the observational evidence of emergence of top-curled flux tube, the helical kink instability was invoked as the possible production mechanism of the $\delta$-sunspots (Sect. 4.1.1). 3D models demonstrate that (1) a tightly twisted tube develops a kink instability; (2) the rise speed of the kinked tube is accelerated due to the enhanced buoyancy; and (3) the tube reproduces a quadrupolar polarity pattern with a sheared PIL on the photospheric surface. These models can reproduce the observed characteristics of Type 1 (Spot-spot) $\delta$-spots.

Type 3 (Quadrupole) $\delta$-spots may be produced by the emergence of a flux tube with multiple buoyant segments (Sect. 4.1.2). Such a top-dent configuration is in fact created in a large-scale convective emergence model. Inspired by the observation of the quadrupolar AR NOAA 11158, the emergence of a flux tube that rises at two sections along the axis was investigated. It was found that the time evolution of the photospheric polarities, i.e., the collision, shearing, and converging motions of the central bipole, is fairly consistent with that of the actual AR. Such evolutions were not achieved by a pair of emerging flux tubes that are placed in parallel. Together with the follow-up study, the multi-buoyant segment model is considered as a likely candidate for quadrupolar $\delta$-spots.

Interaction of emerging flux systems is also recognized as a source of complexity (Sect. 4.1.3). In fact, 3D simulations showed that complex-shaped ARs can be created by interaction of multiple tubes in the solar interior. One interesting consequence of the interaction, both aerodynamic and bodily, is that even simple bipolar ARs may originate from multiple flux systems through merging. In this case, non-neutralized currents can be significant because the return currents are annihilated.

Turbulent convection results in a multitude of effects on the rising flux (Sect. 4.1.4). The convective emergence simulation revealed that the two polarities on the photosphere undergo shearing and rotational motion due to the Lorentz force and that the converging motion at the PIL causes flux cancellation, which leads to the production of a flux rope in the atmosphere. It was also found that the strong collision of opposite polarities results in a series of flare eruptions.

With the aim to obtain a unified perspective of production of flaring ARs, a comparison of different modeling setups was performed (Sect. 4.1.5). It was assumed that the production of Spot-spot, Spot-satellite, Quadrupole, and Inter-AR types are due to the emergence of a kink-unstable tube, two interacting tubes, a multi-buoyant-segment tube, and two independent tubes, respectively. Although all models except for the Inter-AR case successfully reproduced $\delta$-spots with flaring PILs, the Spot-spot case showed a by far fastest rising with the largest free magnetic energy. Therefore, the difference in the observed evolution on the solar surface likely stems from the subsurface history, probably caused by turbulent convection, such as a strong twisting, downward pinning, and collision with other flux systems.

Flux rope formation and the consequent eruption have been extensively surveyed in the sheared arcade and flux cancellation models (Sect. 4.2). Many of these simulation models are based on the filament formation theory by van Ballegooijen and Martens (1989): the coronal fields are tied to the photospheric bottom boundary and the photospheric motion, such as shearing, converging, and/or diffusion, drives the overall evolution. However, the reversed-shear and small-scale emerging field at the PIL are also suggested as the trigger of magnetic reconnection between coronal arcades. Flux cancellation models that take into account the effect of thermodynamics now reproduce the condensation of filament plasma due to radiative cooling.

Along with the extrapolation methods (Sect. 4.3.1), recent progress in the more physics-based modeling of the coronal field is facilitated by the development in magnetographs, especially by the advanced vector magnetograms of Hinode/SOT and SDO/HMI. There are two methods in this category, which are data-constrained models, where a single snapshot is used for creating the initial coronal field (Sect. 4.3.2), and data-driven models, where the bottom boundary is sequentially updated to drive the calculation (Sect. 4.3.3). These methods, although still in the stage of development, provide the means to trace the evolution of coronal fields in a more realistic manner, such as the formation of flux ropes in response to the photospheric motion and the resultant eruptions, and may open the door to real-time space weather forecasting.

## Rapid changes of magnetic fields associated with flares

As we saw in the previous sections, the gradual magnetic field evolution (in the time scale of hours to days) is the key factor for the energy build up of solar eruptions. Then, can solar eruptions in the corona cause rapid (within minutes) magnetic field changes in the photosphere? The changes in the photosphere in response to the coronal eruptions have been expected to be small because the photospheric plasma density is much larger than that of the corona. Aulanier (2016) gave a review of this topic from both observational and modeling perspectives and provided a physical analysis of this issue called the “tail wags the dog” problem. Under certain circumstances, the coronal eruption can cause rapid changes in the photospheric magnetic topology.

Earlier, Hudson et al. (2008) and Fisher et al. (2012) quantitatively assessed the back reaction on the solar surface and interior resulting from the coronal field evolution required to release energy and made the prediction that after flares, the photospheric magnetic field would become more horizontal at the flaring PILs. Their analysis is based on the principle of energy and momentum conservation and builds upon the proposal by Hudson (2000) that the coronal field should, in an overall sense, contract or implode if there is a net decrease in magnetic energy (coronal implosion). This is one of the very few models that specifically predict that magnetic destabilization associated with flares can be accompanied by rapid and permanent changes of photospheric magnetic fields and the pattern of the field changes. One special case related to this scenario is the tether-cutting reconnection model for sigmoids (Moore et al. 2001; Moore and Sterling 2006), which involves a two-stage reconnection process. At the eruption onset, the near-surface reconnection between the two sigmoid elbows produces a low-lying shorter loop across the PIL and a larger twisted flux rope connecting the two far ends of the sigmoid. The second stage reconnection occurs when the large-scale loop cuts through the arcade fields, which causes the erupting flux rope to evolve into a CME and precipitation of electrons to produce flare ribbons (see Fig. 7a for illustration). If scrutinizing the magnetic topology close to the surface, one would find a permanent change of magnetic fields that conforms to the scenario as described above: the magnetic fields turn more horizontal near the flaring PIL due to the newly formed short loops there.

Whereas an earlier review by Wang and Liu (2015) summarizes certain aspects of research up to that time, focusing primarily on the results obtained before the SDO era, this section summarizes more recent observational findings of rapid magnetic field and sunspot structure changes associated with flares and briefly discusses the related theoretical insights.

### Magnetic transients

**Fig. 51:**
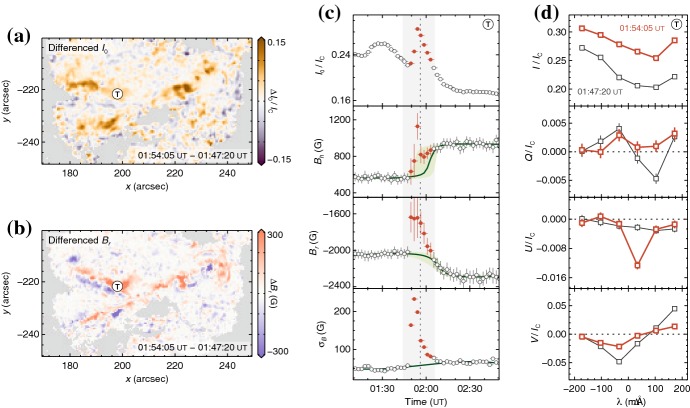
Flare-induced artifact as “magnetic transient.” **a** Differenced map of intensitygram. Symbol “T” marks the sample pixel. **b** Differenced map of magnetic field in the radial direction $B_{r}$. **c** Temporal evolution of the sample pixel. Red symbols show the frames affected by flare emission. Green curves show the fitted step-like function for the horizontal field $B_{h}$ and the radial field $B_{r}$ and a fitted third-order polynomial for the formal uncertainty of field strength ${\sigma }_{B}$; green bands show the $1\sigma$ fitting confidence interval. **d** Stokes profiles of the sample pixel at two instances, near (red) and before (gray) the flare peak. Image reproduced by permission from Sun et al. (2017), copyright by AAS

Before the discovery of the persistent photospheric magnetic field changes associated with flares, some studies showed observations of the so-called “magnetic transients”–the rapid, but short-lived change in the LOS magnetic fields. In the earlier studies (e.g., Tanaka 1978; Patterson 1984), these apparent transient reversals of magnetic polarity associated with flare footpoint emissions were interpreted as real physical effects of change in magnetic topology. Some later studies demonstrated that the short-lived magnetic transients are the observational effect due to changes in profiles of observing spectral lines caused by the flare emissions (Kosovichev and Zharkova 2001; Qiu and Gary 2003; Zhao et al. 2009), so they are sometimes called magnetic anomalies. The most comprehensive study in this topic is a recent paper by Sun et al. (2017), who analyzed the 135-s cadence HMI data and demonstrated the line profile changes and associated field signatures of transients (Fig. 51). Non-LTE^9^ modeling by Hong et al. (2018) explained the profile changes of Fe I 6173 Å line that the HMI uses and provided a quantitative assessment of magnetic transients. Song et al. (2018) suggested that magnetic transients and white-light flares are closely related spatially and temporally.

**Fig. 52:**
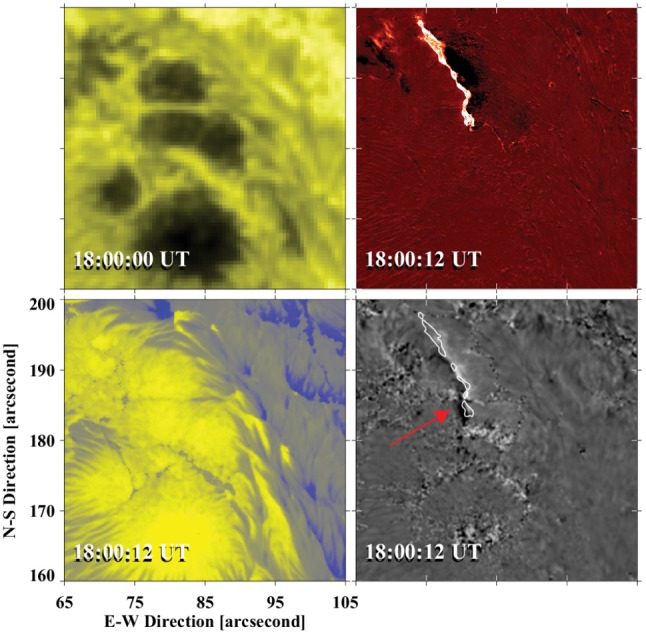
Azimuth angle changes in association with flare emission of 2015 June 22. The FOV is $40'' \times 40''$. (Top left) SDO/HMI white-light map. (Top right) Running difference image in H$\alpha$ blue wing (line core $-1.0$ Å), showing the eastern flare ribbon. The bright part is the leading front and the dark part is the following component. (Bottom left) The GST/NIRIS LOS magnetogram, scaled in a range of $-\,2500\, \mathrm{G}$ (blue) to $2500\, \mathrm{G}$ (yellow). (Bottom right) Running difference map of azimuth angle generated by subtracting the map taken at 17:58:45 UT from the one taken at 18:00:12 UT. The dark signal pointed by the pink arrow represents the sudden, transient increase of azimuth angle at 18:00:12 UT. Image reproduced by permission from Xu et al. (2018), copyright by the authors

All the above magnetic transients are for the LOS component of the magnetic fields. Taking advantage of the unprecedented resolution provided by the 1.6-m GST at BBSO, Xu et al. (2018) showed a sudden rotation of the magnetic field vector by about 12$^{\circ }$–20$^{\circ }$ counterclockwise, in association with the M6.5-class flare on June 22, 2015. Such changes of the azimuth angles of the transverse magnetic field are well pronounced within a ribbon-like structure (${\sim }\, 600\, \mathrm{km}$ in width), moving co-spatially and co-temporally with the flare emission as seen in the H$\alpha$ line (see Fig. 52). However, they are not related to the magnetic transients as shown above. A strong spatial correlation between the azimuth transient and the ribbon front indicates that the energetic electron beams are very likely the cause of the rotation. During the rotation, the measured azimuth becomes closer to that of the potential field, which indicates the process of energy release (untwisting motion) in the associated flare loop. The magnetic fields restored their original direction after the flare ribbons swept through over the area. This was the first time that a transient field rotation was observed. Possible explanations of this phenomenon include (1) effect of induced magnetic fields; (2) effect of downward-drafting plasma; (3) polarization of emission lines due to return current and/or filamentary chromospheric evaporation (different from the original concept of magnetic transient); and (4) effect of Alfvén waves. The authors claimed that the observed field change cannot be explained by existing models. This new, transient magnetic signature in the photosphere may offer a new diagnostic tool for future modeling of magnetic reconnection and the resulting energy release.

### Rapid, persistent magnetic field changes

In the early 1990s, the Caltech solar group discovered obvious rapid and permanent changes of vector magnetic fields associated with the flares using the BBSO data (Wang 1992; Wang et al. 1994a). They found that the transverse field shows much more prominent changes compared to the LOS component. Some of the results appeared to be puzzling: the magnetic shear angle (an indicator of non-potentiality), defined as the angular difference between the potential magnetic field and the measured field (see Sect. 3.2.1), increases following flares. It is well known that, in order to release the energy for a flare to occur, the coronal magnetic field has to evolve to a more relaxed state to release energy. For this reason, there have been some doubts to these earlier measurements, especially because the data were obtained from ground-based observatories that may suffer from certain effects such as atmospheric seeing and lack of continuous observing coverage.

Kosovichev and Zharkova (2001) studied high-resolution SOHO/MDI magnetogram data for the “Bastille Day Flare” on 2000 July 14, and found regions with a permanent decrease of magnetic flux, which are related to the release of magnetic energy. Using high cadence GONG data, Sudol and Harvey (2005) found solid evidence of step-wise field changes associated with a number of flares. The time scale of the changes is as fast as 10 min (GONG cadence is 1 min), and magnitude of change is in the order of 100 G. Petrie and Sudol (2010), Johnstone et al. (2012), Cliver et al. (2012) and Burtseva and Petrie (2013) also surveyed more comprehensively the rapid and permanent changes of LOS magnetic fields with GONG data, which were indeed associated with almost all the X-class flares studied by them.

The above studies using the LOS field data demonstrated the step-wise property of flare-related photospheric magnetic field change. However, the underlying cause of those changes was not clearly revealed. The work by Cameron and Sammis (1999) was the first to use near-limb magnetograph observations to characterize flare-related changes of magnetic fields, taking advantage of the projection effect. In a number of papers, it was found that, for the LOS magnetic field, the limb-ward flux increases in general, while the disk-ward flux in the flaring ARs decreases (Wang et al. 2002b; Wang 2006; Yurchyshyn et al. 2004; Spirock et al. 2002; Wang and Liu 2010). Such a behavior suggests that after flares, the overall magnetic field structure of ARs may change from a more vertical to a more horizontal configuration, which is consistent with the scenario that the Lorentz force change pushes down the field lines. Note that most of the observations listed in Wang and Liu (2010) are made by SOHO/MDI, which has a cadence of up to 1 min. The drastic change in inclination angle of magnetic fields in sunspots associated with the flare eruption was also detected by Ye et al. (2016) by using vector magnetograms from the SDO/HMI, and the observational result was consistent with the expectation of the coronal implosion scenario.

**Fig. 53:**
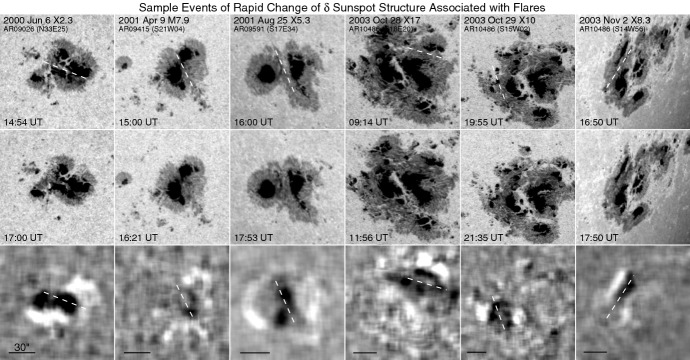
TRACE white-light images covering associated with six major flares. The rapid changes of $\delta$-sunspot structures are observed. The top, middle, and bottom rows show the pre-flare, the post-flare, and the difference images between them after some smoothing, respectively. The white pattern in the difference image indicates the region of penumbral decay, while the dark pattern indicates the region of darkening of penumbra. The white dashed line denotes the flaring PIL and the black line represents a spatial scale of 30”. Image reproduced by permission from Liu et al. (2005), copyright by AAS

**Fig. 54:**
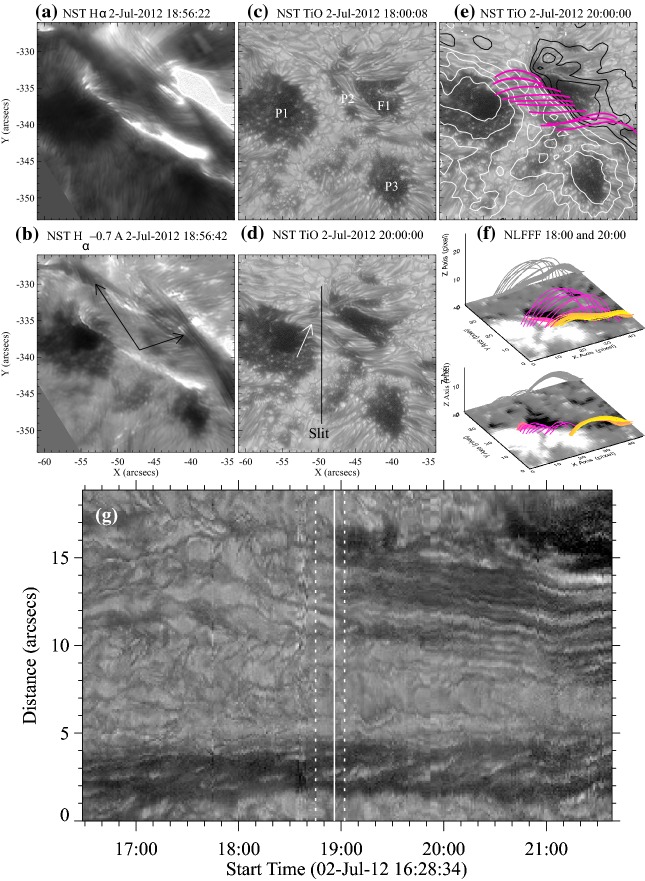
BBSO/GST H$\alpha$ center (**a**) and blue-wing (**b**) images at the peak of the 2011 July 2 C7.4 flare, showing the flare ribbons and possible signatures of a flux rope eruption (the arrows in panel **b**). The GST TiO images about 1 h before (**c**) and 1 h after (**d**) the flare clearly show the formation of penumbra (pointed to by the arrow in panel **d**). The same post-flare TiO image in panel **e** is superimposed with positive (white) and negative (black) HMI LOS field contours, and NLFFF lines (pink). **f** Perspective views of the pre- and post-flare 3D magnetic structures including the core field (a flux rope) and the arcade field from NLFFF extrapolations. The collapse of arcade fields is obvious. **g** TiO time slices for a slit (black line in panel **d**) across the newly formed penumbra area. The dashed and solid lines denote the time of the start, peak, and end of the flare in GOES 1–8 Å. The sudden turning off of the convection associated with the flare is obviously shown. Images reproduced by permission from Wang et al. (2013) and Jing et al. (2014), copyright by AAS. (For movie see Electronic Supplementary Material)

As more and more evidence indicates the irreversible photospheric magnetic field changes following flares, it is natural to find whether these changes are detectable in white-light structures of ARs. The white-light signatures of topological changes are indeed discovered in a number of papers (e.g. Wang et al. 2004a; Liu et al. 2005; Deng et al. 2005; Li et al. 2009; Wang et al. 2009, 2013, 2018b). The most prominent changes are the enhancement (i.e., darkening) of penumbral structure near the flaring PILs and the decay of penumbral structure in the peripheral sides (outer edges) of $\delta$-spots. Figure 53 clearly demonstrates some examples of such spot structure changes. The difference image between pre- and post-flare states always shows a dark patch at the flaring PIL that is surrounded by a bright ring. They correspond to the enhancement of the central sunspot penumbrae and the decay of the peripheral penumbrae, respectively. These examples were discussed in detail by Liu et al. (2005), in which they showed that (1) these rapid changes are associated with flares and are permanent, and (2) the decay of sunspot penumbrae is related to the magnetic field in the outer edge of AR that turns to a more vertical direction, while the darkening of sunspot structure near the central PIL is related to the magnetic field that turns to a more horizontal direction. Chen et al. (2007) statistically studied over 400 events using TRACE white-light data and found that the significance of sunspot structure change is positively correlated with the magnitude of flares. Using Hinode/SOT G-band data, Wang et al. (2012a) further studied the intrinsic linkage of penumbral decay to magnetic field changes. They took advantage of the high spatio-temporal resolution Hinode/SOT data and observed that in sections of peripheral penumbrae swept by flare ribbons, the dark fibrils completely disappear while the bright grains evolve into faculae where the magnetic flux becomes even more vertical. These results again suggest that the component of horizontal magnetic field of the penumbra is straightened upward (i.e., turning from horizontal to vertical) due to magnetic field restructuring associated with flares. Also notably, the flare-related enhancement of penumbral structure near central flaring PILs has also been unambiguously observed with BBSO/GST. Using GST TiO images with unprecedented spatial (0.1”) and temporal (15 s) resolution, Wang et al. (2013) reported on a rapid formation of sunspot penumbra at the PIL associated with the 2012 July 2 C7.4 flare (see Fig. 54 and the corresponding movie). The most striking observation is that the solar granulation evolves to the typical pattern of penumbra consisting of alternating dark and bright fibrils. Interestingly, a new $\delta$-sunspot is created by the appearance of such a penumbral feature, and this penumbral formation also corresponds to the enhancement of the horizontal field. Similar pattern of penumbral formation is shown by Wang et al. (2018b).

**Fig. 55:**
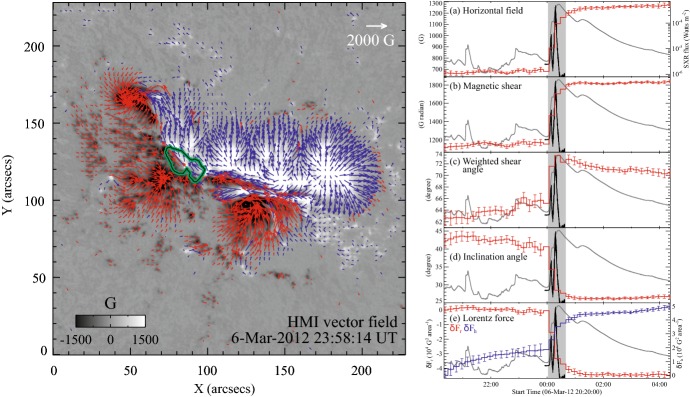
(Left) HMI vector magnetogram on 2012 March 7 showing the flare-productive AR NOAA 11429 right before the X5.4 flare. (Right) Temporal evolution of various magnetic properties of a compact region (green contour in the left panel) at the central PIL, in comparison with the light curves of GOES 1–8 Å soft X-ray flux (gray) and its derivative (black). Note that in panel **d**, the inclination is measured from horizontal direction. The shaded interval denotes the flare period in the GOES flux. Image reproduced by permission from Wang et al. (2012c), copyright by AAS

A very clear demonstration of flare related changes in vector magnetic fields came from the analysis of SDO/HMI vector data by Wang et al. (2012b). The analysis of the X2.2 flare in AR NOAA 11158 on 2011 February 15 clearly demonstrated a rapid/irreversible increase of the horizontal magnetic field at the flaring PIL. The mean horizontal fields increased by about 500 G within 30 min after the flare. The authors also found that the photospheric field near the flaring PIL became more sheared and more inclined towards horizontal, consistent with the earlier results (e.g., Wang 1992; Wang et al. 1994a; Liu et al. 2005). Following that initial study, a number of papers using HMI data demonstrated the consistent changes of magnetic fields (Liu et al. 2012; Sun et al. 2012; Wang et al. 2012c; Petrie 2012, 2013; Yang et al. 2014; Castellanos Durán et al. 2018). The found patterns of the changes are consistent in the sense that the transverse field enhances in a region across the central flaring PIL. Figure 55 shows the typical time profiles of such field changes.

**Fig. 56:**
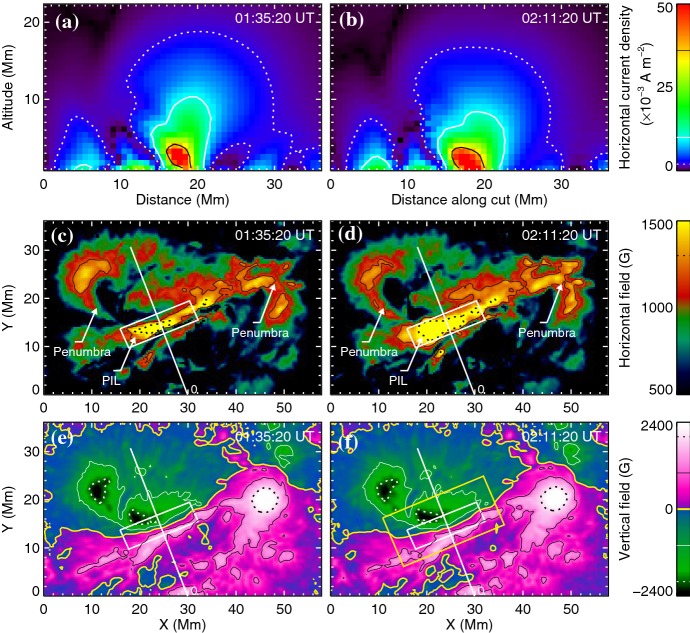
Modeled and observed field changes from before (01:00 UT; **a**, **c**, and **e**) to after (04:00 UT; **b**, **d**, and **f**) the 2011 February 15 X2.2 flare. **a**, **b** Current density distribution on a vertical cross section indicated in **c**–**f**. **c**, **d** HMI horizontal field strength. Contour levels are 1200 G and 1500 G. **e**–**f** HMI vertical field. Contour levels are $\pm \, 1000\, \mathrm{G}$ and $\pm \, 2000\, \mathrm{G}$. Image reproduced by permission from Sun et al. (2012), copyright by AAS

Associated with the above findings in the 2D photospheric magnetic fields, there must be a corresponding magnetic field evolution in 3D above the photosphere. The NLFFF extrapolation works as a powerful tool to reconstruct the 3D magnetic topology of the solar corona (see Sect. 4.3.1 for the extrapolation methods). Using Hinode/SOT magnetic field data, Jing et al. (2008) showed that the magnetic shear (indicating non-potentiality) only increases at lower altitude while it still largely relaxes in the higher corona, therefore the total free magnetic energy in 3D volume should still decrease after energy release of a flare. Using HMI data, Sun et al. (2012) clearly showed that the electric current density indeed increases at the flaring PIL near the surface while it decreases higher up, which may explain the overall decrease of free magnetic energy together with a local enhancement at low altitude (see Fig. 56). The above results may also imply that magnetic fields collapse toward the surface. Such a collapse was even detected in a C7.4 flare on 2012 July 2 as reported by Jing et al. (2014) and shown in Fig. 54. The collapse (or contraction) of magnetic arcades as reflected by NLFFF models across the C7.4 flare is spatially and temporally correlated with the formation of sunspot penumbra on the surface (Wang et al. 2013), as observed in high resolution observations of GST. The physics of this phenomenon is not fully understood: this could be due to newly reconnected magnetic fields above the PIL, or perhaps the reduction of local magnetic pressure due to a removal/weakening of the magnetic flux rope instigates the collapse.

**Fig. 57:**
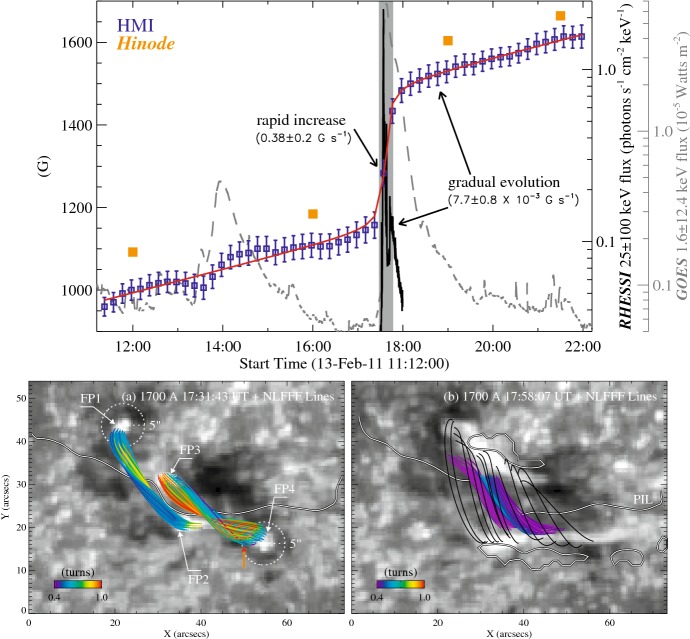
(Top) Temporal evolution of horizontal magnetic field measured by HMI and Hinode/SOT in a compact region around the PIL, in comparison with X-ray light curves for the M6.6 flare on 2011 February 13. The red curve is the fitting of HMI data with a step function. (Bottom) Extrapolated NLFFF lines before and after the event, demonstrating the process of magnetic reconnection consistent with the tether-cutting reconnection model. Images reproduced by permission from Liu et al. (2012, 2013), copyright by AAS

Using vector magnetograms from HMI together with those from Hinode/SOT with high polarization accuracy and spatial resolution, Liu et al. (2012) revealed similar rapid and persistent increase of the transverse field associated with the M6.6 flare on 2011 February 13, together with the collapse of coronal currents toward the surface at the sigmoid core region. Liu et al. (2013) further compared the NLFFF extrapolations before and after the event (see Fig. 57). The results provide direct evidence of the tether-cutting reconnection model. There are four flare footpoints. About 10% of the flux (${\sim }\, 3\times 10^{19}\, \mathrm{Mx}$) from the inner footpoints (e.g., FP2 and FP3 of loops FP2–FP1 and FP3–FP4) undergoes a footpoint exchange to create shorter loops of FP2–FP3. This result presents the rapid/irreversible changes of the transverse field and corresponding 3-D field changes in corona. A more comprehensive investigation including the 3D magnetic field restructuring and flare energy release as well as the helioseismic response of two homologous flares, the 2011 September 6 X2.1 and September 7 X1.8 flares in AR NOAA 11283, was performed by Liu et al. (2014). Their observational and modeling results depicted a coherent picture of coronal implosions, in which the central field collapses while the peripheral field turns vertical, consistent with what was found by Liu et al. (2005).

**Fig. 58:**
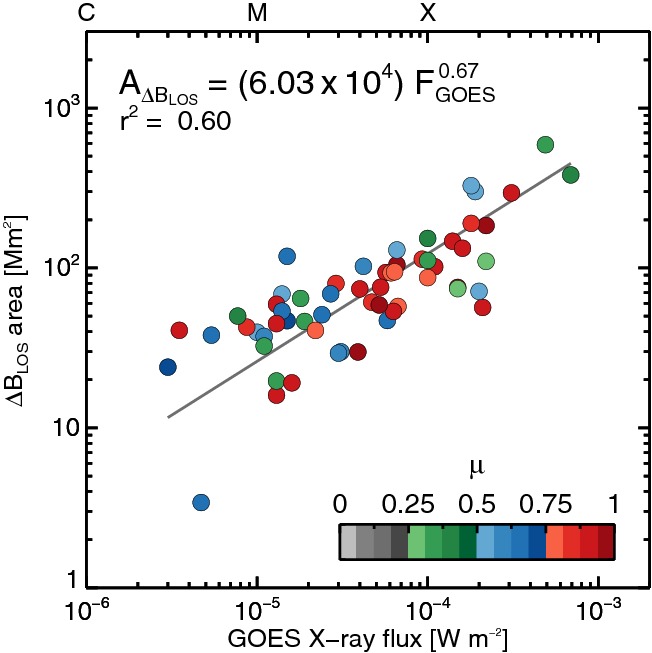
Area affected by rapid field changes corrected for foreshortening of LOS magnetic field as a function of the peak GOES soft X-ray flux of 75 events. Color-coded circles denote the center-to-limb distance $\mu$ (cosine of the heliocentric angle) of each event. The line is the best fit to a power law with a correlation coefficient of 0.6. Image reproduced by permission from Castellanos Durán et al. (2018), copyright by AAS

There are two research directions that are particularly worth mentioning here.

- Joint analysis of photospheric magnetic fields and coronal topology. Petrie (2016) studied two X-class flares observed by SDO and the Solar Terrestrial Relations Observatory (STEREO; Kaiser et al. 2008). They found that the rapid changes of magnetic fields at the PIL is associated with coronal loop contraction. Gömöry et al. (2017) analyzed VTT (Vacuum Tower Telescope) data covering an M-class flare and found an enhancement of the transverse magnetic field of approximately 550 G. This transverse field was found to bridge the PIL and connect umbrae of opposite polarities in the $\delta$-spot. At the same time, a newly formed system of loops appeared co-spatially in the corona as seen in 171 Å passband images of SDO/AIA. Therefore, the rapid photospheric magnetic field evolution is a part of 3D magnetic field re-structuring.
- Statistical study of a large number of events. Castellanos Durán et al. (2018) carried out a statistical analysis of permanent LOS magnetic field changes during 18 X-, 37 M-, 19 C-, and 1 B-class flares using data from SDO/HMI. They investigated the properties of permanent changes, such as frequency, areas, and locations. They detected changes of LOS field in 59 out of 75 flares and found that the strong flares are more likely to show changes. Figure 58 demonstrates the correlation between the affected LOS field change area and the peak GOES flux. It is apparent that larger flare produces more prominent field changes.

### Sudden sunspot rotation and flow field changes

The evolution of magnetic fields is closely associated with photospheric flow motions. Obviously, the studies of the flow fields along with the magnetic field evolution is very important. Several methods of flow tracking have been developed as summarized and compared by Welsch et al. (2007). One particular method is the differential affine velocity estimator (DAVE; Schuck 2005, 2006) that uses the induction equation to derive flow fields. A substantially improved version, DAVE for vector magnetograms (DAVE4VM; Schuck 2008), derives not only the horizontal but also the vertical component of the flows, which thus can analyze the flux emergence (i.e., vertical motions) in addition to the horizontal motions.

Wang et al. (2014) showed some initial results of the flare-related acceleration of sunspot rotation that is derived by DAVE using SDO/HMI observations of AR NOAA 11158. The rotational speeds of the two sunspots increase significantly during and right after the X2.2 flare. Moreover, the direction of the enhanced sunspot rotation agrees with that of the change of the horizontal Lorentz force. Using the estimated torque and moment of inertia, Wang et al. (2014) estimated the angular acceleration of the sunspots. Although there are some uncertainties in the measurements and assumptions, the values agree with the observed angular acceleration of suddenly rotating sunspot immediately after the flare.

**Fig. 59:**
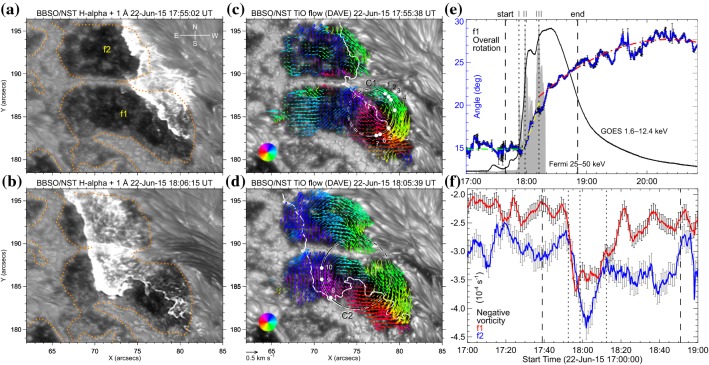
BBSO/GST chromospheric H$\alpha +1$ Å images showing flare ribbons (**a**, **b**) and the corresponding photospheric TiO images (**c**, **d**). In panel **a**, sunspots are labeled as f1 and f2, with the dotted lines contouring the vertical magnetic field at 1300 G. In panels **c** and **d**, the superimposed arrows (color-coded by direction; see the color wheel) depict the differential sunspot rotation tracked with DAVE. The thick white curves are the co-temporal flare ribbon. **e** Temporal evolution of overall sunspot rotation, showing the orientation angle of f1 from an ellipse fit (blue) and its approximation using an acceleration plus a deceleration function. **f** Temporal evolution of vorticity derived based on DAVE velocity vectors indicating the accelerated sunspot rotation. Image reproduced by permission from Liu et al. (2016a), copyright by the authors. (For movie see Electronic Supplementary Material)

Liu et al. (2016a) used GST data to analyze the flow motions of the 2015 June 22 M6.6 flare. It is particularly striking that the rotation is not uniform over the sunspot: as the flare ribbon sweeps across, its different portions accelerate (up to $50^{\circ }\,\mathrm{h}^{-1}$) at different times corresponding to peaks of the flare hard X-ray emission. Associated with the rotation, the intensity and magnetic field of the sunspot change significantly, and the Poynting and helicity fluxes temporarily reverse their signs, indicating that the energy propagation that causes the rotation is from the higher atmosphere down to the photosphere. Figure 59 demonstrates the key results of that study (see also the corresponding movie).

**Fig. 60:**
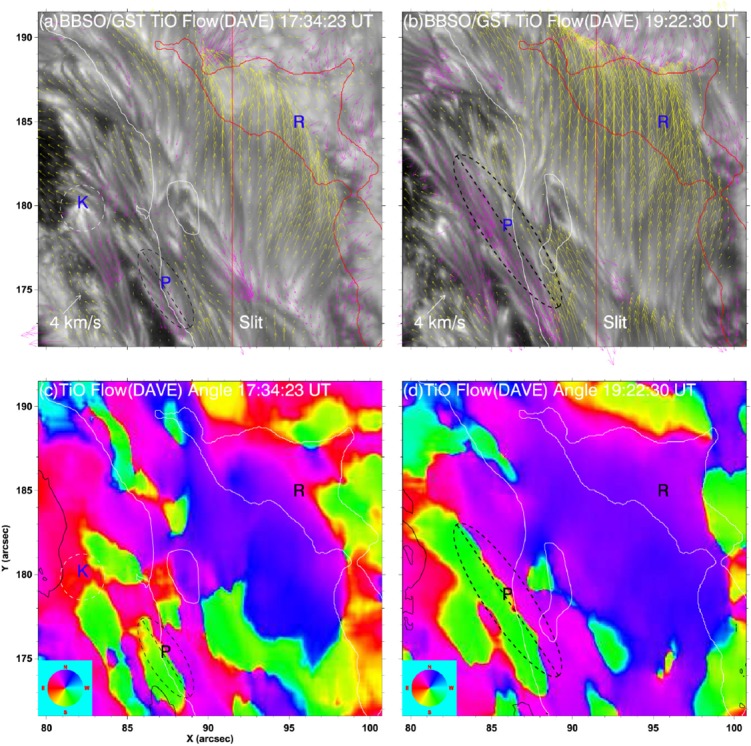
Flow field in the BBSO/GST TiO band. **a**, **b** Pre-flare (at 17:34:23 UT) and post-flare (at 19:22:30 UT) TiO images overplotted with arrows illustrating the flow vectors derived with DAVE. For clarity, arrows pointing northward (southward) are coded yellow (magenta). **c**, **d** Azimuth maps of corresponding flow vectors in panels **a**, **b**, also overplotted with the PIL, precursor kernel, and region R contours. The shear flow region P showing the most obvious flare-related enhancement is outlined using the dashed ellipse, with its major axis quasi-parallel to the PIL. Image reproduced by permission from Wang et al. (2018b), copyright by AAS

Wang et al. (2018b) analyzed the same AR with GST and HMI data. For a penumbral segment in the negative field adjacent to the PIL, an enhancement of penumbral flows (up to an unusually high value of $2\, \mathrm{km\ s}^{-1}$) and extension of penumbral fibrils after the first peak of the flare hard X-ray emission. They also found an area at the PIL, which is co-spatial with a precursor brightening kernel, that exhibits a gradual increase of shear flow velocity (up to $0.9\, \mathrm{km\ s}^{-1}$) after the flare. The enhancing penumbral and shear flow regions are also accompanied by an increase of horizontal field and decrease of magnetic inclination angle measured from the horizontal. These results further confirm the concept of back reaction of coronal restructuring on the photosphere as a result of flare energy release. Figure 60 shows the evolution of the flow fields covering the flare.

### Theoretical interpretations

The modeling efforts of ARs and related eruptions are summarized in Sect. 4. Here we review certain points in explaining magnetic field restructuring following flares. Longcope and Forbes (2014) reviewed solar eruption models and classified them into three categories, tether-cutting, break-out and loss-of-equilibrium, all of which can be catastrophic. The tether-cutting model assumes a two-step reconnection that leads to eruption in the form of flares and CMEs, in particular, for sigmoid ARs (e.g., Moore and Labonte 1980; Moore et al. 2001; Moore and Sterling 2006). The first-stage reconnection occurs near the solar surface at the onset of the eruption and produces a low-lying shorter loop across the PIL and thus explains the observed enhancement of transverse fields after flare. It also produces a much longer twisted flux rope connecting the two far ends of a sigmoid that triggers the second stage of eruption: the twisted flux rope becomes unstable and erupts outward to form a full CME.

It is possible that in the earlier phase of the eruption, contraction of the shorter flare loop occurs. This has received increasing attention recently (e.g., Ji et al. 2006) and possibly corresponds to the first stage of the tether cutting. The ribbon separation described in the standard flare models such as the CSHKP model (Sect. 2.2) manifests the second stage. This model may explain other observational findings such as (1) transverse magnetic field at flaring PILs increases rapidly/persistently immediately following the flares (Wang et al. 2002b, 2004b; Wang and Liu 2010); (2) penumbral decay occurs in the peripheral penumbral areas of $\delta$-spots, indicating that the magnetic field lines turn more vertical after a flare in these areas (Wang et al. 2004a; Liu et al. 2005); and (3) hard X-ray images of the Reuven Ramaty High Energy Solar Spectroscopic Imager (RHESSI; Lin et al. 2002) show four footpoints, two inner ones and two outer ones, and sometimes the hard X-ray emitting sources change from confined footpoint structure to an elongated ribbon-like structure after the flare reaches intensity maximum (Liu et al. 2007a, b).

In an attempt to quantitatively compare observations and modeling, Li et al. (2011) compared idealized MHD simulation of emerging flux in flare triggering with observation. They selected a lower level in the simulation to examine the near-surface magnetic structure evolution. Changes of magnetic field orientation and strength in the photosphere after flares/CMEs are indeed found in the simulation. The most obvious match is at the flaring PIL, where field lines in the simulation are found to be more inclined towards the horizontal, and transverse field strength increased after the eruption. At the outer side of the simulated sunspot penumbral area, field lines turn to a more vertical direction with a decreased transverse field strength. These are consistent with the observed penumbral enhancement at the PIL and decay of peripheral penumbrae (Liu et al. 2005). The simulation also shows the downward net Lorentz force pressing onto the photosphere, confirming the related observations.

**Fig. 61:**
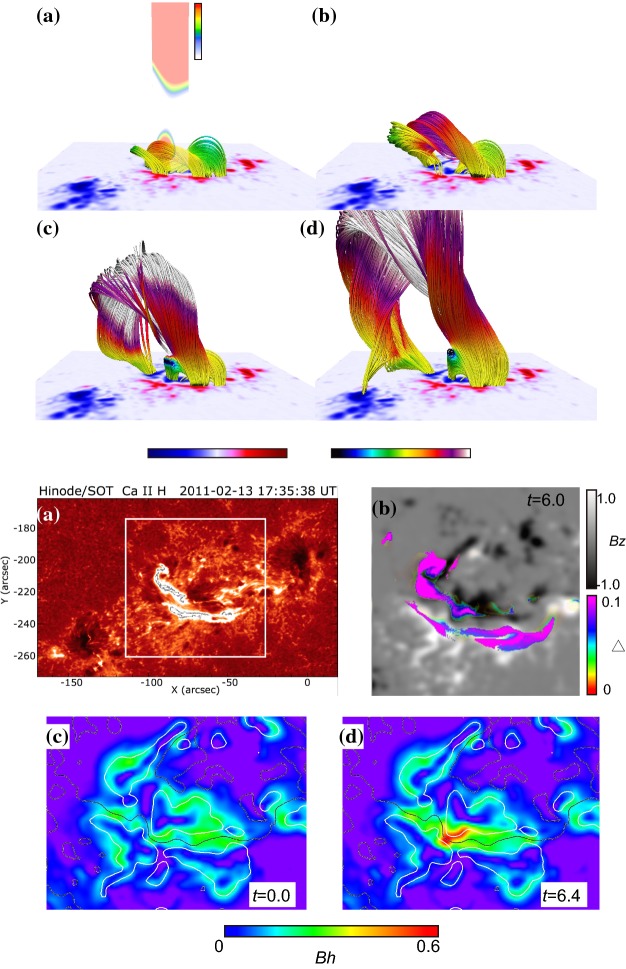
(Top) Temporal evolution of the modeled 3D dynamics of the eruptive flux rope on 2011 February 13 in AR NOAA 11158, together with the $B_{z}$ distribution at the bottom. (Bottom) Comparison of simulation results with observations. **a** Flare ribbons during the M6.6 flare, observed by Hinode at 17:35 UT. **b** Synthetic flare ribbons measured from total displacement of the field line superimposed on the $B_{z}$ distribution. The area corresponds to one surrounded by white square in panel **a**. **c**, **d** $B_{h}$ distributions obtained from the simulation, just prior to and during the eruption, respectively. $B_{h}$ increased prominently across the main PIL (marked by the black lines). Image reproduced by permission from Inoue et al. (2018a), copyright by the authors

Recently, Inoue et al. (2018a) performed an MHD simulation that takes into account the observed photospheric magnetic field to reveal the dynamics of a solar eruption in a realistic magnetic environment. In this simulation, they confirmed that the tether-cutting reconnection occurring locally above the PIL creates a twisted flux tube, which is lifted into a toroidal unstable area where it loses equilibrium, destroys the force-free state, and drives the eruption. Figure 61 shows that the simulation not only reproduces the flare ribbons well but also demonstrates the irreversible transverse field enhancement at the photospheric PIL. Although the authors did not emphasize this point, the peripheral penumbral decay is also apparent in the simulated data. The same event has been analyzed in detail observationally by Liu et al. (2012, 2013). Note that Inoue et al. (2015) demonstrated similar field changes for the X2.2 flare in the same AR. The rapid field change coincides with the onset of the flare.

As we mentioned earlier, Hudson et al. (2008) and Fisher et al. (2012) introduced the back reaction concept. The authors made the prediction that after flares, at the flaring PIL, the photospheric magnetic fields become more horizontal. The analysis is based on the simple principle of energy and momentum conservation: the upward erupting momentum must be compensated by the downward momentum as the back reaction. In addition, the field change should be stepwise (i.e. permanent) because it results from the removal of magnetic energy and magnetic pressure from the corona. This is one of the few models that specifically predict the rapid and permanent changes of photospheric magnetic fields associated with flares and support the observed Lorentz force change (e.g., Wang et al. 2012b, c; Liu et al. 2012; Sun et al. 2012; Petrie 2013, 2014, 2019).

As a more recent study, Wang et al. (2018c) analyzed four flare events using SDO/AIA and STEREO and demonstrated the existence of real contractions of loops. They identified two categories of implosion, which are (1) a rapid contraction at the beginning of the flare impulsive phase, as magnetic free energy is removed rapidly by a filament eruption; and (2) a continuous loop shrinkage during the entire flare impulsive phase that corresponds to ongoing conversion of magnetic free energy in a coronal volume.

**Fig. 62:**
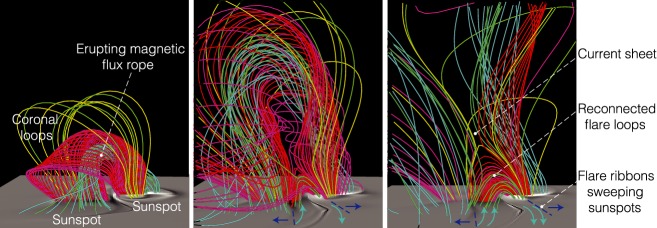
Series of snapshots, from left to right, of a realistic numerical simulation of an eruptive flare. The colored lines show representative coronal magnetic field lines plotted from fixed footpoints in the photosphere: the cyan field lines represent the erupting flux rope, and the red (green) field lines are those that eventually reconnect with pink (yellow) field lines. The gray scale plane shows the time-varying electric current densities in the photosphere. The blue arrows show the displacement of the ribbons and cyan curved arrows indicate how sunspot rotation is initiated as flare ribbons move across sunspots. Image reproduced by permission from Aulanier (2016), copyright by Macmillan

Finally, in Aulanier (2016), the sudden sunspot rotation is somehow demonstrated in their simulation (see Fig. 62). Note that these simulations usually assume the line-tying condition, i.e., the footpoint motions are not allowed (see Sect. 4.2 for details). Nevertheless, the observed trend slightly above the photosphere can demonstrate the direction for the rotational force, although quantitative comparison is very difficult.

## Summary

How close have we reached to the complete picture of the formation and evolution of flare-producing ARs? Thanks to the advancement of observation techniques and modeling efforts, we have acquired a substantial amount of knowledge that may set the grounds for a more complete understanding. In this section, we summarize our current understanding of the genesis and evolution and key observational features of these ARs.

### The era with Hinode, SDO, and GST

To a greater degree, our understanding of the flaring ARs has been pushed forward by the ceaseless improvement of observation instruments, and the progress in the last decade has been made in particular by Hinode, SDO, and GST. In fact, many parts of this review article are based on the outcome of these missions.

Since launch in September 2006, the Hinode spacecraft has sent us various important observables. By virtue of seeing-free condition from space, one of its trio of instruments, SOT, has acquired high-resolution vector magnetograms, revealed the detailed structure of flaring PILs, and showed us its importance in triggering flares and CMEs (Sect. 3.2.1). With the vector magnetograms, though not quite satisfactorily, now we can extrapolate the coronal field by the NLFFF techniques, which is used as the initial condition of data-constrained simulations (Sect. 4.3). Moreover, through simultaneous multi-wavelength observation in concert with XRT and EIS, Hinode realized even more comprehensive tracing of the dynamical evolution over the different atmospheric layers. The flux rope formation due to the photospheric shear motion and the non-thermal broadening of EUV lines in response to the helicity injection are good examples of Hinode’s multi-wavelength probing of flare-producing ARs (Sects. 3.3.1 and 3.3.2).

Everyday, tons of observational data are ceaselessly poured to the ground from SDO (launched in February 2010). They include photospheric intensitygram, Dopplergram, (vector) magnetogram, and (E)UV images. Its constant full-disk observation enables us to statistically investigate the evolution of ARs from appearance to eventual flare eruption with unprecedented details. Together with EIS and XRT, the multi-filter (multi-temperature) observation of AIA provided the thermal diagnostics of ARs such as DEM inversions (Sect. 3.3.1). The steady supply of vector magnetogram by HMI revealed the rapid changes of not only the LOS field but also the transverse field in time scales of down to ${\sim }\, 10$ min (Sect. 5.2). Several new attempts to utilize vector data have started. For instance, the series of vector magnetograms are used in data-driven simulations to sequentially update the boundary condition of coronal field models (Sect. 4.3.3). Various photospheric parameters calculated from the vector data are used for predicting the flares and CMEs (see discussion in Sect. 7.2.1).

Thanks to the high spatial resolution with the 1.6-m aperture and the longer duty cycle, BBSO/GST (scientific observation initiated in January 2009) has played a key role in obtaining insights into the rapid changes of photospheric (high-$\beta$) fields in response to dynamical evolution of coronal (low-$\beta$) fields during the course of flares and CMEs (Sect. 5). The most important science outputs made by BBSO/GST related to the flare-AR science include (1) the detailed structure, development, and destabilization of a flux rope, (2) the sudden flare-induced rotation of sunspots and evolution of photospheric flow fields, and (3) the tiny and transient flare precursors in the lower atmosphere. Through these discoveries, now we know that the answer to the “tail wags the dog” problem, i.e., whether the coronal eruption can cause changes in the photospheric field, is yes.

The advancement of instruments has also motivated the development of numerical modeling. For instance, the long-term monitoring of flare-productive ARs by Hinode and SDO from birth to eruption inspired the flux emergence models and gave a clue to the formation mechanisms of $\delta$-spots (e.g., NOAA 11158 in February 2011: Sect. 4.1.2). Fine-scale flare-triggering fields and rapid magnetic changes during the flares, which are observable only with advanced instruments, have been compared with the results of the flare simulations (Sects. 4.2 and 5.4). Filtergram images of various wavelengths by XRT and AIA provide the means to diagnose the coronal fields (e.g., XRT image and NLFFF extrapolation of sigmoids: Sect. 3.3.1). All of these results underscore the importance of direct comparison of observation and modeling in unraveling the formation and evolution of flare-producing ARs.

### From birth to eruption

In this subsection, we summarize some of the key aspects related to the genesis of flare-producing ARs and eventual energy release, which have been uncovered by the observational and theoretical studies presented in this review article.

1. Subsurface evolution: The dynamo-generated toroidal flux loops start rising in the convection zone (Sect. 2.1). Subject to the background turbulent convection, some of them may lose a simple $\varOmega$-shape and deform into a helical structure, a top-dent configuration, bifurcated multiple branches, or collide with other flux systems (Sect. 4.1). Through these processes, the rising flux systems gain non-potentiality that is represented by free magnetic energy and magnetic helicity.
2. Formation of $\delta$-spots: On their appearance in the photosphere, some of these rising flux loops form $\delta$-sunspots, in which umbrae of positive and negative polarities are so close to share a common penumbra (Sect. 2.3). Most of the $\delta$-spots are generated by multiple emerging loops rather than a single $\varOmega$-loop and the diversity of polarity layout stems from the difference in the subsurface history, but strong flares also emanate from non-$\delta$ sunspots such as the Inter-AR case (Sect. 3.1).
3. Development of flaring PIL and photospheric features: Due to shearing and converging motion, the PIL between the opposite polarities obtains a strong transverse field with high gradient and shear (Sect. 3.2). This is the outcome of the Lorentz force, and this force also causes the rotational motion of sunspots (Sect. 4.1).
4. Formation of flux rope: The coronal fields lying above the PIL become sheared in sync with the photospheric driving, cancel against each other, and form a magnetic flux rope. This helical structure is observed as a sigmoid in soft X-rays and as a filament (prominence) in H$\alpha$ (Sects. 3.3 and 4.2).
5. Flare occurrence and CME eruption: When the energy is sufficiently accumulated, the solar flare is eventually initiated (Sect. 2.2). The flux rope becomes destabilized and erupts, often as a CME into the interplanetary space, leaving behind a variety of remarkable observational features on the Sun. The drastic evolution of coronal fields causes rapid and profound changes in magnetic and flow fields even in the photosphere (Sect. 5). If the confinement of the overlying arcade in an AR is too strong, however, the flux rope may not develop into a CME.

As is obvious from the fact that helical structures are seen in many parts in the story above, the whole process of AR formation, flare eruption, and CME propagation appears to be, overall, the large-scale transport of magnetic helicity and energy from the solar interior all the way to outer space (Low 1996, 2002; Démoulin 2007). In this sense, the formation of $\delta$-spots, where abundant evidence of non-potentiality is observed, is accepted as a natural consequence of the helicity that is delivered from the interior.

### Key observational features and quantities

In the long history of observation of ARs producing strong flares and CMEs, various features have been investigated. Perhaps these features can be summarized into three important factors, which are (a) the size, (b) complexity, and (c) evolution. Given the large magnetic energy accumulated in the ARs, it is reasonable that these ARs are larger in spot area, or naturally in total magnetic flux. However, as we saw in Sect. 2.2, the largest spot in history, RGO 14886, was not flare active, probably because this AR had a simple bipolar (i.e., potential) magnetic field. To increase free magnetic energy that is released through flare eruptions, ARs need to contain morphological and magnetic complexity, which is manifested as the dispersed polarities (i.e., $\gamma$-spots), strong-field, strong-gradient, highly-sheared PILs in $\delta$-spots, magnetic tongues, flux ropes, sigmoids, etc. These complex structures manifest during the course of AR evolution, observed as flux emergence of various scales, shearing motion on both sides of a PIL, and rotational motion of the sunspots. Of course, such evolutionary processes may serve as a trigger of eventual flare eruption.

**Table 1:** Some selected parameters in the literature that address the productivity of X-class flares

Parameter	Production of X-class flares	Reference
Spot area	40% of $\ge$ 1000 MSH $\beta \gamma \delta$-spots	Sammis et al. (2000)
PIL total unsigned flux (*R*-value)	20% of $\log {(R)}=5.0$ (within the next 24 h)	Schrijver (2007)
Fractal dimension	$\ge 1.25$	McAteer et al. (2005)
Power-law index	$>2.0$	Abramenko (2005)
Peak helicity injection rate	$\ge 6\times 10^{36}\, \mathrm{Mx}^{2}\, \mathrm{s}^{-1}$	LaBonte et al. (2007)
Total non-neutralized current	$\ge 4.6\times 10^{12}\, \mathrm{A}$	Kontogiannis et al. (2017)
Maximum non-neutralized current	$\ge 8\times 10^{11}\, \mathrm{A}$	Kontogiannis et al. (2017)
Normalized helicity gradient variance	1.13 (1 day before the flare)	Reinard et al. (2010)

As we have seen in many parts in this review, there is a multitude of statistical investigations that reveal the quantitative differences between flaring and quiescent ARs. In Table 1, we pick up several parameters from the literature that are suggested to differentiate (and may subsequently predict) X-class flares.

One may notice from this table and other references in this article that many of the variables that have been investigated so far are snapshot parameters, i.e., those derived from observation at a single moment. However, since it is the AR evolution that drives the flaring activities, we need to understand the importance of dynamic parameters, i.e., those that describe the temporal change of magnetic fields. One of the most striking examples is the very fast flux emergence in the super-flaring AR NOAA 12673 (Fig. 31). Sun and Norton (2017) showed that the flux growth rate (i.e., time derivative of unsigned total magnetic flux) in this AR was greater than any values reported in the literature, and its X9.3 flare occurred a couple of days after this remarkable emergence was detected. Therefore, such time derivative quantities might be key to predict flares and CMEs (Sect. 7.2.1; see also Leka and Barnes 2003, 2007).

## Discussion

Despite the remarkable progress made to date, many outstanding questions remain. However, some of them will be answered if observational and numerical techniques are improved more in the near future. In this section, we list some of the important questions and discuss the possibilities to utilize our knowledge of flare-productive ARs in related science fields.

### Outstanding questions and future perspective

Observationally, we still do not have a “visual” image of the subsurface emerging flux and thus we cannot establish whether the complex 3D configuration of flaring ARs deduced from the surface evolution is real or not. In a statistical sense, on average, these ARs show enhanced vorticity before they cause flare eruptions (Sect. 3.3.3). However, we still do not have robust methods of imaging the rising flux because the (local) helioseismic probing is hampered by the fast emergence and the low signal-to-noise ratio. The existence of strong flux may not be treated as a small perturbation, which is assumed when solving the linear inverse problem in seismology. Advancement in helioseismology techniques, probably with the support of numerical modeling, is desired to overcome this difficulty.

Turbulent convection plays a crucial role in producing the morphological and magnetic complexity of these ARs. The generation of $\varOmega$-loops from the magnetic wreath in the global anelastic simulations begins to establish the concept of the “spot-dynamo” (Fig. 2: see Nelson et al. 2013; Brun et al. 2015). However, due to the limitation of the anelastic approximation, it is difficult to trace the story after the flux loops pass through the uppermost convection zone (about $-\,20\, \mathrm{Mm}$ and upward). Compressible simulations that enable access to (very close to) the solar surface, such as by Hotta et al. (2014), may reveal the dynamical interaction between the magnetic field and turbulent convection in much greater detail. The genesis of magnetic helicity, namely, the twist and writhe of emerging flux (observed in the form of magnetic shear, spot rotations, magnetic tongues, sigmoids, etc.: Sect. 3), is still a big mystery (Longcope et al. 1999). Regarding the formation of flaring ARs, it is also an interesting question how and why super strong transverse field appears at the PIL in a $\delta$-spot instead of at the core of sunspot umbra. These issues may be solved by an advancement of numerical models.

There has been a dichotomy of theory whether a magnetic flux rope is created well before the eruption or at the very moment of it (see, e.g., Forbes et al. 2006, p. 266). Thanks to the NLFFF, data-constrained, and data-driven models, now the flux rope appears to be created from before eruption, at least in the flare-productive ARs, through the continued shearing along the PIL. These numerical methods may be advanced even more and provide a conclusive answer. For example, vector field measurements in higher atmospheric layers may realize more accurate extrapolations. In the current force-free methods, it is assumed that the input photospheric vector field is in force-free (Sect. 4.3.1). However, this is apparently not the case because the photosphere is in the realm of high-$\beta$ plasma (i.e., the photospheric plasma is largely affected by the non-magnetic forces such as pressure gradient), which requires a smoothing of the photospheric vector field before the extrapolation is applied. Chromospheric low-$\beta$ fields, obtained by future instruments such as the Daniel K. Inouye Solar Telescope (DKIST), may give better boundary conditions for the force-free extrapolations, data-constrained and data-driven models. Moreover, magnetic information at multiple altitudes allows us to calculate the partial derivatives in the vertical direction (i.e., $\partial B_{x}/\partial z$ and $\partial B_{y}/\partial z$) and may provide better estimates of the total (vector) current density, horizontal velocity, electric field, and Lorentz force density.

Stereoscopic monitoring of the Sun from multiple vantage points, for instance by spacecrafts around the Earth and at the Lagrangian L5 point or by off-ecliptic explorers like Solar Orbiter, is helpful in various aspects (Akioka et al. 2005; Schrijver et al. 2015; Gibson et al. 2018). Apart from the early warning of space weather events like Earth-directed CMEs and violent ARs beyond the east limb, it may help probing the deeper interior with local helioseismology, resolving the ambiguity of magnetic measurements, and assessing the topology of entangled coronal fields (see results from STEREO). With advanced spectroscopic and imaging instruments, atmospheric evolution such as build-up and eruption of flux ropes and non-thermal broadening of EUV lines (Sect. 3.3) may be revealed in further detail. All these new capabilities will greatly improve our understanding of the nature of flare-productive ARs.

The detection of flare-related activities from ground-based large-aperture telescopes has been, in most cases, done by GST (Sect. 5). To better understand the fine-scale dynamics in AR build-up and flare eruption, it is necessary to increase the detection rate of these events by enhancing the observing time. One possible idea is to organize an international network of high-resolution telescopes, such as DKIST (4-m aperture in Maui), New Vacuum Solar Telescope (NVST; 1-m aperture in Yunnan), Swedish Solar Telescope (SST; 1-m aperture in La Palma), GREGOR (1.5-m aperture in Tenerife), and European Solar Telescope (EST; 4-m aperture under contemplation), and conduct a long-running monitoring of a target AR. Several key observations of dynamic activities in flaring ARs were already made with NVST (Xue et al. 2016, 2017) and SST (Guglielmino et al. 2016; Robustini et al. 2018). Therefore, the combination of these stations may open up unexplored discovery space and provide insights into the evolution of small-scale magnetic features in the very long run (days to weeks).

### Broader impacts on related science fields

#### Prediction and forecasting of solar flares and CMEs

Probably one of the most practical applications of the knowledge of flaring ARs we have acquired is the prediction of flares and CMEs. Statistical investigations of various events that introduce parameters such as those in Table 1 characterized the flare-productive ARs. In the last decades, the knowledge-based flare predictions using these quantities have been significantly developed.

**Table 2:** 13 flare-predictive parameters derived from the SDO/HMI vector data (Bobra and Couvidat 2015)

Description	Formula	*F*-score
Total unsigned current helicity	$H_{c_{\mathrm{total}}}\propto \sum |B_{z}\cdot J_{z}|$	3560
Total magnitude of Lorentz force	$F\propto \sum B^{2}$	3051
Total photospheric magnetic free energy density	$\rho _{\mathrm{tot}}\propto \sum ({{\mathbf {B}}}^\mathrm{Obs}-{{\mathbf {B}}}^\mathrm{Pot})^{2}dA$	2996
Total unsigned vertical current	$J_{z_{\mathrm{total}}}=\sum |J_{z}|dA$	2733
Absolute value of the net current helicity	$H_{c_{\mathrm{abs}}}\propto \left| \sum B_{z}\cdot J_{z}\right|$	2618
Sum of the modulus of the net current per polarity	$J_{z_{\mathrm{sum}}}\propto \left| \sum ^{B_{z}^{+}} J_{z}dA\right| +\left| \sum ^{B_{z}^{-}} J_{z}dA\right|$	2448
Total unsigned flux	$\varPhi =\sum |B_{z}|dA$	2437
Area of strong field pixels in the active region	$\mathrm{Area}=\sum \mathrm{Pixels}$	2047
Sum of *z*-component of Lorentz force	$F_{z}\propto \sum (B_{x}^{2}+B_{y}^{2}-B_{z}^{2})dA$	1371
Mean photospheric magnetic free energy	$\overline{\rho }\propto \frac{1}{N}\sum ({{\mathbf {B}}}^\mathrm{Obs}-{{\mathbf {B}}}^\mathrm{Pot})^{2}$	1064
Sum of flux near polarity inversion line	$\varPhi =\sum |B_{LoS}|dA$ within *R* mask	1057
Sum of *z*-component of normalized Lorentz force	$\delta F_{z}\propto \frac{\sum (B_{x}^{2}+B_{y}^{2}-B_{z}^{2})}{\sum B^{2}}$	864.1
Fraction of area with shear $> 45^{\circ }$	Area with shear $> 45^{\circ }$/total area	740.8

*F*-score indicates the scoring of the parameter

Nowadays, these methods employ machine-learning algorithms. For example, Bobra and Couvidat (2015) extracted various photospheric parameters from the SDO/HMI vector magnetograms for individual ARs, trained the machine, and obtained a good predictive performance for $\ge$ M1.0 flares. The parameters investigated are listed in Table 2, which are basically the previously suggested variables (Leka and Barnes 2003; Fisher et al. 2012; Schrijver 2007), It should be noted that most of them are “extensive,” where a given parameter increases with AR size (Tan et al. 2007; Welsch et al. 2009; Sun et al. 2015; Toriumi and Takasao 2017).

Many of the parameters listed in Table 2 are, again, snapshot ones (see Sect. 6.3), and the inclusion of dynamic parameters may be helpful in flare predictions (Leka and Barnes 2003, 2007). For instance, to the flare-predictive parameters in Table 2, Nishizuka et al. (2017) added additional information that indicates flare history and chromospheric pre-flare brightening and also time derivatives of various observables. By training the machine with three different algorithms, the authors successfully obtained a prediction score higher than that of Bobra and Couvidat (2015). This study clearly highlights the usage of dynamic parameters.

However, it is worth noting that increasing the number of parameters does not necessarily improve the prediction performance. In fact, Leka and Barnes (2007) and Bobra and Couvidat (2015) found that there was little value to add parameters more than a few. This is because the model with many parameters (i.e. large degrees of freedom) tends to overfit the training data and, in that case, the model may perform worse on the validation data.

Today, while there remains a view that the occurrence of flares is a “stochastic” process (e.g., the avalanche model by Lu and Hamilton 1991) and therefore the “deterministic” forecasting might be fundamentally impossible (Schrijver 2009), the knowledge-based prediction is growing much more rapidly than ever before (e.g., Qahwaji and Colak 2007; Colak and Qahwaji 2009; Yu et al. 2010; Ahmed et al. 2013; Muranushi et al. 2015; Bobra and Ilonidis 2016; Liu et al. 2017a; Jonas et al. 2018; Huang et al. 2018; Nishizuka et al. 2018). Together with the attempts to build up physics-based (i.e., modeling-based) algorithms (Sects. 4.3.2 and 4.3.3), the recent development of this field may tell us that the real-time space weather forecasting will come true in the very near future.

#### Investigating extreme space-weather events in history

The strongest flare activity ever observed with an estimated GOES class of ${\sim }$X45 is the Carrington flare in September 1859 (see Sect. 2.2). To understand the mechanisms and trends of such extreme space weather events that may affect the Earth (like the occurrence frequency; Schrijver et al. 2012; Riley 2012; Curto et al. 2016), it is crucial to increase the sample number by surveying the greatest events in history. However, often these events do not have observations of sufficient data quality for scientific analysis. In the modern age, the data analyzed are often digitized intensity images of various wavelengths and LOS or vector magnetograms. For the historical events, however, available records can be photographic plates or perhaps only sunspot drawings. But still, there are several ways to elucidate how and why the strong events occurred.

For instance, there are several attempts to achieve magnetic information from historical sunspot drawings. For the great storm of May 1921 (Silverman and Cliver 2001; Kappenman 2006), Lundstedt et al. (2015) reconstructed “magnetograms” by applying their torus model to the daily Mount Wilson drawings of sunspot magnetic fields and studied the development of the target AR. They found that spot rotations and flux emergence occurred in the AR. They pointed out the close association between the drastic spot evolutions and the eventual magnetic storm.

Another approach is to reconstruct vector magnetogram from existing LOS magnetogram by applying one of the machine-learning methods called transfer learning (Pan and Qiang 2010). One of the purposes of this method is to convert some source data to target data and, with this method, one may use SDO/HMI vector magnetograms (for Cycle 24) and SOHO/MDI LOS magnetograms (for Cycle 23) as the source data and target data, respectively, and reproduce “vector magnetograms” for ARs of Cycle 23. Because there were many more stronger flares in Cycle 23, such vector data may help investigate the driving mechanisms of extreme events.

In many respects, studying historical records is beneficial in understanding the activity of the Sun. It may tell us how strong events the Sun can produce, how frequently these events occur, and how they make an impact on our magnetic circumstances. Although it is not easy to derive useful information from such records, we can still take advantage of the current knowledge of flaring ARs. Attempts to examine drastic spot evolution and reconstruct magnetograms may give us clues to understand the nature of severe space-weather events.

#### Connection with stellar flares and CMEs

The production of stellar flares and CMEs are now of great importance, not only from the viewpoint of mass and angular momentum loss rates especially of the active young stars (e.g., Aarnio et al. 2012), but also in the search for habitability of orbiting exoplanets. The type II radio burst, which is believed to be produced by MHD shocks in front of the CME propagating into the interplanetary space (Gurnett 1995), is currently the best way of detecting the stellar CMEs (Osten and Wolk 2017).

In this regard, Crosley and Osten (2018a, b) attempted to detect type II bursts on nearby, magnetically-active, well-characterized M dwarf star EQ Peg. During 20 h of simultaneous radio and optical observation, they detected four optical flare signatures but no radio features identifiable as type II bursts. Two radio bursts were found during the additional 44 h of radio-only observation. However, their characteristics were not consistent with that of type II events. From the statistics of the solar flares and CMEs (Yashiro et al. 2006), all the four detected flares are empirically predicted to have associated CMEs, but none was detected at radio wavelengths in this data set.

As an independent analysis, Leitzinger et al. (2014) searched for flares and CMEs on 28 young late-type (K to M) stars in the open cluster Blanco-1. From the 5 h observation, they found four H$\alpha$ flares from three M stars and one K star. Interestingly, however, they also did not detect any clear indications of CMEs such as spectral asymmetries of the H$\alpha$ line caused by large Doppler velocities.

Although we cannot rule out the possibility that the signals were less than the detection sensitivity, it is worth discussing the reason of the “failed” eruptions by employing the knowledge of flare-productive ARs of the Sun. As we saw, for instance, in Sects. 2.2 and 4.1.5, the flare eruption tends to fail when the overlying coronal loops are strong and slowly decaying over height (Wang et al. 2017a; Vasantharaju et al. 2018; Jing et al. 2018). Observations and numerical modeling of flaring ARs show that, for the failed events, a magnetic flux rope is often trapped in the AR core and does not have an access to open fields (Toriumi et al. 2017b; Toriumi and Takasao 2017; DeRosa and Barnes 2018). As the Zeeman Doppler Imaging by Morin et al. (2008) suggests, active M dwarfs tend to be covered by strong magnetic patches over the entire stellar surface. Due to the strong confinement by coronal loops extending from these patches, we may expect less successful CME eruptions even if energetic stellar flares occur (Drake et al. 2016). The confinement may also be due to the strong large-scale dipolar field, as numerically modeled by Alvarado-Gómez et al. (2018).

Thanks to the advancement of observational capabilities, many more “superflares” are now detected on solar-like G-type stars (Maehara et al. 2012; Shibayama et al. 2013). Indications of huge starspots with large magnetic energy are seen in these stars (e.g., Notsu et al. 2013). By conducting spectroscopic and polarimetric observations on the properties of superflares and starspots, and by comparing them with numerical models of solar–stellar flares and ARs, the production mechanisms, similarities and diversities, and their stellar space-weather impacts may be revealed in detail in the near future.

### Electronic supplementary material

Below is the link to the electronic supplementary material.

Movie of Fig. 6 showing X3.4-class flare in AR NOAA 10930. Hinode/SOT/FG Ca ii H image. Courtesy of Joten Okamoto (ISAS/JAXA and NAOJ). (mov 8.37MB)

Movie of Fig. 35 showing 3D magnetic structure and photospheric field *B*_*z*_. Top view (left) and bird's eye view (right). Yellow and blue field lines denote the field lines passing by the current sheet between the two arcades. (mp4 1.10 MB)

Movie of Fig. 44 showing 3D numerical simulations of the four representative types of flare-productive ARs, as introduced in Fig. 17. (Top) Surface vertical magnetic fields (magnetogram). (Bottom) Magnetic field lines. The green field lines are for the parasitic tube and the parallel tube. (mp4 2.94 MB)

Movie of Fig. 54 showing BBSO/GST TiO images of the 2011 July 2 C7.4 flare, clearly showing the formation of penumbra. (mpeg 2.2MB)

Fig. 59 showing BBSO/GST photospheric TiO images. The yellow box shows the rotating sunspots. (mov 15MB)

Movie of Fig. 3 showing “textbook” flux emergence in AR NOAA 12401 observed simultaneously by Hinode, IRIS, and SDO (2015 August 19). From top left to bottom right are the IRIS slit-jaw image of 1400 Å, raster-scan intensitygram at the Mg ii k line core (k3: 2796 Å), intensitygram at the Mg ii triplet line (2798 Å), Dopplergram produced from the Si iv 1403 Å spectrum (blue, white, and red correspond to −10, 0, and +40 km s^−1^, respectively), SDO/AIA 1600 Å and 1700 Å, and SDO/HMI magnetogram and intensitygram. The white arrow in the top left panel indicates the direction of the disk center. (mpeg 24.0MB)

## Appendix: Original advocates of the kink instability

Little has been known about who first proposed the helical kink instability as the formation mechanism for the $\delta$-spots. Almost certainly, it is Linton et al. (1996) who for the first time investigated this instability in the context of $\delta$-spot formation. However, the authors did not clearly claim in their paper that they were the first to propose this idea. Before that, from the observed proper motions of $\delta$-spots, Tanaka (1991) suggested in his posthumous publication that the rising of a knotted twisted flux tube creates the $\delta$-spots (twisted knot model). Although his illustration, adopted as Fig. 15a in this review, is highly evocative of the kink instability, the term “kink instability” was not used at all in his paper. It is now almost impossible to find out whether Katsuo Tanaka and his longtime collaborator Harold Zirin held an idea of the kink instability at that time because both of them are deceased. Here we show a brief history between Tanaka (1991) and Linton et al. (1996), which George H. Fisher, Mark G. Linton, and Yuhong Fan gave to us.

While Fisher was working on the thin flux tube model (Sect. 2.1.1) with Fan in the University of Hawaii, he conceived an idea to add magnetic twist to the thin flux tube, inspired by Mouschovias and Poland (1978), who proposed magnetic twist as a driver of the flux rope eruption. Although the concept of Mouschovias and Poland (1978) was based more on the lateral kink instability, in which the displacement of a twisted flux tube is in a single perpendicular direction (i.e., an $\varOmega$-loop in a 2D plane), Fisher had the misconception that the driving mechanism was the helical kink mode, where the direction of the displacement rotates along the tube axis (see Priest 2014, Sect. 7.5.3 for the two modes). When Fisher and Linton started working on this issue after Fisher moved to the University of California, Berkeley in 1992, Dana W. Longcope showed that the thin flux tube model cannot represent the helical kink instability because this model, in principle, assumes that any physical value is uniform over the tube’s cross section and thus does not include internal motion within the tube.

In the meantime, they studied the textbook of Zirin (1988), especially on the “island $\delta$” sunspot,^10^ as well as the seminal work by Tanaka (1991). These two publications stimulated them to propose the helical kink instability as the origin for the island $\delta$-spot. Over 1994 and 1995, Linton performed energy principle analysis on the instability with Longcope and, eventually, the work resulted in Linton et al. (1996). Moreover, the presentation by Linton et al., probably in the 26th AAS/SPD meeting in 1995, evolved into a collaboration with Russell B. Dahlburg on MHD simulations, which was published later as Linton et al. (1998).

Therefore, it is not easy to narrow down the originator of the idea to one. However, the above story should be a good example of a coincidental misconception serendipitously producing fruit.

## Footnotes

1 The GST was formerly called the New Solar Telescope (NST).

2 This is why a study report on the future of solar physics, published by the Next Generation Solar Physics Mission (NGSPM)’s Science Objectives Team (SOT), chartered by NASA, JAXA, and ESA, cites the formation mechanism of flare-productive ARs as one of the most important science targets. At the time of this writing, the report is available at https://hinode.nao.ac.jp/SOLAR-C/SOLAR-C/Documents/NGSPM_report_170731.pdf. Also, observation and modeling of such ARs is recognized as an important target in the International Space-weather Roadmap (Schrijver et al. 2015).

3 Millionths of the solar hemisphere. $1\, \mathrm{MSH}\sim 3\times 10^{6}\, \mathrm{km}^{2}$.

4
McIntosh (1990) mentioned that “[r]arely will the measured magnetic class conflict with” his definitions of unipolar and bipolar groups.

5 It is implicitly assumed here that the net helicity flux through *S* other than the photosphere is zero.

6 It is also suggested that the flux ropes emerge bodily from below the surface (e.g., Lites et al. 1995; Okamoto et al. 2008).

7 Not to be confused with the magnetic helicity density, ${{\mathbf {B}}}\cdot {{\mathbf {A}}}={{\mathbf {A}}}\cdot (\nabla \times {{\mathbf {A}}})$: see Sect. 3.2.3.

8 Note that the kink instability is also suggested as one driving mechanism of CME eruption: see Sect. 2.2.

9 In local thermodynamical equilibrium (LTE), it is assumed that the state of plasma is described simply by the Saha–Boltzmann equations, i.e., as a function of the local kinetic temperature and electron density alone. Non-LTE indicates that this assumption is not valid.
